# Enhanced alkene productivity in methanol-to-hydrocarbons conversion using a secondary catalyst component and hydrogen co-feeds

**DOI:** 10.1038/s41467-026-77944-9

**Published:** 2026-09-23

**Authors:** Matteo Vanni, Andreas Brenig, Adam H. Clark, Jeroen A. van Bokhoven, Vladimir Paunović

**Affiliations:** 1 PSI Center for Energy and Environmental Sciences, Paul Scherrer Institute, Villigen, Switzerland; 2 Institute for Chemical and Bioengineering, Department of Chemistry and Applied Biosciences, ETH Zurich, Zurich, Switzerland; 3 PSI Center for Photon Science, Paul Scherrer Institute, Villigen, Switzerland

**Keywords:** Heterogeneous catalysis, Chemical engineering, Catalyst synthesis

## Abstract

The methanol-to-hydrocarbons conversion over unidimensional 10-membered ring zeolites offers an attractive route to produce C_3_–C_5_ alkenes, central building blocks for the production of sustainable aviation fuels. However, the application of such zeolites is limited by a rapid deactivation due to coke formation. We present a novel catalyst design concept to significantly enhance coke resistance, achieved by physically mixing the zeolite with a secondary catalyst component exhibiting hydrogenation activity and operating under near ambient-pressure hydrogen co-feeds. Among the tested materials, Pd/SiO_2_ provides the highest stability enhancement, leading up to 10× higher cumulative productivity of C_3_–C_5_ alkenes, and outperforming a bifunctional Pd-loaded zeolite. The enhancement is observed across a broad range of hydrogen concentrations and zeolite:Pd/SiO_2_ weight ratios, offering flexibility to minimize hydrogen and Pd use. Kinetic and spectroscopic investigations reveal that Pd intercepts coke-forming intermediates, such as formaldehyde and dienes, thereby reducing the rate of internal and external coke deposition.

## Abstract (web summary)

The methanol-to-hydrocarbons conversion over unidimensional 10-membered-ring zeolites provides an attractive route for producing C_3_–C_5_ alkenes but is limited by rapid catalyst deactivation caused by severe coke formation. Here, the authors introduce a novel catalyst design strategy based on physically mixing the zeolite with a secondary catalyst component and using hydrogen co-feeds, markedly enhancing coke resistance.

## Introduction

The methanol-to-hydrocarbons (MTH) conversion, catalyzed by zeolites and zeotypes, is an important industrial technology for the production of light alkenes (C_2_-C_4_) and gasoline-range fractions. Product selectivity is primarily governed by the shape-selective properties of the catalyst, and several process variants are commercialized to meet specific market demands^1–4^. The MTH reaction proceeds via a complex mechanism, involving methylation and cracking of alkene and aromatic intermediates constituting the hydrocarbon-pool (HCP)^1,3–5^. Reactions in the alkene cycle primarily yield propene and higher alkenes, while reactions in the aromatic cycle produce aromatics, ethene, and, to a certain extent, propene. Hydrogen transfer, cyclization, and cracking reactions interconnect the two cycles. Over time, HCP intermediates evolve into higher-molecular-weight species that remain within the catalyst pores and over the crystal surfaces, forming the so-called coke deposits.

The MTH process holds significant promise for decarbonizing the chemical industry and transportation sectors, since methanol can be produced from various carbon feedstocks, including renewable ones, such as biomass and CO_2_. Because of this, MTH has been recognized as an attractive step for the production of sustainable aviation fuels (SAFs), which exhibit a high energy density that is difficult to meet with batteries or other hydrogen carriers, such as ammonia^6–8^. In this scenario, MTH provides a pathway to C_3+_ range alkenes that, via oligomerization and hydrogenation, can be converted to C_8_–C_18_ alkanes, which are the main constituents of jet fuel^9–11^. This imposes specific requirements on the MTH product distribution, since the ideal alkene stream should preferably comprise C_3_–C_8_ alkenes that can be readily oligomerized over acid catalysts^12,13^. Zeolites, such as ZSM-22 (TON framework), ZSM-23 (MTT), and ZSM-48 (RFE), constitute a particularly attractive class of MTH catalysts, since the confinement effect of their one-dimensional 10-memebered ring (1D10-MR) channels favor the production of C_3_–C_5_ alkenes and suppress significantly the formation of aromatics^14–16^. However, this channel geometry substantially limits the molecular diffusion, resulting in a fast coke-induced catalyst deactivation^14,17^. Different zeolite engineering approaches have been attempted to circumvent this issue. For instance, by growing nanosized ZSM-23 (MTT) using tailored organic structure directing agents, considerable lifetime improvements in methanol cumulative turnover capacity (CT_0_) were achieved (from ≈ 1 to ≈ 64 g_CH3OH_ g_cat_^−1^)^18^. However, this bottom-up approach has variable reproducibility^19^, and limited scope due to synthetic challenges^20^. Alternatively, top-down methodologies, such as sequential desilication-dealumination treatments of ZSM-22, provided a significant increase in its CT_0_ by 10–17× (from ≈ 1.8 to ≈ 30 g_CH3OH_ g_cat_^–1^)^21,22^. In addition, substantial enhancement of catalyst stability can be attained by engineering the process conditions. Recently, high-pressure hydrogen (H_2_) co-feeds (30 bar, 10–99 mol% H_2_) were demonstrated as an attractive strategy that can extend lifetimes in MTH by a factor of 3–70× over relevant 3D zeolite topologies, particularly chabazite (CHA), thereby preserving the original alkene selectivity^23^. Further enhancement could be achieved over CHA through the additional co-feeding of H_2_O, leveraging a synergistic effect with H_2_^24^. According to kinetic investigations^25^, the positive effect of H_2_ co-feeds is associated with quenching of key coke precursors, such as formaldehyde (CH_2_O) and dienes, via their hydrogenation over Brønsted acid sites. This raises the question of whether the integration of extra hydrogenation functionality could be leveraged. Early studies of MTH over offretite (OFF) loaded with Ni or Pt, in the presence of H_2_ co-feed at ambient pressure, resulted in a 5× improvement of the CT_0_, but at the cost of a complete hydrogenation of alkenes into alkanes^26^. More recently, a 2× increase in CT_0_ (from ≈ 375 to 800 g_DME_ g_cat_^−1^) was achieved in the conversion of dimethyl-ether (DME) to hydrocarbons over the bifunctional 1D zeolite Pd/ZSM-48, compared to the parent zeolite, with a moderate increase in alkanes formation (≈ 3.5×)^27^. However, the reaction was still performed at high-pressure (20 bar). To date, a catalytic system that preserves the alkene selectivity while operating at low pressure is lacking.

Herein, we present a novel concept to substantially increase the resistance to coking and cumulative C_3_–C_5_ alkenes productivity (CP_C*n*H2*n, n *= 3–5_) of 1D 10-MR ZSM-22 and ZSM-48 zeolites at near-ambient operating pressures that uses H_2_ co-feeding and a secondary catalyst component (SCC) physically mixed with the MTH catalyst. The SCC consists of a supported metal with hydrogenating functionality, thus not requiring any modification of the zeolite itself. Through in-depth catalyst testing, we identify the best-performing supported metal system. By combining kinetic experiments with *operando* X-ray absorption (XAS) and *operando* diffuse reflectance ultraviolet-visible (DR-UV-vis) spectroscopies, we provide insights into the mechanism of the co-catalytic action of SCC.

## Results

### The impact of secondary catalyst components

**Fig. 1:**
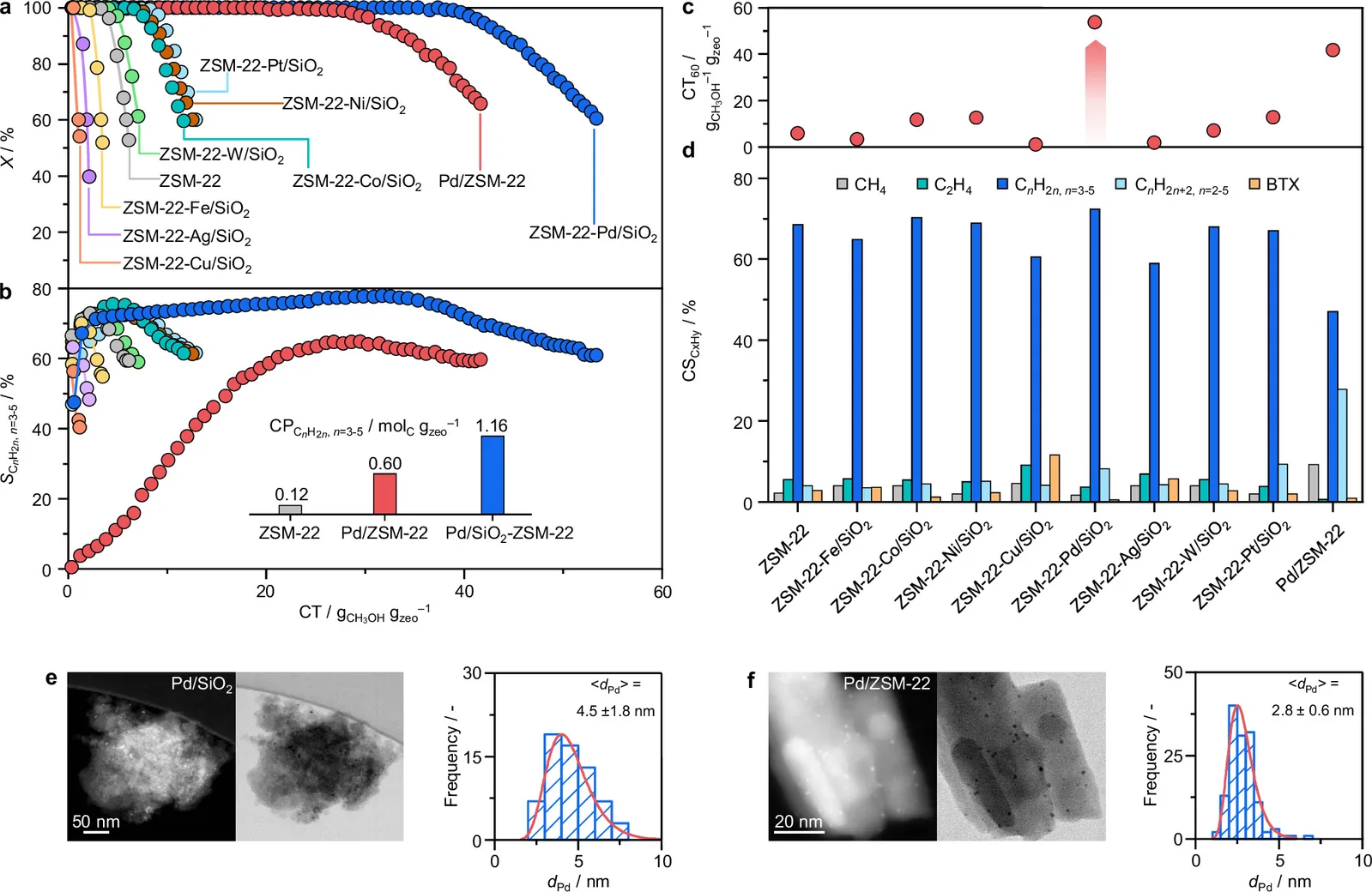
MTH performance of ZSM-22-based multicomponent catalysts (MCCs) with different secondary catalysts components (SCCs). **a** Methanol conversion (*X*) and **b** selectivity to C_3_–C_5_ olefins (S_C*n*H2*n*, *n* = 3–5_) vs. methanol cumulative turnover (CT) of the parent ZSM-22, ZSM-22-M/SiO_2_ MCC (M = Fe, Co, Ni, Cu, Pd, Ag, W, Pt), and bifunctional Pd/ZSM-22. The inset in **b** shows the cumulative productivity of C_3_–C_5_ olefins (CP_C*n*H2*n*, *n* = 3–5_) for selected systems. **c** Methanol cumulative turnover capacity (CT_60_) and **d** cumulative selectivities to specific hydrocarbons (CS_CxHy_) of the parent zeolite and MCCs. Scanning transmission electron microscopy (STEM) images and Pd particle size analysis of **e** Pd/SiO_2_, and **f** Pd/ZSM-22. Reaction conditions: CH_3_OH:H_2_ = 10:90 mol%, WHSV = 2.4 g_CH3OH_ g_zeo_ h^–1^, *W*_SCC_ :*W*_zeolite_ = 1, *T* = 723 K, and *P* = 1.2 bar.

To investigate the influence of the external hydrogenating function on the MTH conversion, a series of multicomponent catalysts (MCCs) was prepared by mixing a commercial ZSM-22 zeolite with secondary catalyst components (SCCs), consisting of transition metals deposited over an inert silica (SiO_2_) support, in 1:1 wt% ratio (Supplementary Figs. 1 and 2 and Supplementary Table 1). The SCCs were synthesized with a nominal metal loading of 67 µmol g_SiO2_^–1^ (0.4–1.2 wt%, depending on the metal) by either incipient-wetness impregnation or ion-adsorption, followed by calcination. The absence of peaks of respective metal or metal oxides in the powder X-ray diffractograms of M/SiO_2_ SCC materials indicate a high metal dispersion, except for Pt/SiO_2_, exhibiting larger metal nanoparticles of ≈ 15 nm (Supplementary Fig. 2). In line with previous reports on ZSM-22^14^, MTH conversion tests were performed at a relatively low weight hourly space velocity (WHSV) of 2.4 h^–1^, moderate temperature of *T* = 723 K, total pressure of *P* = 1.2 bar, and in the presence of H_2_ co-feed (CH_3_OH:H_2_ = 10:90 mol%). The impact of SCCs in MCCs was assessed based on their methanol cumulative turnover capacity, CT_60_, measured from the initially complete methanol conversion (*X* = 100%, corresponding to industrial operation) down to *X* = 60%, and the cumulative selectivities to hydrocarbon products, CS_CxHy_, in the specified conversion range (*X* = 100–60%) (Fig. 1a–d). Among the studied systems, the CT_60_ of the pristine ZSM-22 (5.9 g_CH3OH_ g_zeo_^–1^) was enhanced in the MCCs incorporating Ni/SiO_2_ (13 g_CH3OH_ g_zeo_^–1^), Co/SiO_2_ (12 g_CH3OH_ g_zeo_^–1^), Pt/SiO_2_ (13 g_CH3OH_ g_zeo_^–1^), and, most notably, Pd/SiO_2_ (54 g_CH3OH_ g_zeo_^–1^), all of which comprise a metal with hydrogenation activity. Furthermore, SCCs containing Co, Ni, Pd, Pt lead to low selectivity to benzene, toluene, and xylene (BTX) aromatics, with the lowest values attained over Pd/SiO_2_ (CS_BTX_ = 0.5%), and high selectivity to C_3_–C_5_ alkenes, CS_C*n*H2*n*, *n* = 3–5_, (72%), which is comparable to the product distribution pattern of ZSM-22 (CS_BTX_ = 2.8%, CS_C*n*H2*n*, *n *= 3–5_ = 69%). Notably, among the investigated SCCs, Pd/SiO_2_ exhibits the highest selectivity to C_3_–C_5_ alkenes and the highest CT_60_, thus providing the greatest enhancement of the productivity of C_3_–C_5_ alkenes. Contrastingly, Cu/SiO_2_ and Ag/SiO_2_ SCCs lead to the lowest CT_60_ values (1.9 g_CH3OH_ g_zeo_^–1^), a higher selectivity to BTX (12% and 5.7%), and reduced selectivity to C_3_–C_5_ alkenes (59% and 61% vs. 69%) compared to the parent zeolite. This trend indicates a higher contribution of the aromatic cycle and, consequently, enhanced coke formation, consistent with the low CT_60_ achieved over these SCCs.

### The activity of Pd/SiO_2_ SCC

In view of its exceptional promoting effect, the influence of Pd/SiO_2_ SCC is analyzed in more detail. Scanning transmission electron microscopy (STEM) imaging shows homogeneously distributed Pd nanoparticles dispersed on the SiO_2_ surface, with a size distribution centered at *d*_Pd_ ≈ 4.5 nm (Fig. 1e and Supplementary Fig. 3). A closer inspection at the evolution of *S*_C*n*H2*n*, *n* = 3–5_ vs. CT over ZSM-22-Pd/SiO_2_ MCC (Fig. 1b and Supplementary Fig. 4) reveals the existence of a short induction period, characterized by an initially high selectivity to C_2_–C_5_ alkanes, which is followed by a rapid increase in selectivity to the C_3_–C_5_ alkene fraction to a level comparable to that of the parent material (≈ 70%). These product distribution profiles suggest initial unselective alkene hydrogenation to alkanes over Pd. After this transient period, the MCC becomes alkene-selective, and the *S*_C*n*H2*n*, *n* = 3–5_ profile follows a trend qualitatively similar to that of the parent ZSM-22. The selectivity to alkenes gradually increases with time until reaching a maximum of *S*_C*n*H2*n*, *n* = 3–5_ = 78%, just before the methanol conversion drops below 100%. This trend reflects the changing selectivity of individual alkenes, with C_5_H_10_ increasing markedly from 6 to 23% at the point where conversion starts to decline (Supplementary Fig. 4). The increase in selectivity to higher alkenes up to the point of drop in conversion is rationalized with the progressively decreasing extent of secondary cracking reactions occurring in the lower sections of the catalyst bed^28,29^. Considering the rather short duration of the induction period, the CS_C*n*H2*n*, *n *= 3–5_ of ZSM-22-Pd/SiO_2_ MCC and ZSM-22 are very similar (Fig. 1b). Nonetheless, the MCC under H_2_ co-feeding exhibits a reduced CS_C2H4_ (3.6% vs. 5.4%) and CS_BTX_ (0.5% vs. 2.8%). Since ethene products arise from the aromatic cycle of the HCP^30^, this result points to a suppression of this cycle over the MCC under H_2_ co-feeding. The higher CS_C*n*H2*n+2*, *n* = 2–5_ (8.2% vs. 4.0%) of the MCC likely originates from the hydrogenation of alkene products over the Pd sites, rather than from hydrogen transfer, typically associated with cyclization and aromatization. Overall, the use of an SCC and the presence of H_2_ co-feeds enhances the cumulative productivity of C_3_–C_5_ alkenes by up to 10× (Fig. 1a, inset).

### The impact of the proximity between Pd and zeolite active sites

The influence of metal and zeolite proximity was analyzed by assessing the performance of three specific catalysts, each containing the same nominal amount of Pd: (1) MCC in which Pd/SiO_2_ and ZSM-22 particles are separately sieved and then mixed in a catalyst bed (*vide supra*), (2) MCC in which Pd/SiO_2_ and ZSM-22 powders are closely mixed by grinding, pressed and sieved, and (3) a bifunctional Pd/ZSM-22 catalyst (Fig. 1f and Supplementary Figs. 1 and 5), comprising Pd loaded on a ZSM-22 zeolite and exhibiting Pd particles within a similar size range (*d*_Pd_ ≈ 2.8 nm), representing the highest proximity between the active metal and zeolite sites, pressed and sieved. Notably, the integration of Pd in a separate secondary phase (i.e., the SCC) is more effective than loading the metal directly on the zeolite (see Fig. 1a–d). In particular, the bifunctional Pd/ZSM-22 catalyst not only deactivated more rapidly (CT_60_ = 43 vs. 54 g_CH3OH_ g_zeo_^–1^) but also exhibited a stronger hydrogenation activity. This resulted in a much higher CS to alkanes (CS_C*n*H2*n*+2, *n *= 2–5_ = 28 vs. 8%) and a more prominent lag time for the development of the C_3_–C_5_ alkene fraction, with a markedly peak-shaped *S*_C*n*H2*n*, *n*=3–5_ profile. As a result, although an enhancement in alkene productivity was observed over Pd/ZSM-22, the MCC achieved twice the productivity under the same conditions (CP_C*n*H2*n*, *n*=3–5_ = 0.60 vs. 1.2 mol_C_ g _zeo_^–1^ of the MCC). Pd/ZSM-22 exhibits comparable textural properties as the parent ZSM-22 (Supplementary Table 1 and Supplementary Fig. 6), but a lower concentration of strong acid sites (by 23%). Considering the smaller average Pd nanoparticle size in Pd/ZSM-22 compared to Pd/SiO_2_ SCC (2.8 vs. 4.5 nm), which is expected to enhance the hydrogenation activity, its inferior performance in terms of over-hydrogenation and catalyst stability relative to the MCC suggests that the close proximity of Pd centers to HCP active species is not optimal for leveraging the beneficial impact of the hydrogenating functionalities.

No pronounced differences were observed between the two MCC catalysts with different ZSM-22-SCC proximity in terms of turnover capacity and product selectivity, with a slightly slower deactivation of the grinded MCC at sub-complete conversion (Supplementary Fig. 7). This indicates that the positive effect is preserved when the average SCC-zeolite distance is reduced from tens of micrometers (mixed particles) to below 1 µm (mixed powders) and further suggests that this effect primarily arises from the interception of deactivating gas-phase species by the SCC. Notably, since SiO_2_ is a common binder in technical catalysts, the possibility of mixing Pd/SiO_2_ and zeolite as powders holds promise for the future development of shaped MCC catalysts^31,32^.

### The impact of SCC on another zeolite framework and the influence of SCC loading

**Fig. 2:**
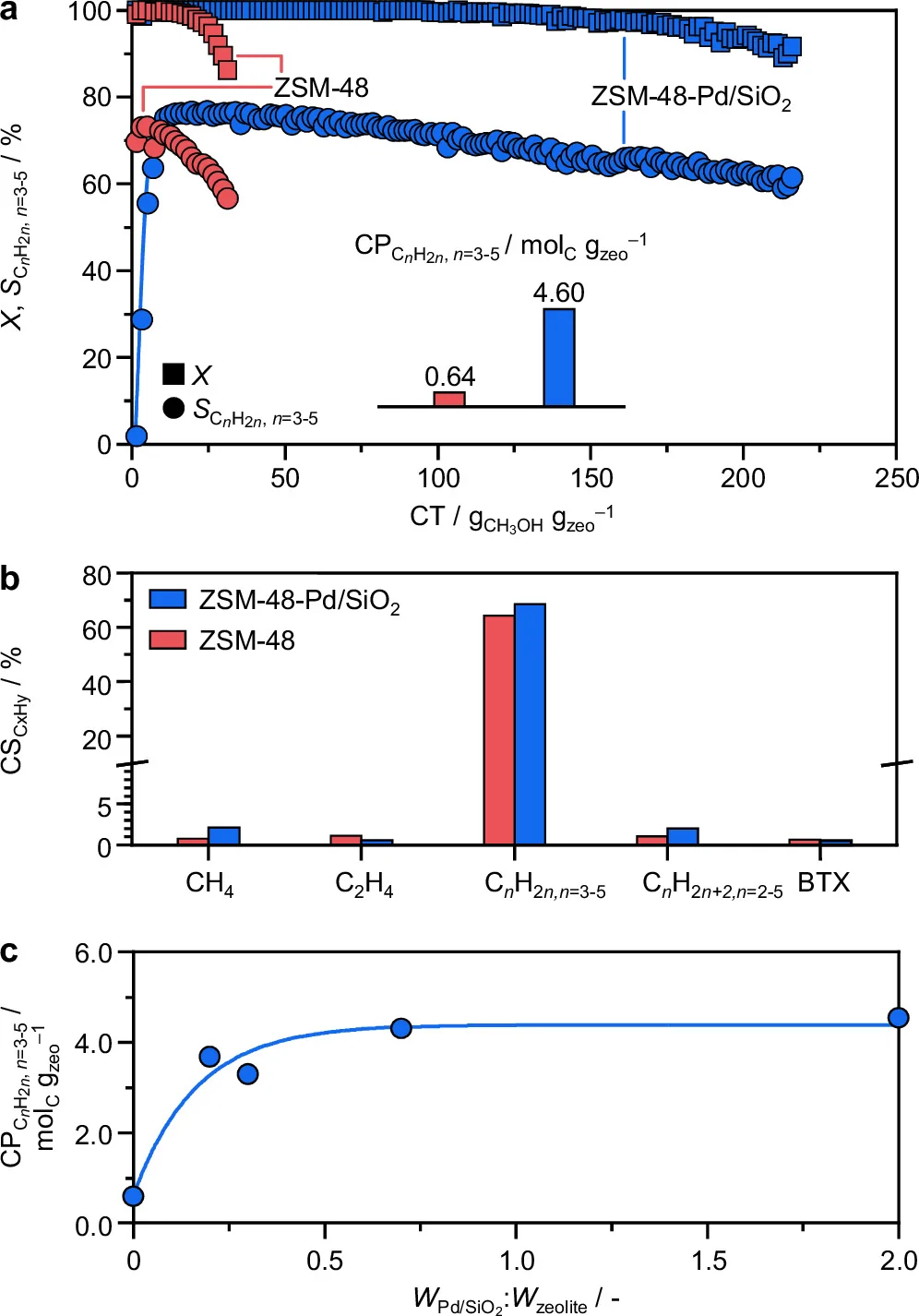
MTH performance of ZSM-48-Pd/SiO_2_ MCC and influence of the SCC loading. **a** Methanol conversion (*X*) and selectivity toward C_3_–C_5_ alkenes (*S*_C*n*H2*n*_, _*n* = 3–5_) vs. methanol cumulative turnover (CT), and **b** cumulative selectivities to specific hydrocarbons (CS_CxHy_) of the parent ZSM-48 and ZSM-48-Pd/SiO_2_ MCC. The inset in **a** shows the cumulative productivity of C_3_–C_5_ alkenes (CP_C*n*H2*n*, *n* = 3–5_). **c** Cumulative productivity of C_3–5_ alkenes (CP_C*n*H2*n*, *n *= 3–5_) of ZSM-48-Pd/SiO_2_ MCC, assessed at MTH conversion *X* = 100–90%, vs. the Pd/SiO_2_:zeolite weight ratio (*W*_Pd/SiO2_:*W*_zeolite_). Reaction conditions: CH_3_OH:H_2_ = 10:90 mol%, WHSV = 4.8 g_CH3OH_ g_zeo_ h^–1^, *W*_Pd/SiO2_:*W*_zeolite_ = 2 (**a**, **b**) or 0–2 (**c**), *T* = 723 K, and *P* = 1.2 bar.

To evaluate the generality of the approach demonstrated with ZSM-22, we studied the impact of SCC on the performance of a commercial ZSM-48 (*W*_Pd/SiO2_:*W*_zeolite_ = 2, Supplementary Fig. 1 and Supplementary Table 1), exhibiting a disordered RFE topology with 1D 10-MR straight channels slightly wider than in ZSM-22 (5.6 × 5.3 Å vs. 5.7 × 4.6 Å). Under suitable WHSV and temperature, ZSM-48 is more stable and selective to higher alkenes than ZSM**-**22^14^. The use of H_2_ co-feeds instead of Ar slightly improves the CP_C*n*H2*n*, *n* = 3–5_ of parent ZSM**-**48 by 1.6× (Supplementary Fig. 8). The addition of Pd/SiO_2_ SCC in the absence of H_2_ co**‑**feeding leads to higher enhancement by ≈ 4×, likely because small amounts of H_2_ are always generated via minor CH_3_OH decomposition on Pd (Supplementary Fig. 9). External H_2_ co**-**feeding is required to maximize productivity enhancement. Compared to the parent ZSM-48 tested with H_2_, the combined use of SCC and H_2_ co-feed increased the alkene productivity in the conversion range *X* = 100–90% by ≈ 7× (Fig. 2a, b). The enhancement factor reaches ≈ 12× when the parent zeolite without H_2_ co**-**feed is used as the benchmark (Supplementary Fig. 8). Furthermore, the beneficial effect of the SCC and H_2_ co-feed is also observed at elevated inlet CH_3_OH concentrations (Supplementary Fig. 10). Compared to ZSM-22, where the hydrocarbon distribution is dominated by C_3_H_6_, over ZSM-48, C_3_H_6_:C_4_H_8_:C_5_H_10_ are produced in a ratio of ≈ 1.0:0.8:1.0 mol_C_% (Supplementary Fig. 11). As observed in ZSM-22, the selectivity profiles of MCC follow similar trends to those of the parent zeolite ZSM-48 but extend over a much higher range of CT. Similarly to ZSM-22, the selectivity profiles reveal a short induction period at the early stage of the reaction, characterized by extensive CH_4_ and alkane formation (Supplementary Fig. 11). This suggests methanol decomposition over Pd to carbon monoxide and hydrogen, followed by hydrogenation into CH_4_, as well as unselective alkene hydrogenation to alkanes (vide infra; Supplementary Fig. 9). Compared to the parent ZSM-48, the MCC shows a lower CS_C2H4_ (0.6 vs. 1.1%), while the selectivity toward aromatic products is low for both catalysts (CS_BTX_ < 0.6%).

These results demonstrate the general applicability of the MCC approach as a simple and effective strategy to extend alkene productivity across different 1D 10-MR zeolites. The enhancement results from a higher methanol conversion capacity combined with a retained product selectivity. In this regard, a methanol cumulative turnover capacity of CT_0_ = 310 g_CH3OH_ g_zeo_^–1^ is obtained up to full catalyst deactivation. This value is in the range of those achieved using the industrially deployed ZSM-5 zeolite (Supplementary Fig. 12), illustrating the potential of the MCC approach to attain industrially attractive catalyst stability with 1D zeolite topologies. Furthermore, as demonstrated with the Pd/SiO_2_-ZSM-5 MCC system, which showed an enhancement factor of 1.5× compared to the already high CT_0_ value of the parent ZSM-5 catalyst (Supplementary Fig. 13), the MCC approach can be extended beyond 1D 10**-**MR zeolites.

The ZSM-48-Pd/SiO_2_ MCC system was used to investigate the influence of the loading of SCC (and thus of Pd). In the conversion range between *X* = 100–90%, CP_C*n*H2*n*, *n* = 3–5_ increases with the Pd/SiO_2_ content as a result of the increased CT_90_, following an asymptotic trend (Fig. 2c). In particular, while a CP_C*n*H2*n*, *n* = 3–5_ of 0.64 mol_C_ g_zeo_^–1^ is attained over the parent zeolite, the MCC with *W*_Pd/SiO2_:*W*_zeolite_ = 0.2 results in a CP_C*n*H2*n*, *n* = 3–5_ of 3.7 mol_C_ g_zeo_^–1^ (selectivity vs. conversion profiles in Supplementary Fig. 14), and the MCC with much higher *W*_Pd/SiO2_:*W*_zeolite_ = 2 yields a CP_C*n*H2*n*, *n* = 3–5_ of 4.6 mol_C_ g_zeo_^–1^. This finding has major practical implications, as a relatively low amount of SCC is needed to achieve maximal productivity, limiting the amount of precious metal. From a fundamental perspective, the trends observed with regard to the primary influence of SCC loading and Pd speciation on the CP_C*n*H2*n, n* = 3–5_ values, suggest that the impact of SCC primarily stems from the hydrogenation of species that are active in coke formation.

### The impact of SCC on the formation of coke

**Fig. 3:**
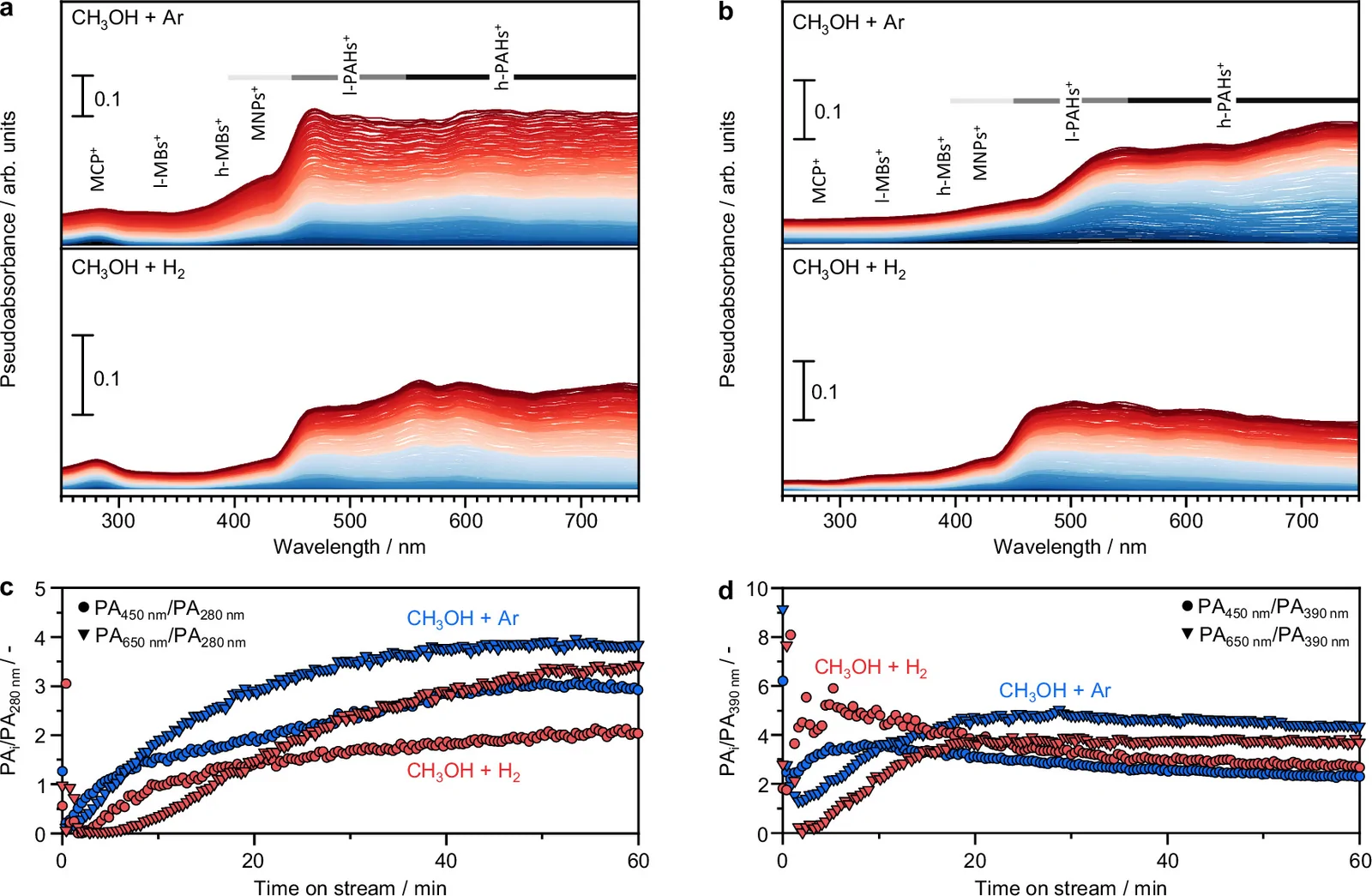
DR-UV-vis study of intermediates in the MTH reaction over MCC. **a**, **b** DR-UV-vis spectra acquired during MTH reaction and **c**, **d** temporal evolution of the normalized pseudoabsorbance (PA) for selected bands in the presence and in the absence of a H_2_ co-feed over ZSM-22-Pd/SiO_2_ (**a**, **c**) and ZSM-48-Pd/SiO_2_ (**b**, **d**) MCCs, without and with H_2_ co-feed. Reaction conditions: CH_3_OH:H_2_:Ar = 17:83(0):0(83) mol%, WHSV = 2.9 (ZSM-22-Pd/SiO_2_) and 5.2 g_CH3OH_ g_zeo_ h^–1^ (ZSM-48-Pd/SiO_2_), *W*_Pd/SiO2_:*W*_zeolite_ = 0.2, *T* = 723 K, and *P* = 1.0 bar.

The findings on the influence of SCC on the zeolite lifetime were complemented with an *operando* DR-UV-vis spectroscopic analysis of ZSM-22-Pd/SiO_2_ and ZSM-48-Pd/SiO_2_ MCCs in the MTH reaction in the absence (i.e., using argon as diluent) or presence of H_2_ co-feeds and other conditions closely matching those applied in the fixed-bed reactor tests (Fig. 3a, b). Qualitatively similar DR-UV-vis spectra are observed over the ZSM-22-Pd/SiO_2_ both under Ar and H_2_ co-feeding. The initial spectra show a characteristic band at 280 nm at the onset of the reaction (Fig. 3a)^33,34^, attributed to a cyclopentenyl cation (MCP^+^) with 8-9 carbon atoms and H-substituted on the C-2 position^35^. This is in agreement with previous studies identifying cyclopentenyl species as active HCP intermediates in ZSM-22^36–39^. In particular, alkyl-cyclopentenyl cations are easily formed from alkene species, even in the absence of CH_3_OH (Supplementary Fig. 15), and can in turn revert to alkenes or evolve into aromatics without accumulating. With the progress of reaction, the bands at ≈ 430 nm, ascribed to methylnaphthalenium ions (MNPs^+^) and *≈* 470 nm, associated with lower polyaromatic hydrocarbons (l-PAHs^+^) evolve. The high-wavelength region (>550 nm), which comprises contributions from higher polycyclic aromatic hydrocarbons (h-PAHs^+^)^33,34^, rises progressively at a rate comparable to that of the MNPs^+^ band. Previous theoretical calculations and extraction experiments indicated that MNPs^+^, as well as l-PAHs^+^ with pseudo-linear geometry (e.g., anthracene, fluorene) grow within and block the ZSM-22 micropores^40,41^, thus the band at 450 nm, intermediate between 430 and 470 nm, is considered representative of internal coke. Conversely, bulkier h-PAHs are attributed to external coke, of which the band at 650 nm is chosen as representative. A semi-quantitative assessment of the H_2_ influence on internal and external coke formation, relative to the active HCP intermediates MCP^+^, can be made by comparing the evolution of the pseudo-absorbance (PA) ratios PA_450 nm_/PA_280 nm_ and PA_650 nm_/PA_280 nm_ (Fig. 3c). For both coke types, the presence of H_2_ results in a decreased accumulation rate. While external coke formation is initially suppressed under H_2_ (PA_650 nm_/PA_280 nm_ ≈ 0), its relative intensity eventually rises to values comparable to those observed under argon. In contrast, PA_450 nm_/PA_280 nm_, reflecting the major source of micropore blockage in ZSM-22, remains lower in the presence of H_2_. Spectra of ZSM-48-Pd/SiO_2_ reflect a different HCP composition than in ZSM-22, with a less evident MCP^+^ signature (Fig. 3b). In this regard, prior studies on the MTH mechanism over ZSM-48 had proposed higher methylated benzenes (h-MBs^+^, ≈ 390 nm) as the most relevant intermediates, consistent with the larger pore dimensions (5.3 × 5.6 Å)^42^. Nevertheless, no distinct peaks can be observed in this convoluted region, and the bands corresponding to MNPs^+^ and PAHs^+^ are the most prominent. Comparison of the PA profiles in the high-wavelength region suggests that H_2_ co-feeding leads to a reduced contribution from higher PAHs^+^. The PA ratios PA_450 nm_/PA_390 nm_ and PA_650 nm_/PA_390 nm_ are plotted in the presence and absence of a H_2_ co-feed, using the 390 nm band (h-MBs^+^) as representative of active intermediates (Fig. 3d). The PA_650 nm_/PA_390 nm_ profile indicates a reduced formation of h-PAHs^+^ in the presence of H_2_ compared to argon. The ambiguous trend of PA_450 nm_/PA_390 nm_ could reflect that the concentration of h-MBs^+^ at 390 nm is also diminished in the presence of H_2_ and Pd, in line with the lower selectivity to ethene and BTX, leading to a less predictable evolution of the PA_450 nm_/PA_390 nm_ ratio over time. In summary, *operando* DR-UV-vis analysis indicates that ambient pressure H_2_ co-feeding greatly reduces the rate of external and, at least for ZSM-22, internal coke growth, with respect to active HCP intermediates. A reduced rate of coke deposition over the MCC system is also confirmed by the independent quantification of the coke content in spent ZSM**-**48 and ZSM**-**Pd/SiO_2_ MCC catalysts by thermogravimetric analysis (TGA, Supplementary Fig. 10).

**Fig. 4:**
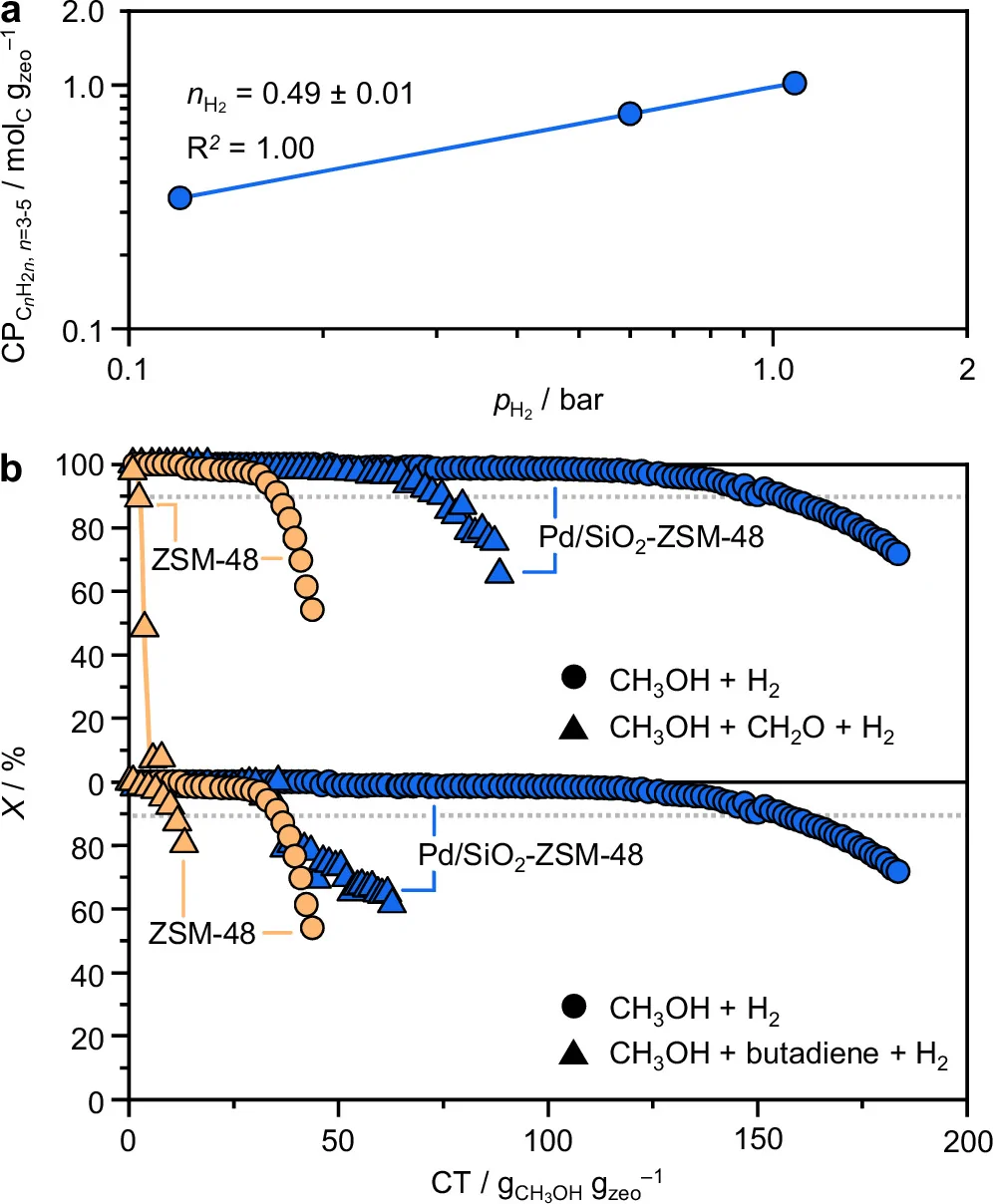
The influence of SCC and H_2_ co-feed on coke-induced deactivation. **a** The cumulative productivity of C_3_–C_5_ alkenes (CP_C*n*H2*n*, *n* = 3–5_) over ZSM-22-Pd/SiO_2_ MCC vs. the partial pressure of H_2_ (*p*_H2_). Reaction conditions: CH_3_OH:H_2_:Ar = 10:10-90:80-0, WHSV = 2.4 g_CH3OH_ g_zeo_ h^–1^, *W*_Pd/SiO2_:*W*_zeolite_ = 2, *T* = 723 K, and *P* = 1.2 bar. **b** Methanol conversion (*X*) vs. methanol cumulative turnover (CT) of parent ZSM-48 and ZSM-48-Pd/SiO_2_ MCC in the MTH reaction with and without CH_2_O or butadiene co-feeds (2 mol_C_% vs. CH_3_OH). Butadiene was generated in situ by co-feeding 3-buten-1-ol. Gray dotted lines highlight the conversion level *X* = 90% at which CT was compared. Reaction conditions: CH_3_OH:co-feed:H_2_ = 10(9.8):0(0.2):90 mol%, WHSV = 4.8 g_CH3OH_ g_zeo_ h^–1^, *W*_Pd/SiO2_:*W*_zeolite_ = 0.2, *T* = 723 K, and *P* = 1.2 bar.

A reduced rate of coke-forming sequences in MCC under H_2_ co-feeds suggests the interception of the reaction intermediates causing coking. To better understand such a mechanism, we first studied the impact of H_2_ concentration in the reactant stream on the stability and product distribution. In these experiments, the CH_3_OH concentration in the feed was kept constant at 10 mol%, while the H_2_ concentration was varied, with Ar used as balance gas (CH_3_OH:H_2_:Ar = 10:10-90:80-0 mol%). The methanol cumulative turnover capacity, measured from initial complete conversion down to *X* = 90%, CT_90_, increases prominently with increasing the H_2_ partial pressure, but levels off at moderate H_2_ pressures, corroborating the hypothesis that the enhancement in catalyst stability stems primarily from the hydrogenation activity of the SCC (Supplementary Fig. 16). Cumulative product selectivities are virtually identical over the MCC system within the concentration range *c*_H2_ = 10–90 mol%, although a small and progressive decrease in the selectivity to BTX aromatics (from 1.7% to 0.5%) is observed with increasing H_2_ concentration. Consequently, CP_C*n*H2*n*, *n* = 3–5_ values mirror the catalyst lifetime sequence (Fig. 4a). Notably, the CP_C*n*H2*n*, *n* = 3–5_ trend attained over MCC exhibits the apparent reaction order with respect to H_2_ of *n*_H2_ = 0.49, which is characteristic for the kinetics of hydrogenation reactions in which hydrogenation is the rate-limiting step. Assuming that CP_C*n*H2*n*, *n *= 3–5_ is proportional to the rate at which deactivating species are removed during the MTH reaction, thereby extending the number of catalytic turnovers, these results suggest that the effect of SCC primarily arises from intercepting coke-forming pathways through hydrogenation of coke precursors (Supplementary Methods 3). With regard to this, CH_2_O, formed as a primary MTH product, and hydrocarbons with increased unsaturation degree, such as dienes and polyenes, are suggested as central intermediates favoring the production of aromatics and coke^34,43–46^. Recent theoretical and kinetic investigations showed that hydrogenation of these molecules over BAS using high-pressure H_2_ co-feeds can intercept the coke-forming pathways and thus increase the catalyst lifetime^25^. To gain insights into the nature of species potentially hydrogenated over the SCC, low-concentrations of CH_2_O (2.0 mol_C_%) and butadiene (generated in situ by co-feeding 3-buten-1-ol, 7.0 mol_C_%) were individually added to the CH_3_OH:H_2_ = 10:90 mol% feed over ZSM-48 and ZSM-48-Pd/SiO_2_ MCC (Fig. 4b). CT_90_ values in the presence of CH_2_O decrease 15× for ZSM-48, and only 2× for MCC, while with butadiene the decrease is 3× for ZSM-48 and ≈ 4× for MCC, yet CT_90_ remains higher for the MCC compared to the parent zeolite. This observation points to a higher efficacy of the MCC in quenching CH_2_O, consistently also with the CT_90_ achieved in the presence of this co-feed compared to butadiene. Remarkably, in the excess of both deactivating species, a net improvement in the zeolite performance is observed upon integration of Pd/SiO_2_ within the catalyst formulation. Particularly, the MCC (*W*_Pd/SiO2_:*W*_zeolite_ = 0.2) allows to match (with butadiene) or even improve (2×, with CH_2_O) the CT_90_ of the parent ZSM-48, although without achieving the performance of the MCC system in the absence of high concentrations of deactivating co-reactants. Similar results were obtained in the experiments with CH_2_O co-feeding over ZSM-22 and the corresponding MCC (Supplementary Fig. 17).

These results indicate that the promoting effect of SCCs stems from their ability to intercept the species promoting the formation of coke and that this likely proceeds via selective hydrogenation, as also reflected by the dependency of the CT enhancement factor on the H_2_ pressure. Investigations of the reactivity of CH_2_O and of mixed butadiene-butene feeds over the Pd/SiO_2_ SCC in the presence of H_2_ show that CH_2_O decomposes into CO and H_2_, with minor CH_4_ formation, whereas butadiene is selectively hydrogenated to butene (Supplementary Figs. 18–21). These findings reveal that different deactivating intermediates can follow distinct pathways, with polyenes and possibly other unsaturated species being responsible for the observed dependence on H_2_. Notably, in contrast to the sole zeolite systems in which high pressures are required to initiate the hydrogenation activity of BAS, the addition of SCC enables that moderate H_2_ pressures can effectively enhance the zeolite lifetime and the cumulative alkene productivity. Based on process needs, a threshold in the H_2_ partial pressure (within *p*_H2_ ≈ 0.1–1.0 bar) could be determined to balance productivity enhancement with H_2_ consumption.

### Induction period and Pd speciation

**Fig. 5:**
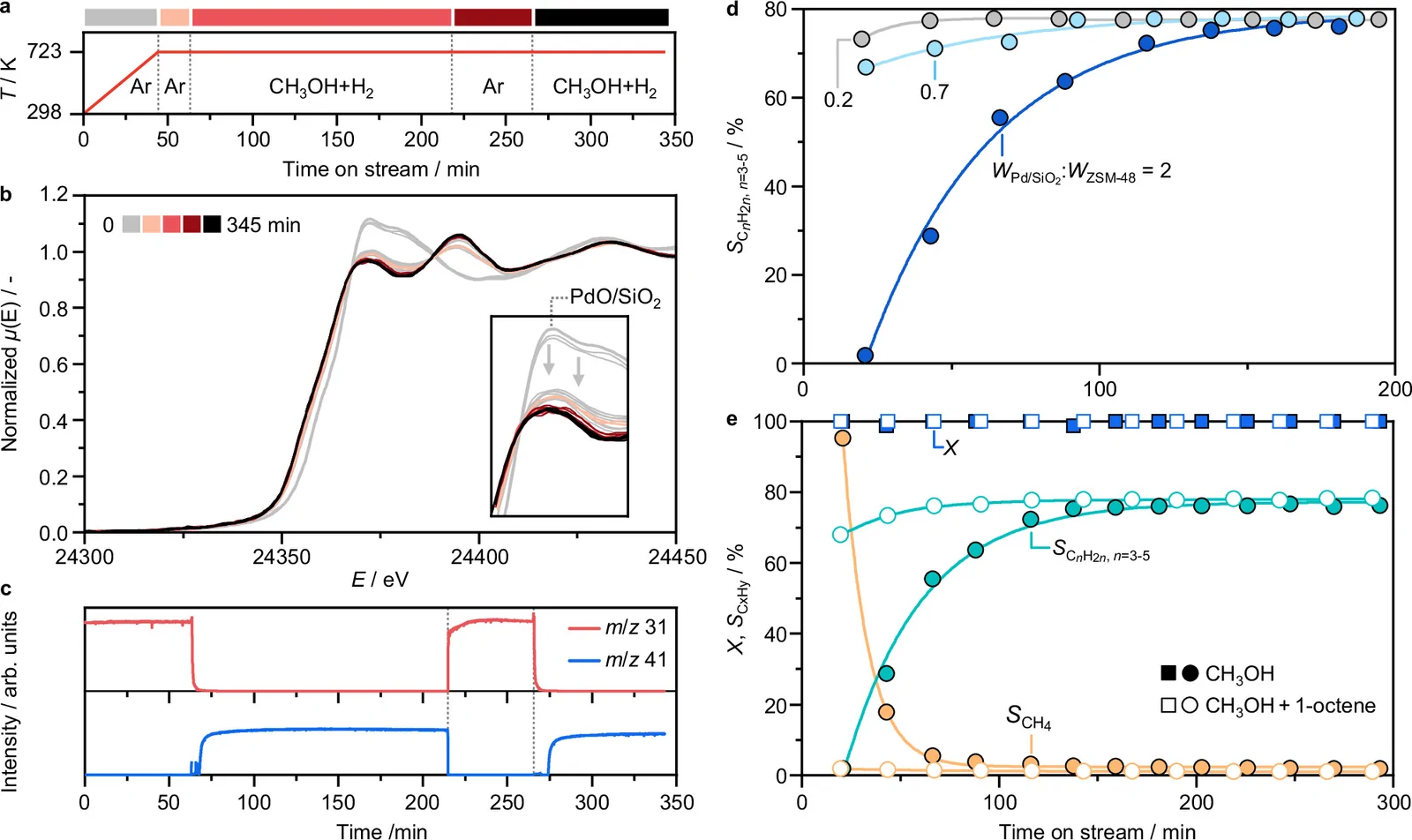
Investigation of the Pd speciation by operando XAS and control of the induction period. **a** Sequence of reactive environments to which the ZSM-48-Pd/SiO_2_ MCC catalyst is exposed during the operando XAS experiment, and **b** corresponding Pd K-edge XANES spectra. The fresh SCC contains PdO that undergoes a partial reduction even during temperature ramping in Ar, likely induced by the X-ray beam. Upon exposure to the H_2_-rich reactant feed, the reduction is completed, yielding metallic Pd that remains structurally unaltered during multiple MTH cycles. **c** Temporal evolution of mass fragments *m*/*z* 31 (CH_3_O^+^) and 41 (C_3_H_5_^+^). The time scale corresponds to the one shown in (**a**). Reaction conditions: WHSV = 0.7 g_CH3OH_ g_zeo_ h^–1^, *W*_Pd/SiO2_:*W*_zeolite_ = 0.5, *T* = 723 K, and *P* ≈ 1.0 bar. **d** Initial selectivity to C_3_–C_5_ alkenes (*S*_C*n*H2*n*, *n* = 3–5_) vs.  Pd/SiO_2_:zeolite weight ratio. **e** The impact of 1-octene co-feeding on the initial methane and C_3_–C_5_ alkene selectivity. Reaction conditions: CH_3_OH:C_8_H_18_:H_2_ = 10:0:90 (**a**), 9.9:0.1:90 mol% (**b**), WHSV = 4.8 g_CH3OH_ g_zeo_ h^–1^, *W*_PdSiO2_:*W*_zeolite_ = 2, *T* = 723 K, and *P* = 1.2 bar.

The initial induction period over the MCC in which alkene productivity rises to match the value of the parent zeolite (see Figs. 1a and 2a, and previous discussion) might suggest the reaction-induced modification of the starting Pd-phase. *Operando* Pd K-edge XAS indicates that PdO present in the calcined SCC is reduced to metallic Pd during temperature ramping in Ar and exposure to the H_2_ co-feed (Fig. 5). Consistent with the tests performed in a conventional microreactor, the temporal profile of alkene-specific fragment at *m*/*z* 41 exhibits a short induction period (Fig. 5c), which is also observable during a second MTH reaction that follows an intermediate Ar treatment (≈ 60 min). Notably, Pd(0) state remains unaltered during the reaction, as well as during cycles of reactive (CH_3_OH + H_2_) and inert atmosphere treatments (Fig. 5a, b), excluding the occurrence of deep structural changes of the Pd phase induced by reacting environment (Supplementary Fig. 22). The absence of detectable reaction-induced restructuring suggests that the modulation of Pd hydrogenation activity that enables the formation of alkenes with high selectivity, is likely originating from the surface-confined effects, such as competitive adsorption of strongly binding intermediates (e.g., polyenes) on Pd(0) and poisoning species that accumulate during MTH. These results are consistent with the suppression of alkene over-hydrogenation induced by oxygenates and dienes in the MTH mixture (vide infra, Supplementary Figs. 19–21). In line with this interpretation, the induction period is shortened upon decreasing the Pd/SiO_2_:zeolite weight ratio, i.e., at longer contact times of hydrocarbons per Pd site (see Fig. 5d). Additionally, if 1-octene (6.0 mol_C_%) is co-fed with methanol over ZSM-48-Pd/SiO_2_ MCC, thus promoting the autocatalytic MTH reaction and accumulation of its intermediates, the induction period is absent (Fig. 5e).

To gain insights into the influence of selected species over the hydrogenation activity of the Pd/SiO_2_ SCC, a series of experiments was performed feeding model mixtures of 1-butene, H_2_, and a co-reactant, chosen between CH_2_O or butadiene, in the absence of the zeolite catalyst (Supplementary Figs. 19–21). While pure streams of 1-butene in H_2_ undergo complete hydrogenation on Pd/SiO_2_ at 723 K, both oxygenates and butadiene were found to inhibit butene consumption. Furthermore, butadiene is selectively hydrogenated in the presence of butene. Despite simplifying the complex composition of the MTH product mixture, these experiments indicate that species formed during MTH can prevent over-hydrogenation over SCC, thus steering the hydrocarbon selectivity toward alkenes.

## Discussion

**Fig. 6:**
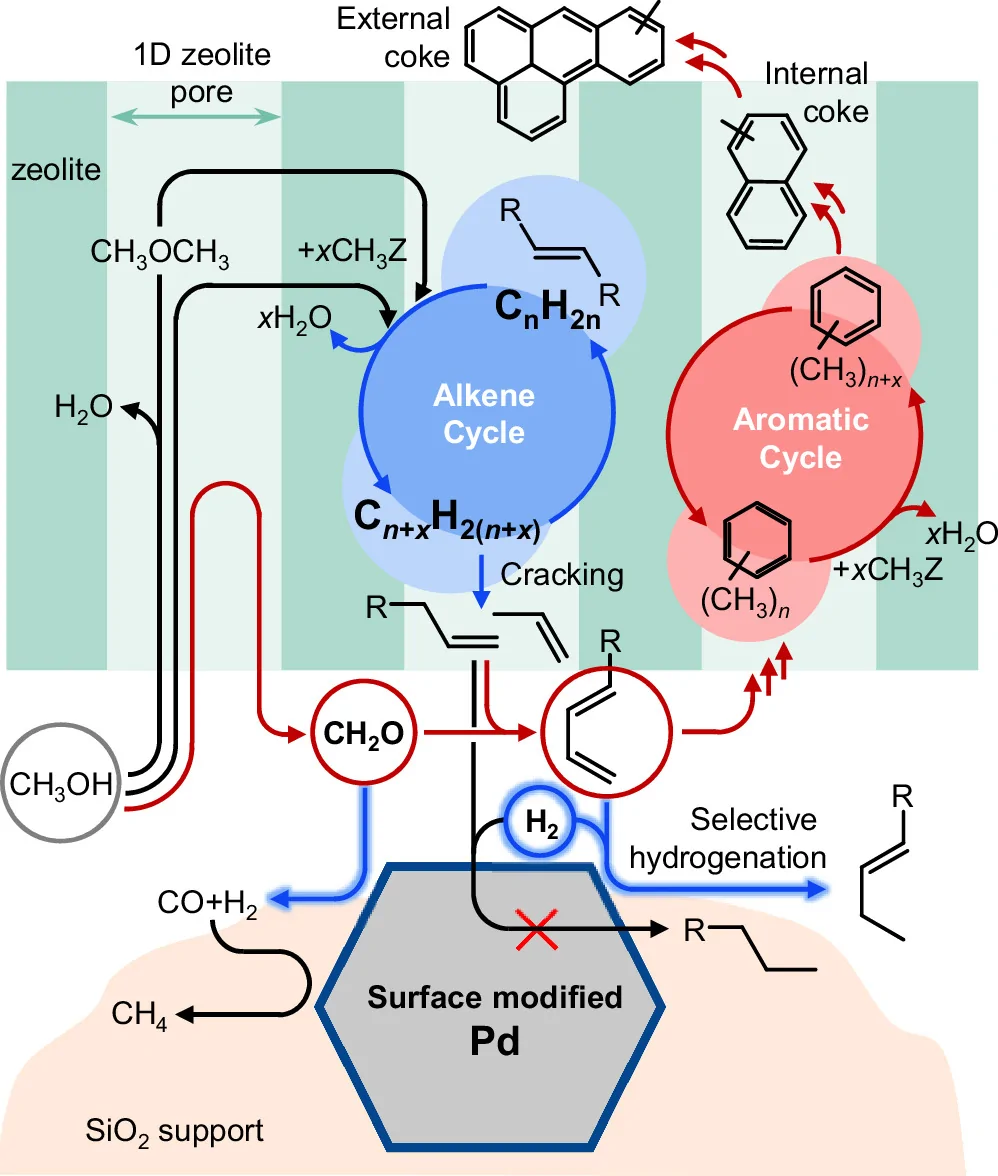
Proposed mechanism for the superior performance of MCC catalyst with H_2_ co-feeds in MTH. Under reaction conditions, Pd(0) nanoparticles selectively intercept coke precursors (blue arrows), either via hydrogenation (e.g., polyenes) or via decomposition (CH₂O). The complex reactant mixture modulates the hydrogenation activity of the Pd surface, preventing over-hydrogenation of alkene products. The removal of deactivating intermediates suppresses deactivation pathways (red arrows), thereby enhancing the cumulative productivity of C_3_–C_5_ alkenes.

This study introduces a novel approach to enhance the productivity of C_3_–C_5_ alkenes during MTH over 1D 10-MR zeolites by conveniently mixing the zeolite with an SCC, and using H_2_ as co-feed at near ambient pressure, thus not requiring direct modifications of the zeolite nor high pressures. Systematic investigation of different supported metals identified Pd/SiO_2_ as the best performing SCC system, achieving up to 10× better alkene productivity. The loading of SCC in the catalyst bed can be adjusted to minimize the use of Pd, as the productivity enhancement rapidly attains saturation at higher SCC loadings. Kinetic investigations indicate that the mechanism behind this effect stems from the selective interception of deactivating species present at the surface of the zeolite particles or gaseous intermediates evolving from them during MTH transformation, such as hydrogenation of polyenes, and decomposition of CH_2_O (Fig. 6). Consistently, *operando* DR-UV-vis experiments and TGA analyses of spent catalysts indicate reduced coke formation compared to the parent material. During an induction period, PdO is reduced to metallic Pd, which, under the reactant atmosphere, selectively favors alkene products as a result of surface reactivity modulation by oxygenates and polyunsaturated hydrocarbon species. Overall, these results advance a new concept to boost C_3+_ alkene productivity from potentially renewable feedstocks, mitigating zeolite coking thanks to the selective action of Pd/SiO_2_ and hydrogen. Given that SiO_2_ is a commonly used binder component in industrial catalyst formulations, these results hold great potential for scale-up into technical catalysts.

## Methods

### Catalyst preparation

A commercial ZSM-22 zeolite (ACS Material, nominal SiO_2_/Al_2_O_3_ = 65–80), in protonic form, was used as received from the supplier. A commercial ZSM-48 zeolite (Shandong Famous Materials, nominal SiO_2_/Al_2_O_3_ = 130) was received in ammonium form and was calcined in static air at 823 K (5 K min^−1^) for 5 h to yield the protonic form of the zeolite. The SCCs were prepared by incipient-wetness impregnation with a solution of the metal, targeting a metal loading of 67 µmol_M_ g_SiO2_^−1^. The following metal precursors were used: cobalt(II) nitrate hexahydrate (Sigma-Aldrich, >99.0%), silver nitrate (Puratrem, >99.999%), iron(III) acetylacetonate (Sigma-Aldrich, >99.9%), copper(II) acetylacetonate (Aldrich, >99.9%), ammonium tungstate (Johnson Matthey), Pd(II) nitrate (abcr GmbH, 99.9%). Silica (SiO_2_, Davisil Grade 635, Sigma-Aldrich) with BET surface area *S*_BET_ = 480 m^2^ g^−1^ and pore volume *V*_p_ = 0.75 cm^3 ^g^−1^ was used as support. All metal precursors were dissolved in water, except for the iron and copper precursors, which were dissolved in CHCl_3_ (Sigma-Aldrich, 99.8%). Pt/SiO_2_ and Ni/SiO_2_ were prepared by ion-adsorption, using as metal precursors tetraammineplatinum(II) nitrate (Sigma-Aldrich, >99.99%) and nickel(II) nitrate hexahydrate (Sigma-Aldrich, >97%). The required amount of metal nitrate was dissolved in water (33 cm^3^ per gram of SiO_2_), then SiO_2_ was added under vigorous stirring. The suspension was heated up to 343 K and left stirring for 2 h at this temperature. The solid was retrieved by filtration and washed with water. Following impregnation/ion-adsorption, the solids were dried under vacuum (50 mbar) at 343 K for 12 h, and calcined in static air at 823 K (5 K min^−1^) for 5 h to yield the SCCs. The bifunctional catalyst Pd/ZSM-22 was prepared by ion-exchange following an identical protocol, replacing SiO_2_ with ZSM-22 and using Pd(II) nitrate (abcr GmbH, 99.9%) as a metal precursor. The multicomponent catalysts (zeolite + SCC) were prepared by physical mixing. The parent zeolite and the SCC were each sieved to a fraction of 0.125–0.250 mm. Appropriate amounts of the sieved materials, necessary to attain a target zeolite:Pd/SiO_2_ weight ratio, were mixed. To study the influence of zeolite-SCC proximity in MCC on the reaction performance, ZSM-22 and Pd/SiO_2_ were grinded together in an agate mortar, and the powder thus obtained was then sieved.

### Catalyst characterization

Powder XRD measurements were performed on a PANalytical X’Pert PRO-MPD diffractometer using Cu Kα radiation (*λ* = 1.54060 Å) and a X’Celerator detector. Data were recorded in the 10–70°2*θ* range with an angular step size of 0.05° and a counting time of 1.5 s per step. N_2_ adsorption at 77 K was measured using a Micromeritics TriStar II analyzer. The samples were degassed at 473 K for 6 h prior to measurement. The *t*-plot method was used to estimate the micropore volume (*V*_micro_), and the total surface area (*S*_BET_) was calculated by the Brunauer-Emmett-Teller (BET) method. Temperature programmed desorption of ammonia (NH_3_-TPD) was performed in a homebuilt setup connected to an online quadrupole mass spectrometer (MS; Pfeiffer OmniStar GSD 320). The sample (0.100 g) was loaded in a quartz reactor, dried at 823 K for 30 min (heating rate 10 K min^–1^) under Ar (PanGas 5.0, *F*_Ar_ = 50 cm_STP_^3^ min^–1^), and then cooled to 393 K. Ammonia (PanGas, 10 mol% in He, *F*_NH3+He_ = 40 cm_STP_^3^ min^–1^) was adsorbed at this temperature for 30 min, followed by purging with Ar (*F*_Ar_ = 50 cm_STP_^3^ min^–1^) for 1 h to remove the loosely bound NH_3_. The remaining ammonia was desorbed by heating the sample to 873 K at a rate of 10 K min^–1^, while monitoring the signal of the molecular fragment *m*/*z* 17 using the MS. The concentration of medium and strong acid sites was determined upon deconvolution of the NH_3_**-**TPD profile (Supplementary Table 1 and Supplementary Fig. 6). Transmission electron microscopy (TEM), scanning TEM (STEM), and energy-dispersive X-ray (EDX) spectroscopy measurements were performed on a JEOL JEM F200. Samples were prepared by suspending the ground material in ethanol, followed by dispersion via ultrasonication. Approximately 2 μL of the suspension was drop-cast onto a holey carbon-coated copper grid, which was plasma-cleaned in a JEOL EC 52000IC ion cleaner for 5 min at about 300 V before being loaded into the microscope on a single tilt holder. The samples were measured in a dynamic vacuum in the range of 10^−7 ^mbar. The cold-field emission gun of the microscope was operated at an accelerating voltage of 200 kV, and the emission current was set to 12–15 μA. TEM micrographs were acquired with a 4096 × 4096 Gatan Rio16 complementary metal-oxide-semiconductor (CMOS) camera with an exposure time of 0.25–0.6 s and a frame rate of 20 frames/s. Bright field (BF) and dark field (DF) STEM images with a resolution of 1024 × 1024 were recorded with a dwell time of 16 μs/pixel. A total of 512 × 512 EDX maps were collected for about 10 min with a dwell time of 0.1 ms/pixel. TEM and STEM images were analyzed using Gatan Digital Micrograph, while EDX maps were evaluated with the JEOL Analysis Station software. The coke content in deactivated catalysts was determined by thermogravimetric analysis using a NETZSCH STA 449 Jupiter analyzer. The sample (*ca*. 0.035 g) was first dried at 373 K for 30 min, then heated to 1073 K under a flow of air (*F*_air_ = 100 cm_STP_^3^ min^−1^) with a heating rate of 10 K min^–1^, and finally held at this temperature for 20 min to ensure complete coke combustion.

### Catalyst testing

The sieved catalysts (0.125–0.250 mm) were mixed with quartz sand as diluent (0.350–0.500 mm, Brenntag Schweizerhall AG) using a (*W*_Pd/SiO2_ + *W*_sand_):*W*_zeolite_ weight ratio of 3 for ZSM-22 and 6 for ZSM-48, and loaded into a tubular quartz reactor for catalytic testing (zeolite weight *W*_zeolite_ = 0.100 g or 0.200 g for ZSM-48 and ZSM-22, respectively). All MTH reactions were carried out in an automated home-built continuous-flow fixed-bed reactor setup described previously^32^. The catalyst was preactivated in situ in oxygen flow (PanGas, purity 5.0, 100 cm^3^_STP_ min^−1^) at 823 K for 10 min, and then brought to 723 K under Ar (PanGas, purity 5.0, 225 cm^3^_STP_ min^–1^). Methanol (Sigma-Aldrich, HPLC grade, >99.9%) was fed with a syringe pump to an evaporator at a liquid flow rate of 0.01 cm_liq_^3^ min^−1^, corresponding to a gas flow of *F*_CH3OH_ = 5.5 cm^3^_STP_ min^−1^, and diluted with a flow of H_2_ (PanGas, purity 5.0) and/or Ar (*F*_H2+Ar_ = 51 cm^3^_STP_ min^−1^, *c*_CH3OH_ ≈ 10 mol%). The reactant feed was stabilized through a bypass line and then introduced into the packed reactor at a total pressure of 1.2 bar. Co-feeding experiments were performed by feeding formaldehyde (Sigma-Aldrich, 37 wt% solution in water), 3-buten-1-ol (Aldrich, >98%), or 1-octene (Acros organics, >99%) dissolved in methanol in the required concentration (within 2–6 mol_C_ %). Details on product analytics, conversion, and selectivity calculation are available in the Supplementary Information.

### Operando DR-UV-vis

DR-UV-vis measurements were performed using a home-built setup comprising a quartz flow cell mounted in a customized oven, an Ocean Optics DH-2000-BAL deuterium/halogen light source, and an Ocean Optics Maya 2000-Pro UV-vis spectrometer equipped with a reflection probe (Ocean Optics six-around-one probe). The quartz cell was loaded with the MCC mixture (*W*_zeolite_ = 0.100 g and 0.150 g for ZSM-48 and ZSM-22, respectively; *W*_Pd/SiO2_:*W*_zeolite_ = 0.2) and brought to 723 K under Ar (245 cm^3^_STP_ min^−1^), monitoring the temperature with a K-type thermocouple positioned in the catalyst bed. The methanol feed was produced by passing a stream of Ar or H_2_ (27 and 33 cm^3^_STP_ min^−1^ for ZSM-22 and ZSM-48, respectively) through a bubbler filled with liquid CH_3_OH and maintained at a temperature of 298 K (*c*_CH3OH_ ≈ 17 mol%). The CH_3_OH feed was stabilized through a bypass line, then introduced through the UV-vis cell at 1.0 bar. For the hexene cracking experiment, the quartz cell was loaded with ZSM-22 (*W*_zeolite_ = 0.150 g) and the reactant feed was produced by passing a stream of Ar (27 cm^3^_STP_ min^−1^) through a bubbler filled with liquid 1-hexene maintained at a temperature of 298 K (*c*_C6H12_ ≈ 25 mol%). Spectra were acquired over 45 min (2 min for hexene cracking) within the SpectraSuite package using an integration time of approximately 250 ms and collecting 5 scans to average for each spectrum. The raw spectra were smoothed in Origin(Pro) 2024, applying a Savitzky-Golay filter.

### Operando XAS

Operando XAS measurements at the Pd K-edge were carried out at the Debye beamline of the Swiss Light Source 2.0 (SLS). operating at 400 mA ring current. The X-ray beam produced from the 2.1 T bending magnet source was collimated with a Pt-coated mirror at 2.64 mrad. Subsequently, the beam was monochromatized with a liquid N_2_-cooled Si (311) channel cut crystal. The monochromatic X-ray beam was focused with a Pt-coated toroidal double focusing mirror with a beam size of 1.0 mm (horizontal) × 0.2 mm (vertical) on the sample. Gas-filled ionization chambers (1.9 bar, N_2_:Ar = 25:75 mol%) were used to measure incident and transmitted beam intensity. Simultaneously, a Pd reference foil was measured for energy calibration. ZSM-48-Pd/SiO_2_ MCC (15 mg, *W*_Pd/SiO2_:*W*_zeolite_ = 0.5) was loaded in a borosilicate glass capillary (OD = 2.0 mm, wall thickness 0.2 mm) between two plugs of quartz wool. The capillary was brought to 473 K in Ar (14 cm^3^_STP_ min^−1^), with a heating rate of 10 K min^−1^. The CH_3_OH feed was produced by passing a stream of H_2_ (2.7 cm^3^_STP_ min^−1^) through a bubbler filled with liquid CH_3_OH and maintained at a temperature of 273 K (*c*_CH3OH_ ≈ 4 mol%). The CH_3_OH feed was stabilized through a bypass line, then introduced through the packed capillary at ≈ 1.0 bar. Two MTH cycles were performed, separated by an Ar purge. The composition of the reactor inlet and outlet was analyzed by online MS. XAS spectra were acquired during the whole process (5 s per spectrum) and averaged over 5 min. Spectra were recorded in fluorescence mode for the sample using a PIPS detector at room temperature^47^. The resulting raw data and EXAFS spectra were analyzed by using the Demeter software package^48^.

## Supplementary information

Supplementary Information

Transparent Peer Review file

## Data Availability

All data generated or analyzed during this study are included in this published article (and its Supplementary Information file), or can be obtained from the authors upon request.
