# Supplementary material for: Enhanced alkene productivity in methanol-to-hydrocarbons conversion using a secondary catalyst component and hydrogen co-feeds
Source: Nat Commun. 2026 Sep 23;17:11065. doi: 10.1038/s41467-026-77944-9 (PMC13691128; doi:10.1038/s41467-026-77944-9)
Supplement: Supplementary file 1 [file 41467_2026_77944_MOESM1_ESM.pdf]

Supplementary Information

Enhanced alkene productivity in methanol-to-hydrocarbons conversion using a secondary catalyst component and hydrogen co-feeds

Matteo Vanni,[1] *Andreas Brenig,*[2] *Adam H. Clark,*[3] *Jeroen A. van Bokhoven,*[1,2] *Vladimir Paunović*[1]*

[1]Center for Energy and Environmental Sciences, PSI, Forschungsstrasse 111, 5232 Villigen, Switzerland.

[2]Institute for Chemical and Bioengineering, Department of Chemistry and Applied Biosciences, ETH Zurich, Vladimir-Prelog-Weg 1, 8093 Zurich, Switzerland

[3]Center for Photon Science, PSI, Forschungsstrasse 111, 5232 Villigen, Switzerland.

Email:

vladimir.paunovic@psi.ch

Table of contents

Supplementary methods 3

Supplementary tables 6

Supplementary figures 7

Supplementary references 33

Supplementary methods

1. Reaction analytics in catalytic tests

The concentrations of methanol (CH_3OH), dimethyl ether (DME), and hydrocarbon products were analyzed using an online gas chromatograph (Agilent GC 6890) equipped with an HP PLOT-Q column and a flame ionization detector (FID). Both CH_3OH and dimethyl-ether (DME) were treated as reagent species in calculating conversion. The conversion (X), selectivity to hydrocarbon products C_xH_y (S_{CxHy}), cumulative selectivity to C_xH_y (CS_{CxHy}), cumulative productivity of C_3-C_5 alkenes ($CP_{C_nH_{2n},\ n=3\text{-}5}$), and methanol cumulative turnover (CT) were calculated according to **Equations 1-5**:

$$X = \frac{n_{CH_3OH}^{in} - n_{CH_3OH}^{out} - 2n_{DME}^{out}}{n_{CH_3OH}^{in}} \times 100,\ \% \qquad \textbf{1}$$

$$S_{C_xH_y} = \frac{x n_{C_xH_y}^{out}}{\sum_1^p x n_{C_xH_y}^{out}} \times 100,\ \% \qquad \textbf{2}$$

$$CS_{C_xH_y} = \frac{\int_0^t x n_{C_xH_y}^{out}\, dt}{\int_0^t \sum_1^p x n_{C_xH_y}^{out}\, dt} \times 100 \approx \frac{\int_0^t X S_{C_xH_y}\, dt}{\int_0^t X dt} \times 100,\ \% \qquad \textbf{3}$$

$$CP_{C_nH_{2n},\ n=3-5} = \frac{\int_0^t \sum_3^5 x n_{C_x}^{out}\, dt}{W_{zeolite}},\ mol_C\ g_{zeo}^{-1} \qquad \textbf{4}$$

$$CT = WHSV \int_0^t X dt,\ g_{CH_3OH}\ g_{zeo}^{-1} \qquad \textbf{5}$$

where $n_{j,in}$ and $n_{j,out}$ denote the molar flow of compound j at reactor inlet and outlet, C_xH_y denote hydrocarbons with x carbons, p is the total number of detectable products, and WHSV is the

weight hourly space velocity. The methanol cumulative turnover capacity from initial conversion up to a specific conversion X is denoted as CT_X. The methanol cumulative turnover capacity up to full deactivation (CT_0) was calculated using a nonlinear regression of X vs. time-on-stream data presented in this work (**Fig. 2a**) or calculated from the cited references (**Supplementary Fig. 9**), using Janssens kinetic model[1].

2. Kinetic investigation of the intrinsic reactivity of Pd/SiO$_2$ SCC

To better understand the fate of individual intermediates in the presence of the Pd/SiO_2 SCC and H_2 co-feeds, and to elucidate how alkene selectivity is preserved under strongly hydrogenating conditions, a series of experiments on model reacting systems was performed (**Supplementary Fig. 9, 18-21**). All reactions were carried out using the homemade continuous-flow fixed-bed reactor setup described in the main text, additionally equipped with a quadrupole mass spectrometer (MS; Pfeiffer OmniStar GSD 320). The MS was used for online sampling of either the reactor outlet or the inlet via a bypass line.

3. Supplementary notes on data regression in Fig. 4a

Based on previous studies on the origin of the stabilization effect provided by high-pressure H_2 co-feeds, and the data discussed in the main text, the alkene productivity enhancement is ascribed to the elimination of deactivating species by selective hydrogenation over Pd. In the first approximation, unsaturated deactivating species (e.g., polyenes) can be lumped into one single deactivating species DS, and the rate of hydrogenation over Pd can be considered to follow the **Equation 6**:

$$r_{H_2} = k_{H_2} c(\mathrm{DS}) p_{H_2}^{n_{H2}} \quad \mathbf{6}$$

where k_{H2} is a proportionality constant, c(DS) the concentration of deactivating species, p_{H2} the hydrogen partial pressure, and n_{H2} the reaction order with respect to H_2.

Assuming that c(DS) is constant within the methanol conversion range $X = 100\text{-}90\%$ used for the analysis, and that the cumulative productivity of C_3-C_5 alkenes ($\mathrm{CP}_{C_nH_{2n},\, n=3\text{-}5}$), resulting from the extended cumulative turnover capacity, is proportional to the rate of removal of deactivating species, **Equation 7** is obtained:

$$\mathrm{CP}_{C_n\mathrm{H}_{2n+2},\, n=3-5} = k' r_{\mathrm{H}_2} = k'' p_{\mathrm{H}_2}^{n_{\mathrm{H}_2}} \qquad \mathbf{7}$$

where k' and k" are proportionality constants. **Equation 7** is linearized in the log-log plot of **Fig. 4a** to determine n_{H2}.

Supplementary tables

Supplementary Table 1. Textural and acidic properties of the zeolite catalysts.

Zeolite	S_{BET}[b] / $m^2\ g^{-1}$	S_{ext}[c] / $m^2\ g^{-1}$	V_{micro}[c] / $cm^3\ g^{-1}$	V_{total}[e] / $cm^3\ g^{-1}$	$c_{medium\ sites}$[e] / $\mu mol\ g^{-1}$	$c_{strong\ sites}$[e] / $\mu mol\ g^{-1}$
ZSM-22	245	74	0.09	0.44	38	88
Pd/ZSM-22[a]	232	75	0.08	0.47	34	68
ZSM-48	221	93	0.06	0.24	74	172

[a] Measured after H_2 treatment at 723 K. [b] N_2 adsorption + BET model. [c] N_2 adsorption + *t*-plot model. [d] N_2 adsorption at $p/p_0 = 0.98$. [e] NH_3-TPD.

Supplementary Figures

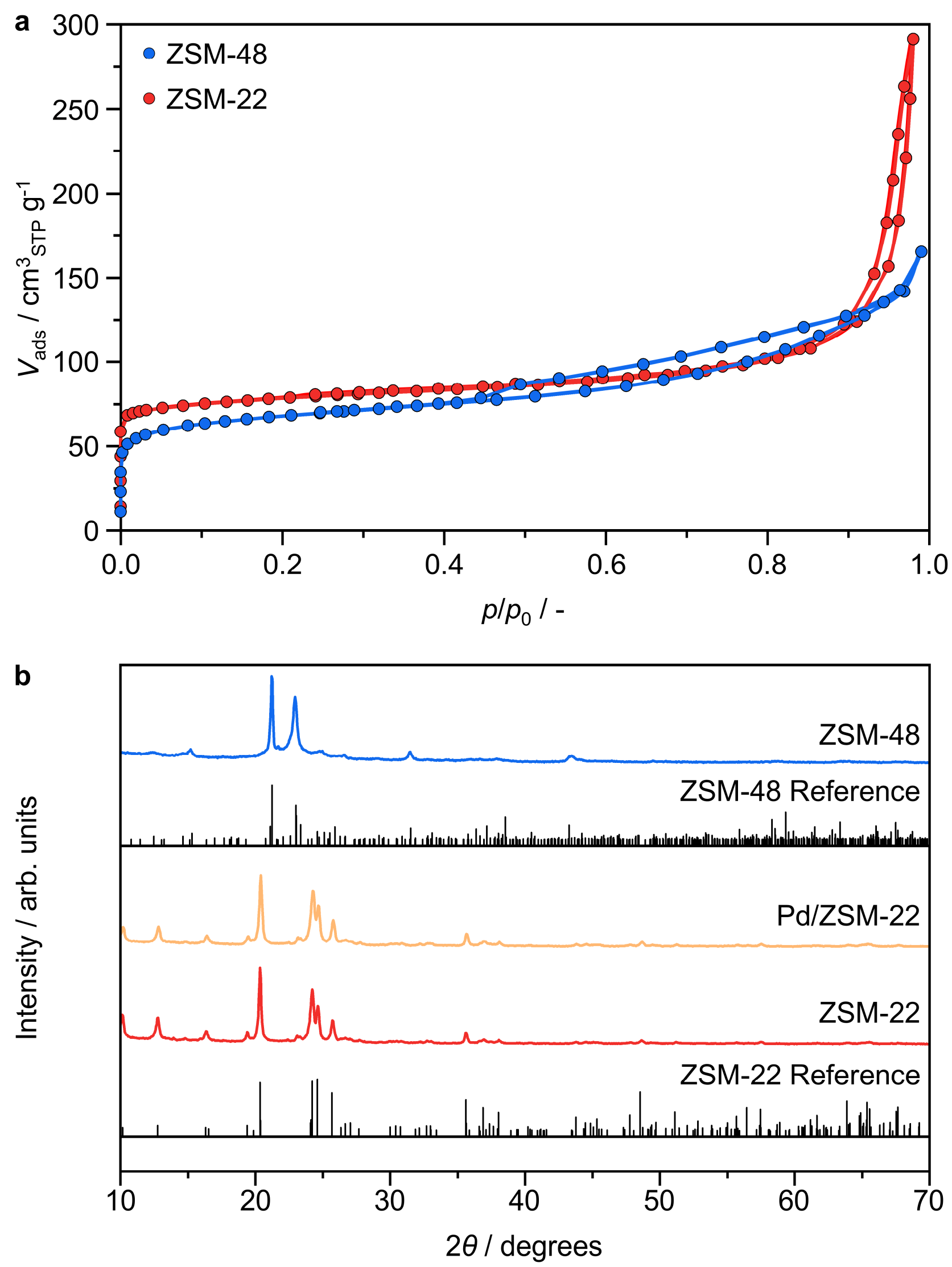

Supplementary Fig. 1. Textura and structural characterization of the zeolite catalysts. a N_2 adsorption isotherms at 77 K of the parent ZSM-22 and ZSM-48 zeolites, and **b** XRD profiles of the parent ZSM-22, Pd/ZSM-22, and parent ZSM-48 zeolites, together with reference patterns obtained from International Zeolite Association[2].

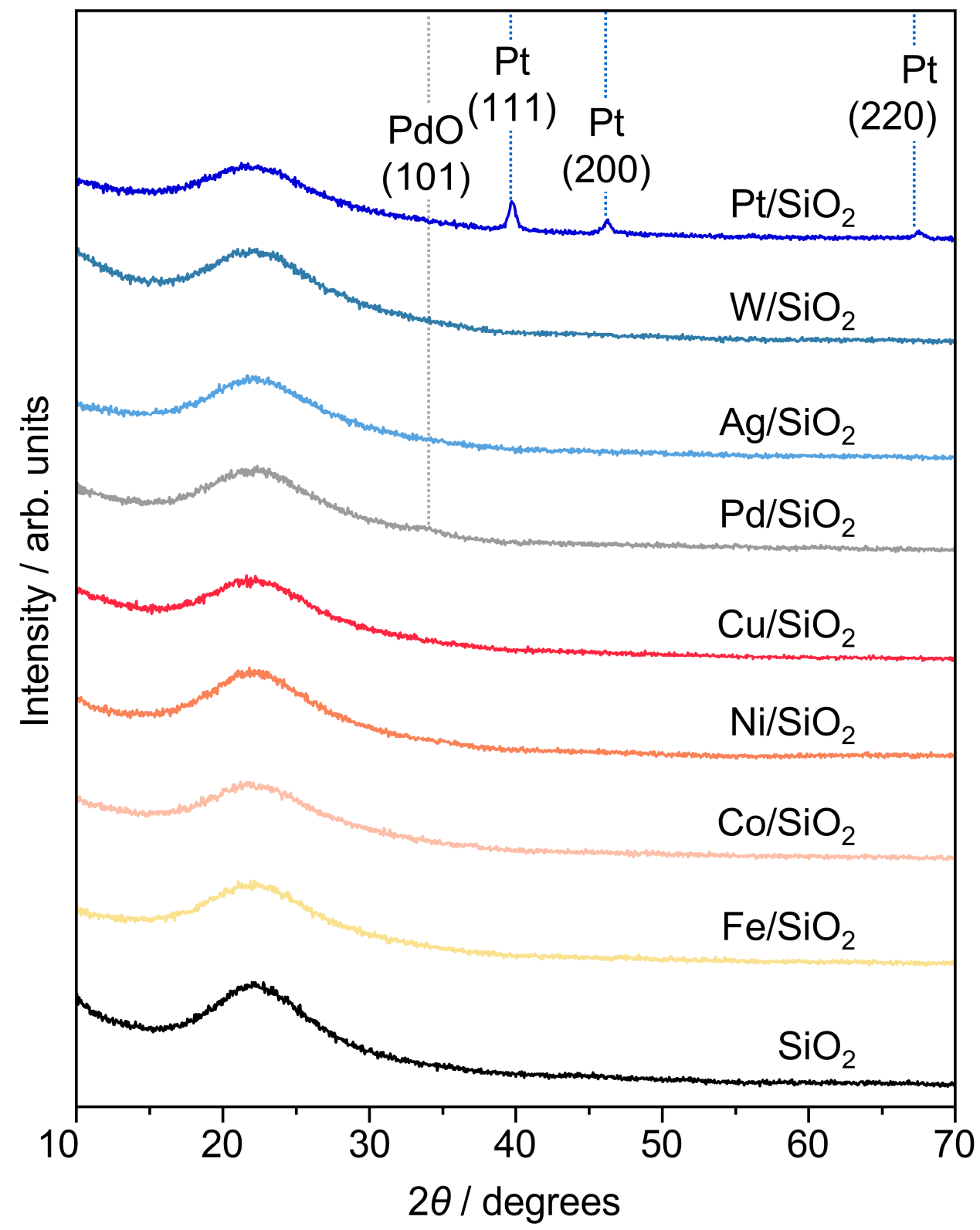

Supplementary Fig. 2. Powder XRD profiles of the calcined SCCs. A low-intensity, broad peak at $2\theta \approx 34°$, corresponding to the (101) reflection of PdO ($P4_2/mmc$), can be distinguished in Pd/SiO_2. Contrarily, sharp reflections are visible in Pt/SiO_2 at 39.7° and 46.2°, corresponding to larger metallic Pt (Fm-3m) aggregates with particle size $d_{Pt} \approx 15$ nm based on the Scherrer equation. The profiles of the other materials do not show peaks beyond the SiO_2 component, indicating a high metal dispersion.

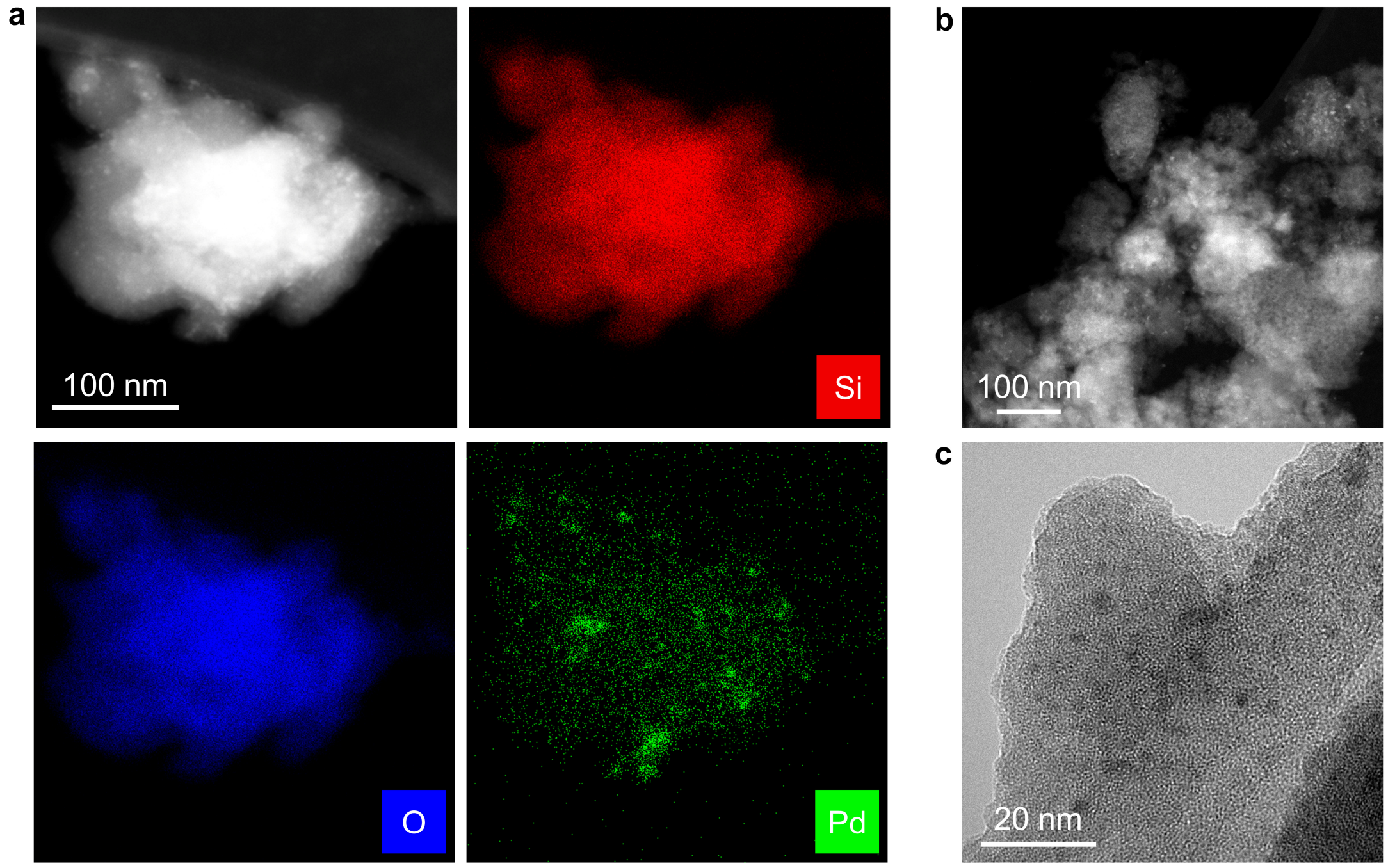

Supplementary Fig. 3. Electron microscopy of the fresh Pd/SiO_2 SCC. a STEM-EDX elemental mapping corresponding to the micrograph shown in (**Fig. 1c**), **b** additional STEM and **c** TEM characterization of the material. The EDX mapping at lower magnification indicates the mostly uniform distribution of Pd, the concentration of which may be higher in some regions. TEM imaging at higher magnification reveals Pd nanoparticles with average size 4.5 ± 1.8 nm.

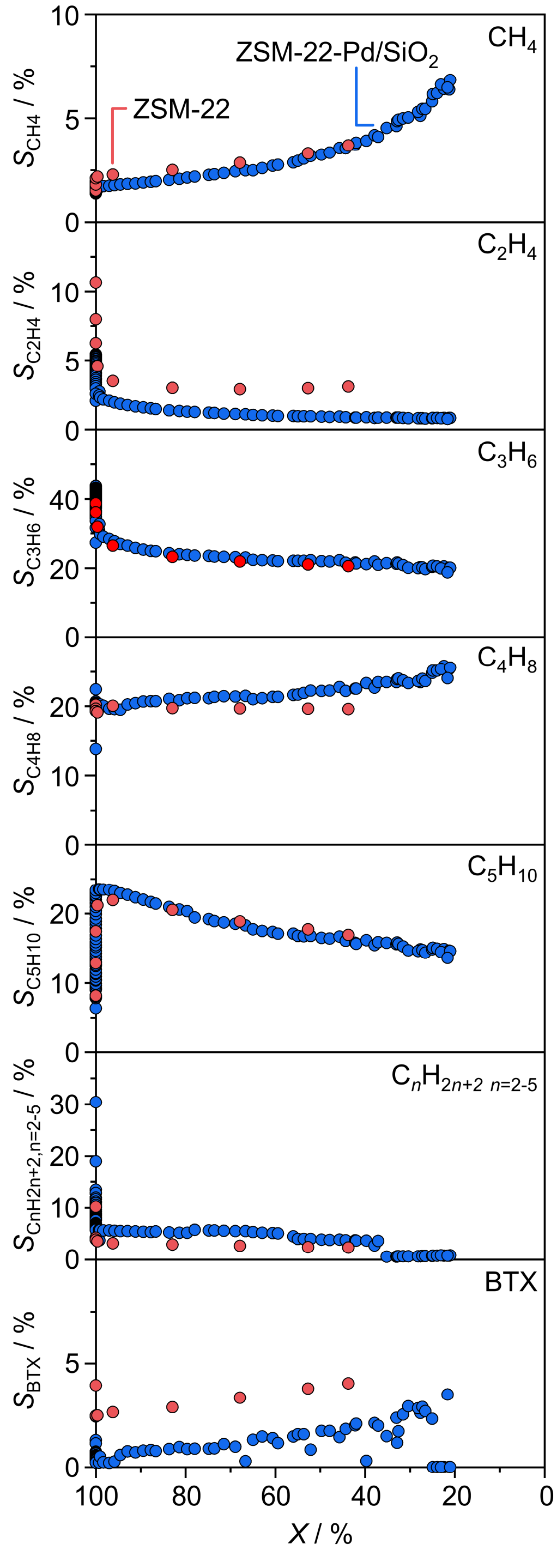

Supplementary Fig. 4. **Selectivity profile of ZSM-22-Pd/SiO$_2$ MCC.** Selectivity to specific hydrocarbons (S_{CxHy}) vs. conversion (X) profiles in MTH reaction with H_2 co-feed over the parent ZSM-22 and ZSM-22-Pd/SiO$_2$ MCC for selected hydrocarbon products. The selectivity profile over the MCC system follows that of the parent zeolite, while showing a diminished contribution of aromatic-cycle reactions (lower S_{C2H4} and S_{BTX}), and some over-hydrogenation

of alkene products, particularly at the reaction onset (higher $S_{CnH2n+2,n = 2-5}$). Reaction conditions are detailed in **Fig. 1** of the main manuscript.

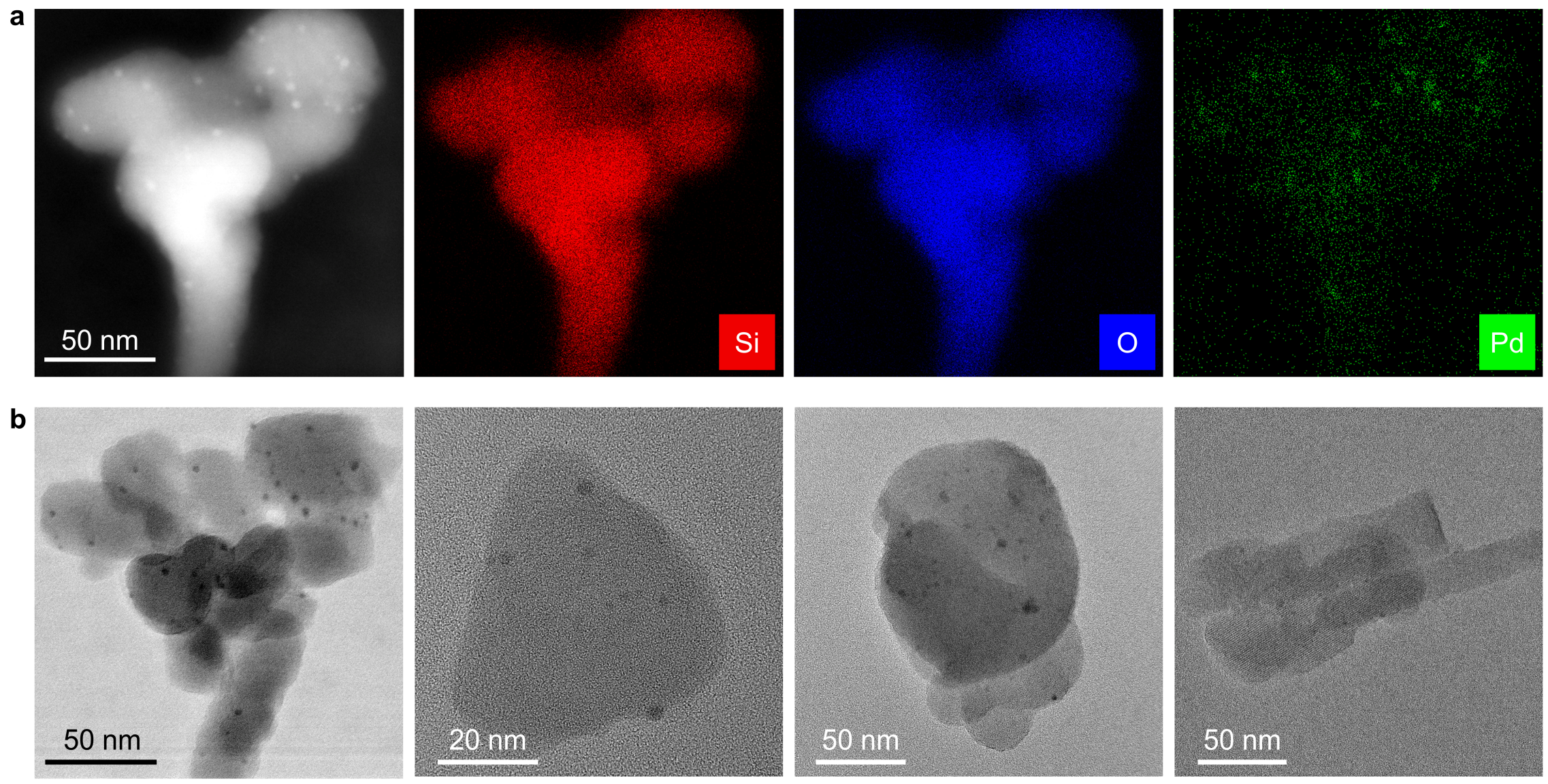

Supplementary Fig. 5. Electron microscopy of the fresh Pd/ZSM-22 bifunctional catalyst. **a** STEM-EDX elemental mapping, **b** additional STEM and TEM characterization of the material. Pd is well dispersed, with particle size 2.8 ± 0.6 nm, which is slightly smaller than in Pd/SiO_2.

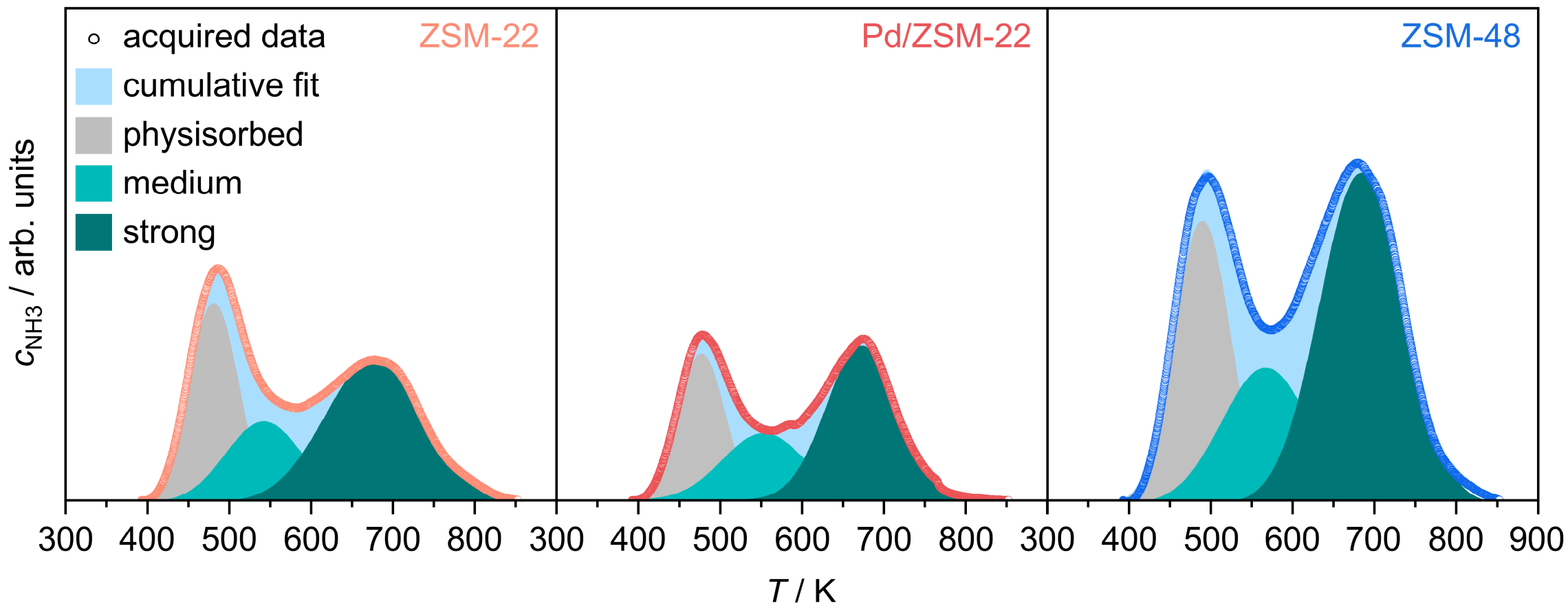

Supplementary Fig. 6. Acidity of the zeolite catalysts. NH_3-TPD profiles of ZSM-22, Pd/ZSM-22, and ZSM-48 catalysts in their fresh form. Pd/ZSM-22 was treated in H_2 at 723 K before NH_3 TPD measurement. Signals were deconvoluted into physisorbed and chemisorbed NH_3 contributions, the latter comprising weakly and strongly bound NH_3. The contributions from chemisorbed NH_3 were used to calculate the acidity of the catalysts reported in **Supplementary Table 1**.

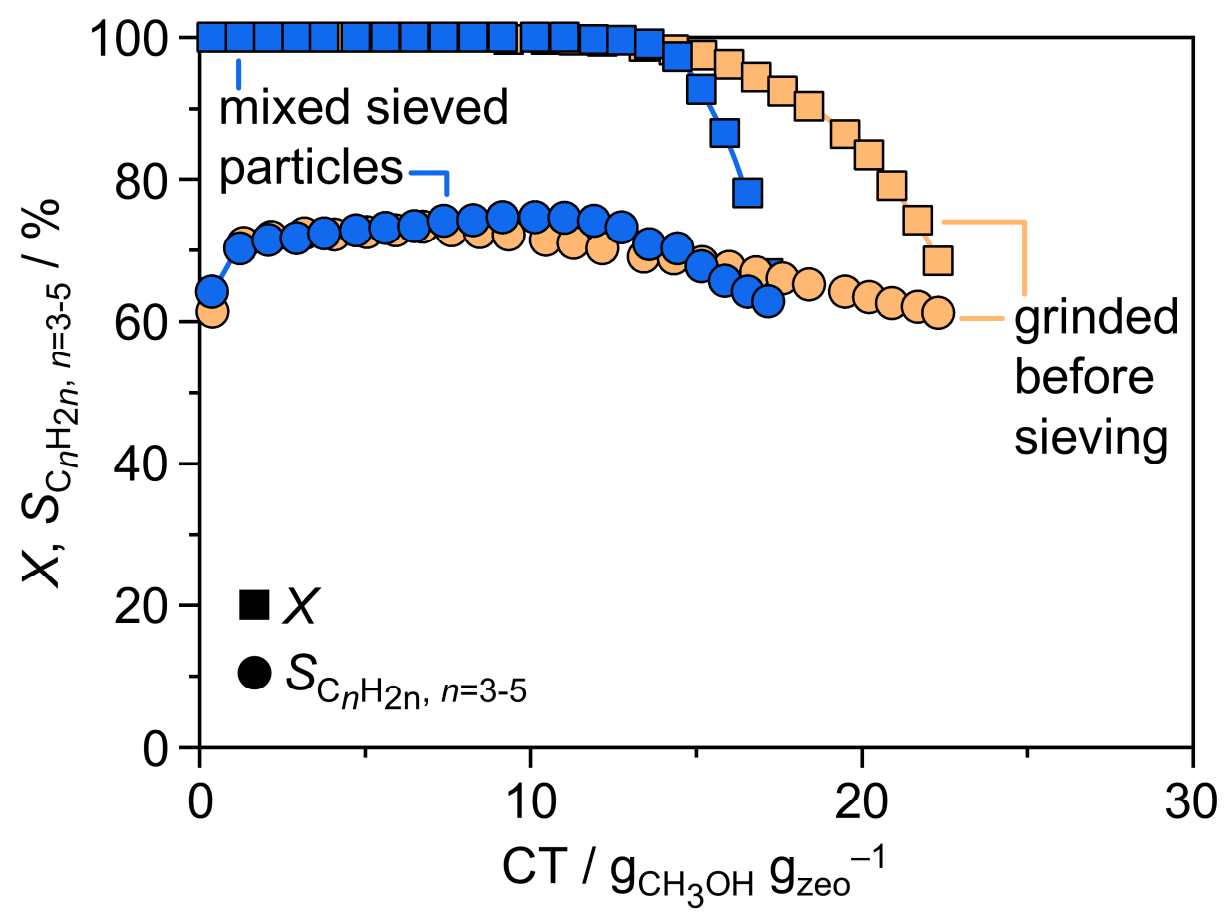

Supplementary Fig. 7. Impact of the proximity between Pd and zeolite active sites on the catalytic performance of the ZSM-22-Pd/SiO_2 MCC. Methanol conversion (X) and selectivity to C_3-C_5 alkenes ($S_{C_nH_{2n},\ n=3\text{-}5}$) vs. methanol cumulative turnover (CT) of ZSM-22-Pd/SiO_2 MCC, prepared by direct mixing of the sieved MCC components, or by grinding followed by sieving. No significant difference is observed, apart from a more gradual loss of activity at $X < 100\%$ for the ground system. The possibility to directly mix the zeolite and the SCC as powders opens up to the future development of shaped MCC catalysts. Reaction conditions: CH_3OH:Ar:H_2 = 10:80:10 mol%, WHSV = 2.4 $g_{CH3OH}\ g_{zeo}\ h^{-1}$, $W_{Pd/SiO2}$:$W_{zeolite}$ = 1, T = 723 K, and P = 1.2 bar.

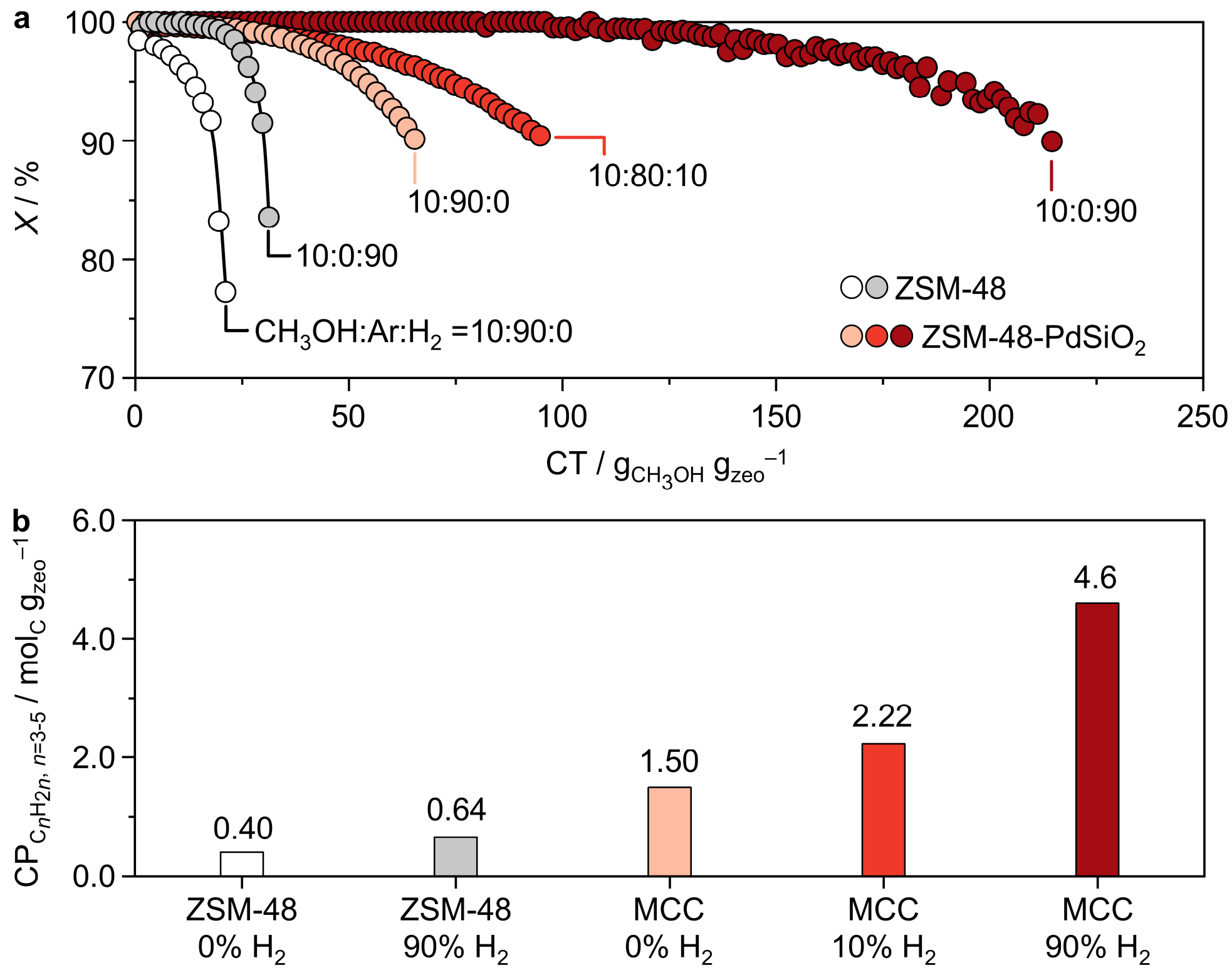

Supplementary Fig. 8. Impact of the use of Pd/SiO_2 SCC and H_2/Ar co-feed on the MTH performance of ZSM-48. a Methanol conversion (*X*) vs. methanol cumulative turnover (CT) and **b** corresponding cumulative productivity of C_3-C_5 alkenes ($CP_{C_nH_{2n},\ n=3\text{-}5}$) in the conversion range *X* = 100-90% for the parent ZSM-48 and the ZSM-48-Pd/SiO_2 MCC ($W_{Pd/SiO2}$:$W_{zeolite}$ = 2), at different concentrations of H_2 co-feed (0-90 mol%). The use of H_2 slightly increases the $CP_{C_nH_{2n},\ n=3\text{-}5}$ of the parent zeolite (1.6×), as well as the addition of Pd/SiO_2 SCC without H_2 (≈ 4×). The latter is ascribed to minor *in situ* formation of H_2 from CH_3OH and the interception of CH_2O on Pd by direct decomposition (**Supplementary Fig. 18**). Nonetheless, H_2 co-feeds are needed to maximize the effect of the MCC (≈ 12×), which is attributed to the more efficient removal of deactivating polyene intermediates by selective hydrogenation. Reaction conditions: CH_3OH:H_2:Ar=10:(90-0):(0-90), WHSV = 4.8 $g_{CH3OH}\ g_{zeo}\ h^{-1}$, *T* = 723K, and *P* = 1.3 bar.

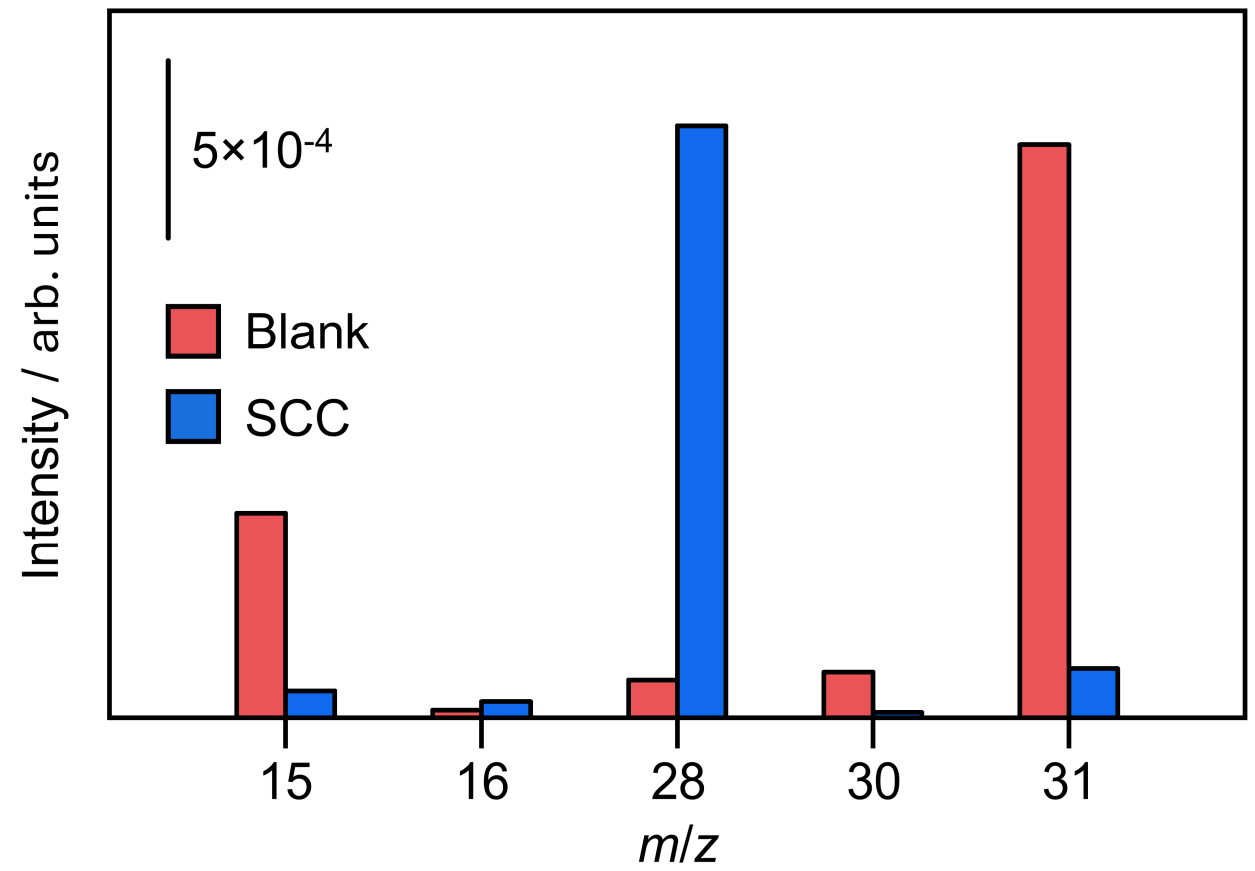

Supplementary Fig. 9. Reactivity of CH_3OH over Pd/SiO_2 SCC with H_2 co-feed. MS fragmentation pattern from the reaction of CH_3OH in H_2 over Pd/SiO_2 SCC, and comparison with the corresponding blank. A mixture of CH_3OH and H_2O diluted in H_2 (F_{CH3OH} = 6 cm^3_{STP} min^{-1}, F_T = 57 cm^3_{STP} min^{-1}, $c_{CH3OH} \approx$ 10 mol%) was fed to the reactor packed with Pd/SiO_2 SCC mixed with quartz sand (W_{SCC} = 50 mg, W_{sand} = 600 mg). The fragmentation measured pattern was compared with a blank. The drop in *m*/*z* 31 with respect to the blank indicates CH_3OH consumption, with the marked rise in *m*/*z* 28 pointing to decomposition into CO and H_2. To a less extent, some methanation occurs, as indicated by a small rise in *m/z* 16. Reaction conditions: CH_3OH:H_2O:H_2 = 10.1:1.8:88.1 mol%, WHSV = 9.9 g_{CH3OH} $g_{Pd/SiO2}$ h^{-1}, T = 723 K, and P = 1.1 bar. The signal is averaged over 8 minutes.

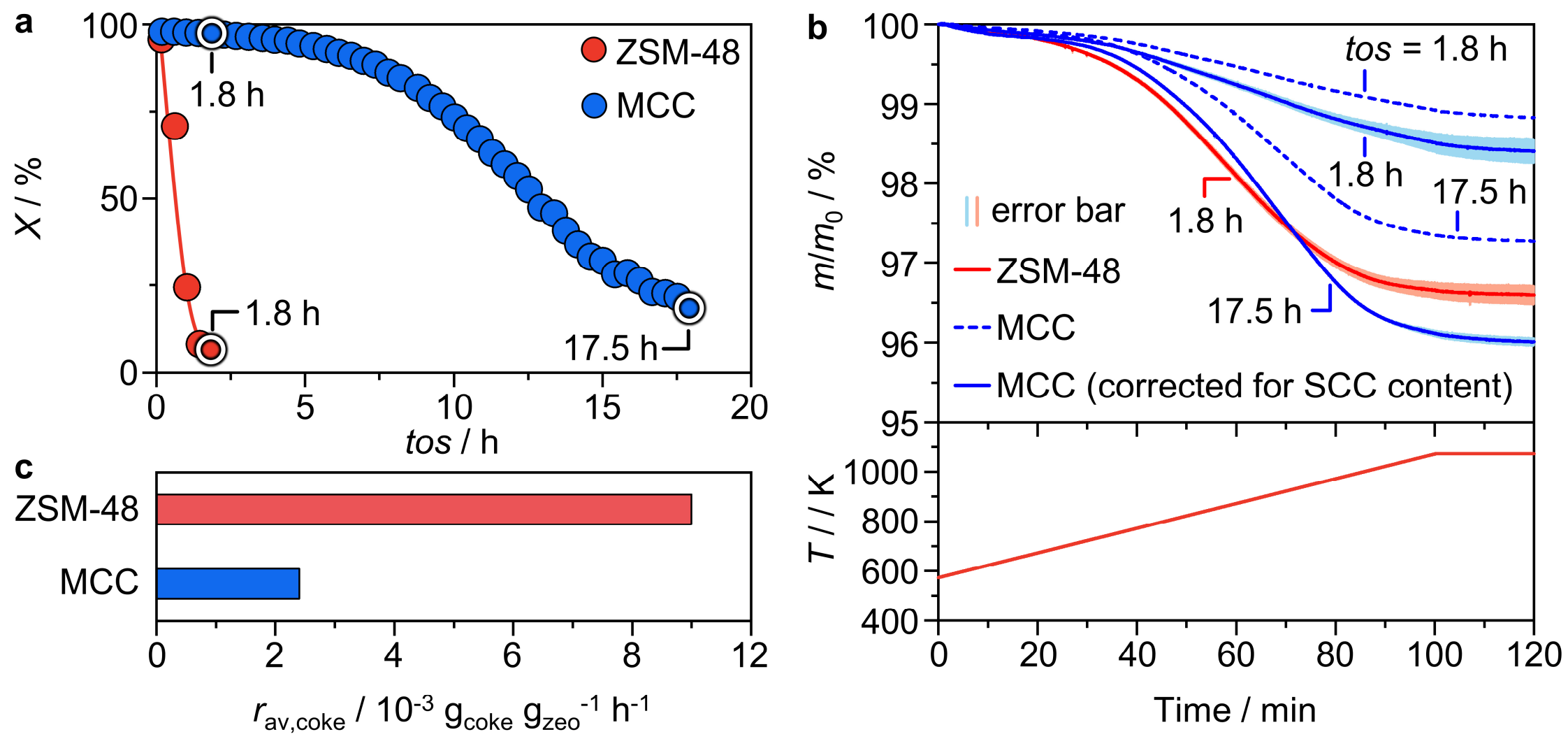

Supplementary Fig. 10. Quantitative assessment of coke deposition in ZSM-48 and ZSM-48-Pd/SiO_2 MCC. a Methanol conversion (*X*) vs. time-on-stream (*tos*) profiles under accelerated deactivation conditions (c_{CH3OH} = 19 mol%, WHSV = 11.8 g_{CH3OH} g_{zeo} h^{-1}). The points at which partially deactivated catalysts were retrieved for thermogravimetric analysis (TGA) are highlighted. Notably, a significant lifetime enhancement over the MCC system with H_2 co-feeding is observed even at elevated CH_3OH concentrations. Data are shown as the mean of two independent measurements; error bars indicate the range of the two replicate values. Reaction conditions: CH_3OH:H_2=19.6:80.4, W_{PdSiO2}:$W_{zeolite}$ = 0.5, T = 723K, and P = 1.3 bar. **b** Corresponding TGA profiles. Error bars are calculated from two replicates of each measurement. To enable a direct comparison with the parent ZSM-48, the MCC profiles are corrected for the content of SCC (uncorrected measurements are also displayed). **c** Average rate of coke formation over the time span shown in (**a**). At a deactivation level of X = 12 ± 6%, reached after 1.8 h and 17.5 h on stream for the parent ZSM-48 and the MCC, respectively, the two samples exhibit comparable coke content of 3.4 and 4.0 wt%, respectively. Correspondingly, only 1.5 wt% of coke is deposited within the MCC after 1.8 h. This indicates that the lifetime enhancement of the MCC system stems primarily from a reduced rate of coke formation rather than from an increased coke accumulation capacity. The slightly higher coke content of the MCC is ascribed to the presence on the Pd/SiO_2, which may also undergo some fouling.

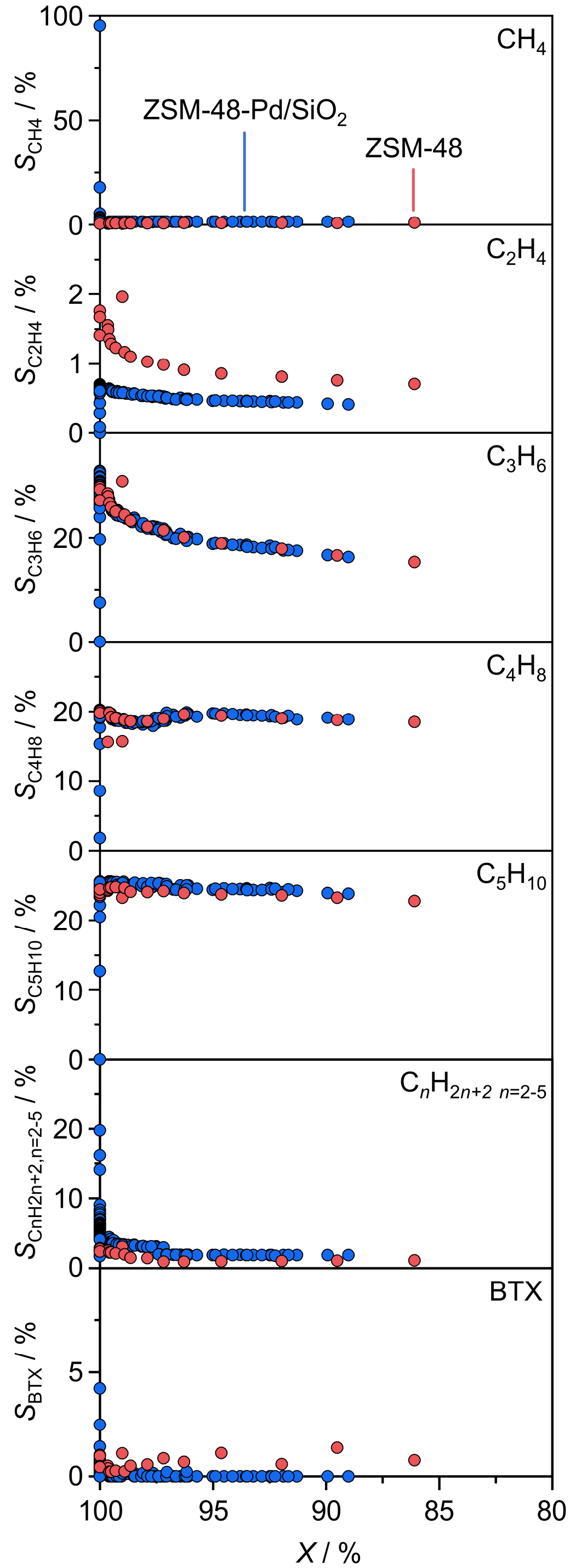

Supplementary Fig. 11. Selectivity profile of ZSM-48-Pd/SiO$_2$ MCC. Selectivity to specific hydrocarbons (S_{CxHy}) vs. conversion (X) profiles in MTH reaction with H_2 co-feed over parent ZSM-48 and the ZSM-48-Pd/SiO$_2$ MCC, for selected hydrocarbon products. Reaction conditions are detailed in **Fig. 2** of the main manuscript. Similarly to the ZSM-22 case (**Supplementary Fig. 4**), the selectivity profile over the MCC system follows that of the parent

zeolite, while showing greatly reduced contribution of aromatic-cycle reactions (lower S_{C2H4} and S_{BTX}), and some over-hydrogenation of alkene products, particularly at the reaction onset (higher $S_{CnH2n+2,n = 2-5}$).

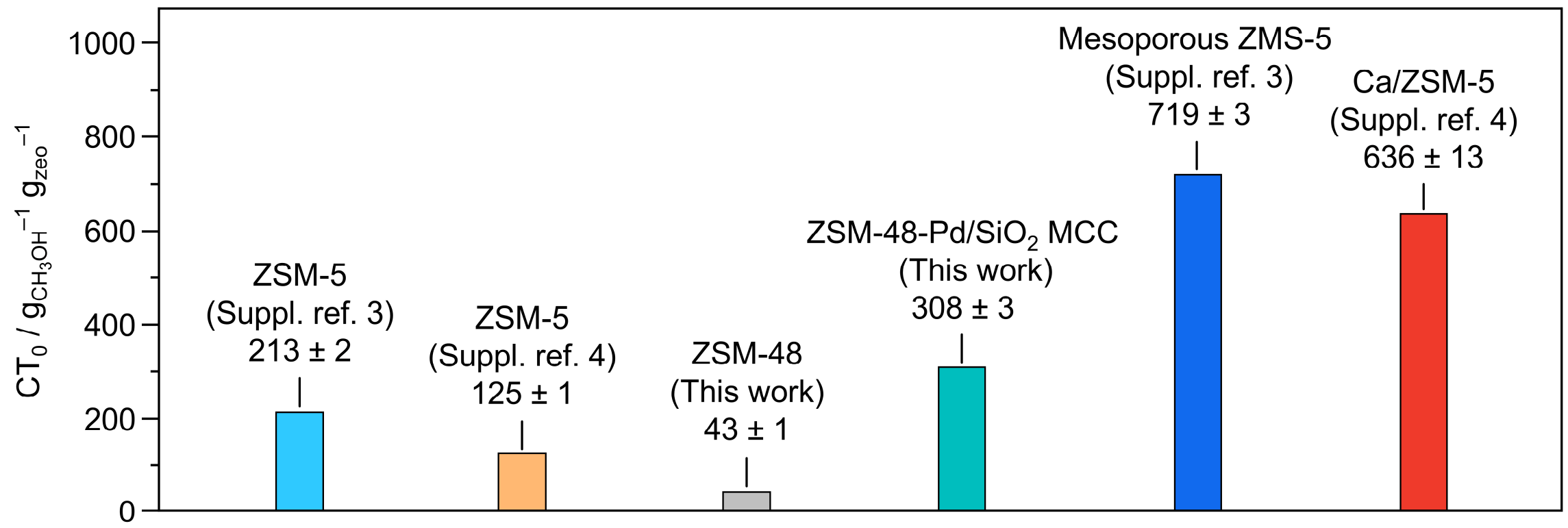

Supplementary Fig. 12. Performance of ZSM-48-Pd/SiO_2 MCC relative to representative ZSM-5 catalysts. Comparison between the cumulative methanol turnover (CT_0) achieved in MTH at near ambient pressure over ZSM-48-Pd/SiO_2 MCC and the values reported for notable examples of modified ZSM-5 catalysts, such as mesoporous ZSM-5 and Ca-exchanged ZSM-5, and the corresponding parent zeolites[3,4]. Uncertainty intervals correspond to the error of the fit (**Supplementary Methods 1**). While 1D 10-MR zeolites typically exhibit substantially lower CT_0 values than ZSM-5 catalysts, the MCC system based on ZSM-48 achieves a CT_0 of 310 g_{CH3OH} g_{zeo}^{-1}. This value approaches those of modified-ZSM-5 systems (≈ 600-700 g_{CH3OH} g_{zeo}^{-1}) and exceeds that of the corresponding parent zeolites (120-200 g_{CH3OH} g_{zeo}^{-1}), underscoring the potential of the MCC approach for practical scale-up of the MTH process using 1D zeolite topologies. Reaction conditions: CH_3OH:H_2=10:90, WHSV = 4.8 g_{CH3OH} g_{zeo} h^{-1}, T = 723K, and P = 1.2 bar (MCC and related parent ZSM-48); CH_3OH:N_2=50:50, WHSV = 8.0 g_{CH3OH} g_{zeo} h^{-1}, T = 773 K, and P = 1.0 bar (Ca/ZSM-5 and corresponding parent ZSM-5); CH_3OH:N_2=55:45, WHSV = 9.5 g_{CH3OH} g_{zeo} h^{-1}, T = 723 K, and P = 1.0 bar (mesoporous ZSM-5 and corresponding parent ZSM-5).

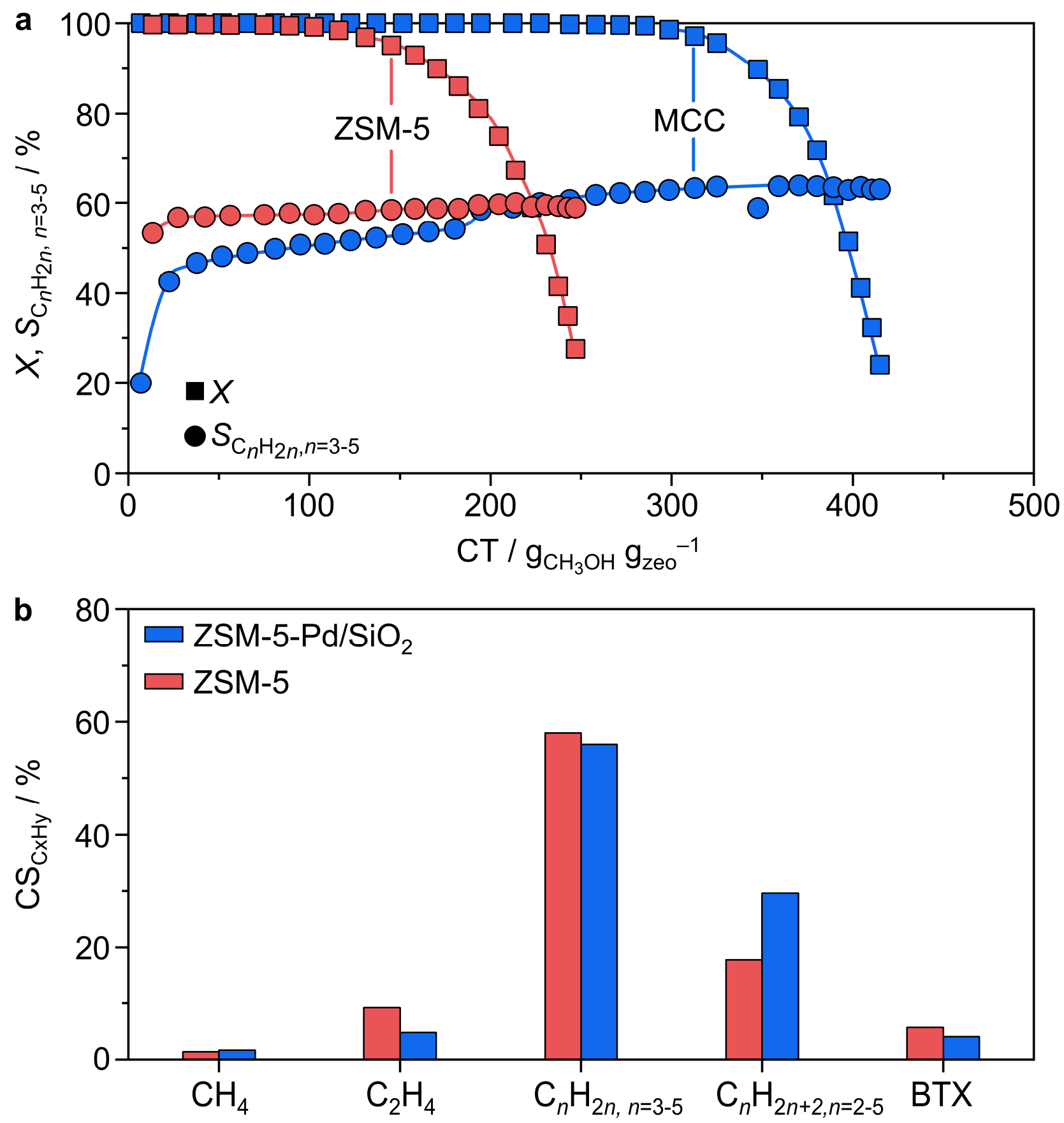

Supplementary Fig. 13. MTH performance of ZSM-5-Pd/SiO$_2$ MCC. a Methanol conversion (X) and selectivity toward C$_3$-C$_5$ alkenes ($S_{C_nH_{2n}}$, n=3-5) vs. methanol cumulative turnover (CT), and **b** cumulative selectivities to specific hydrocarbons (CS_{CxHy}) of the parent ZSM-5 (Zeolyst International, CBV 5524G) and ZSM-5-Pd/SiO$_2$ MCC. The MCC exhibits a lifetime improvement by a factor of ×1.6, with a CT$_0$ of 434 g_{CH3OH} g_{zeo}^{-1}, compared to 274 g_{CH3OH} g_{zeo}^{-1} for the parent zeolite. The $CS_{C_nH_{2n},\, n=3\text{-}5}$ of the parent ZSM-5 (58%) is slightly reduced in the MCC (56%), reflecting the trend observed at the beginning of the test. The decrease in CS_{C2H4} (from 9.2 to 4.7%) and CS_{BTX} (from 5.6 to 3.9%) points to a partial suppression of the aromatic cycle in the MCC, while the marked rise in $CS_{C_nH_{2n+2},\, n=2\text{-}5}$ (from 17.8 to 29.6%) results from over-hydrogenation of the alkene products. Reaction conditions: CH_3OH:H_2=10:90, WHSV = 38 g_{CH3OH} g_{zeo} h^{-1}, W_{PdSiO2}:$W_{zeolite}$ = 4, T = 723K, and P = 1.4 bar.

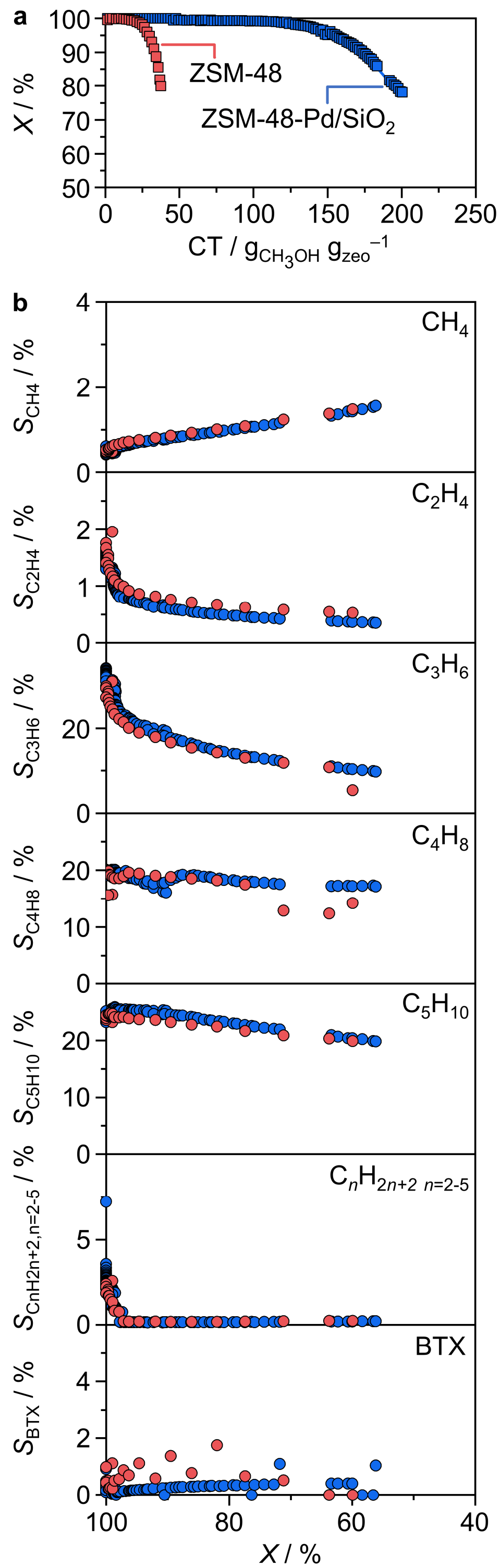

Supplementary Fig. 14. Selectivity profile of ZSM-22-Pd/SiO$_2$ MCC with reduced SCC loading. a Conversion (X) vs. methanol cumulative turnover (CT), and **b** corresponding selectivity (S_{CxHy}) vs. X profiles in MTH reaction with H_2 co-feed over the parent ZSM-48 and the ZSM-48-Pd/SiO$_2$ MCC with $W_{Pd/SiO2}$:$W_{zeolite}$ = 0.2. Other reaction conditions are detailed

in **Fig. 2** of the main manuscript. Reducing the amount of Pd/SiO_2 SCC has no significant effect on the selectivity profile of the MCC, except for the elimination of the induction period observed at the onset of the reaction (**Supplementary Fig. 11**).

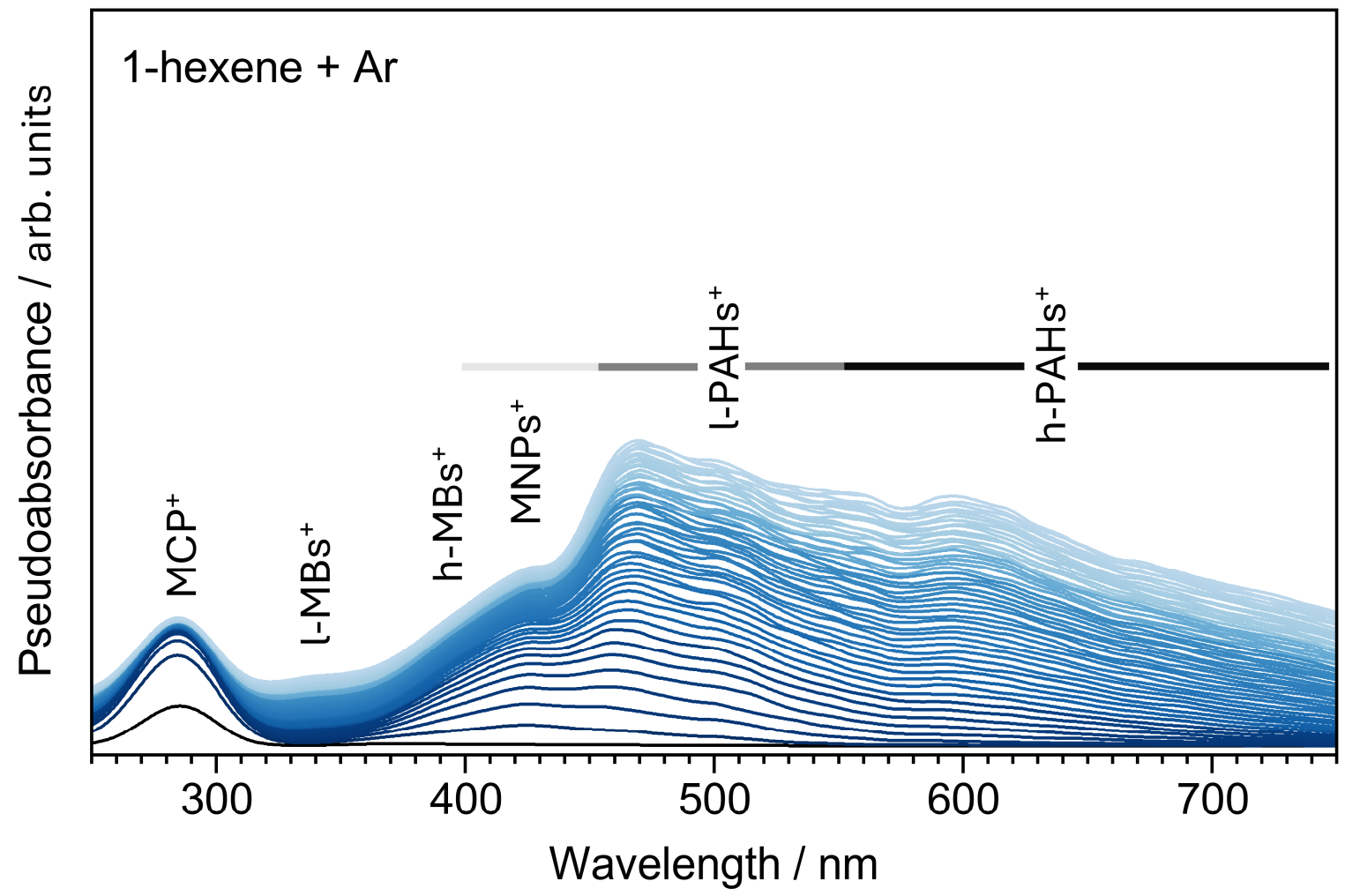

Supplementary Fig. 15. DR-UV-vis spectra acquired under CH_3OH-free conditions, during the cracking of 1-hexene over ZSM-22. The spectroscopic signature of methylcyclopentenyl (MCP^+) species is the most pronounced at the onset of the reaction, rapidly achieving a steady-state pseudoabsorbance, pointing to their rapid consumption. Contrarily, bands corresponding to monocyclic and polycyclic aromatics rise steadily, owing to their progressive accumulation within the zeolite. These findings suggest that MCP^+ species are in dynamic equilibrium with alkenes (or their corresponding carbenium ions) and do not accumulate. Reaction conditions: 1-hexene:Ar = 25:75 mol%, WHSV = 12 g_{C6H12} g_{zeo} h^{-1}, T = 723 K, and P = 1.0 bar.

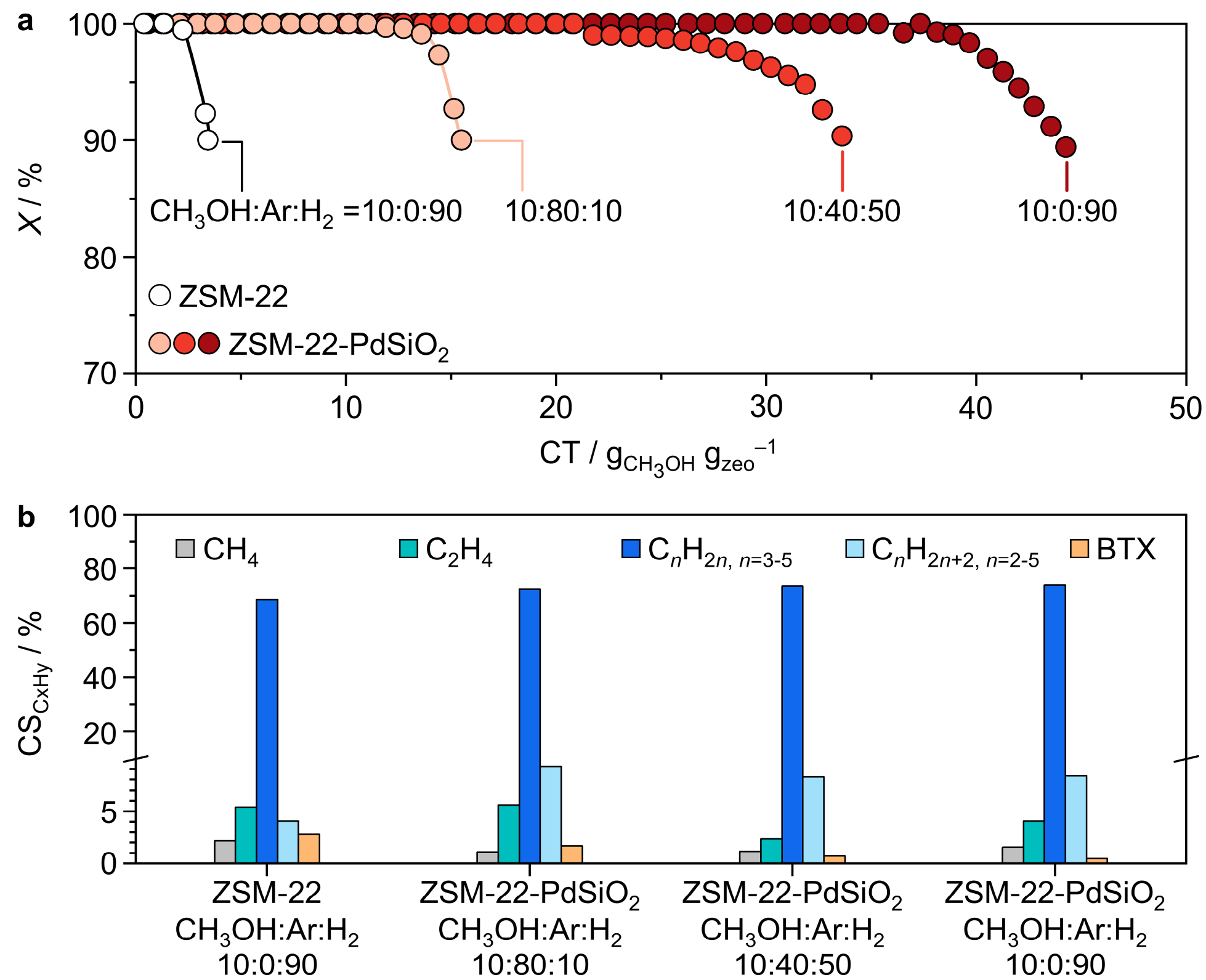

Supplementary Fig. 16. a Impact of the hydrogen concentration on the catalytic performance of the ZSM-22-Pd/SiO$_2$ MCC. Methanol conversion (*X*) vs. methanol cumulative turnover (CT), and **b** corresponding cumulative selectivity to specific hydrocarbons (CS_{CxHy}) over the parent ZSM-22 and the ZSM-22-Pd/SiO$_2$ MCC (W_{PdSiO2}:$W_{zeolite}$ = 1), at different concentrations of H_2 co-feed (10-90 mol%). Reaction conditions are detailed in **Fig. 4** of the main manuscript. Increasing the H_2 concentration extends the MCC lifetime, while retaining a cumulative selectivity profile similar to that of the parent zeolite.

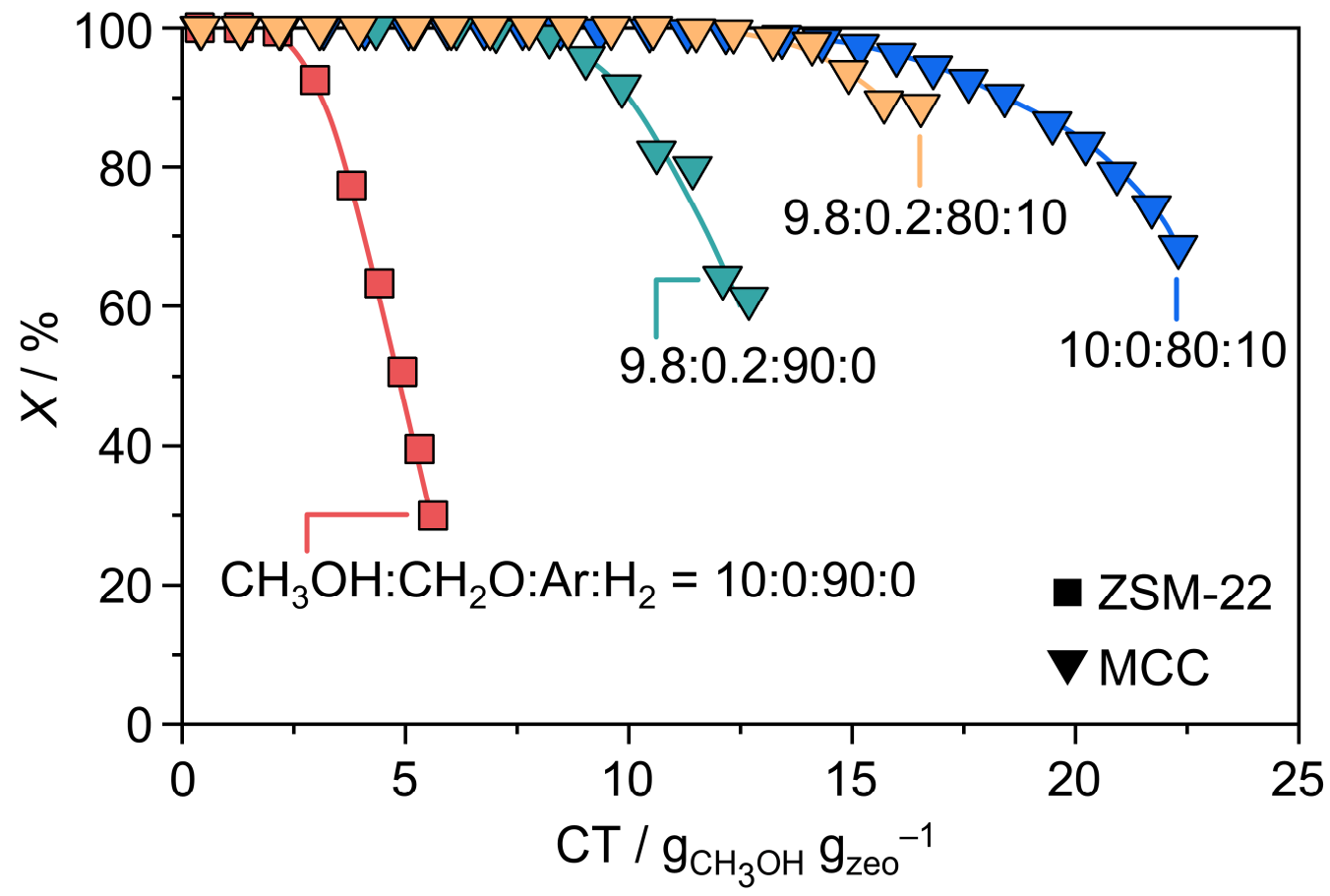

Supplementary Fig. 17. Impact of the use of SCC and H_2 co-feed on coke-induced deactivation. Methanol conversion (*X*) vs. methanol cumulative turnover (CT) in MTH with H_2 co-feed over parent ZSM-22 and ZSM-22-Pd/SiO_2 MCC, with and without CH_2O co-feeding (c_{CH2O} ≈ 2 mol$_C$% vs. CH_3OH). In the presence of H_2, the addition of CH_2O to the reactant feed has only a minor effect on the MCC stability. Notably, even in the absence of H_2, the MCC outperforms the parent ZSM-22 despite the CH_2O co-feed. Reaction conditions: CH_3OH:CH_2O:Ar:H_2 = 10(9.8):10(0):0(0.2):80(90) mol%, WHSV = 2.4 g_{CH3OH} g_{zeo} h^{-1}, $W_{Pd/SiO2}$:$W_{zeolite}$ = 2, T = 723 K, and P = 1.2 bar.

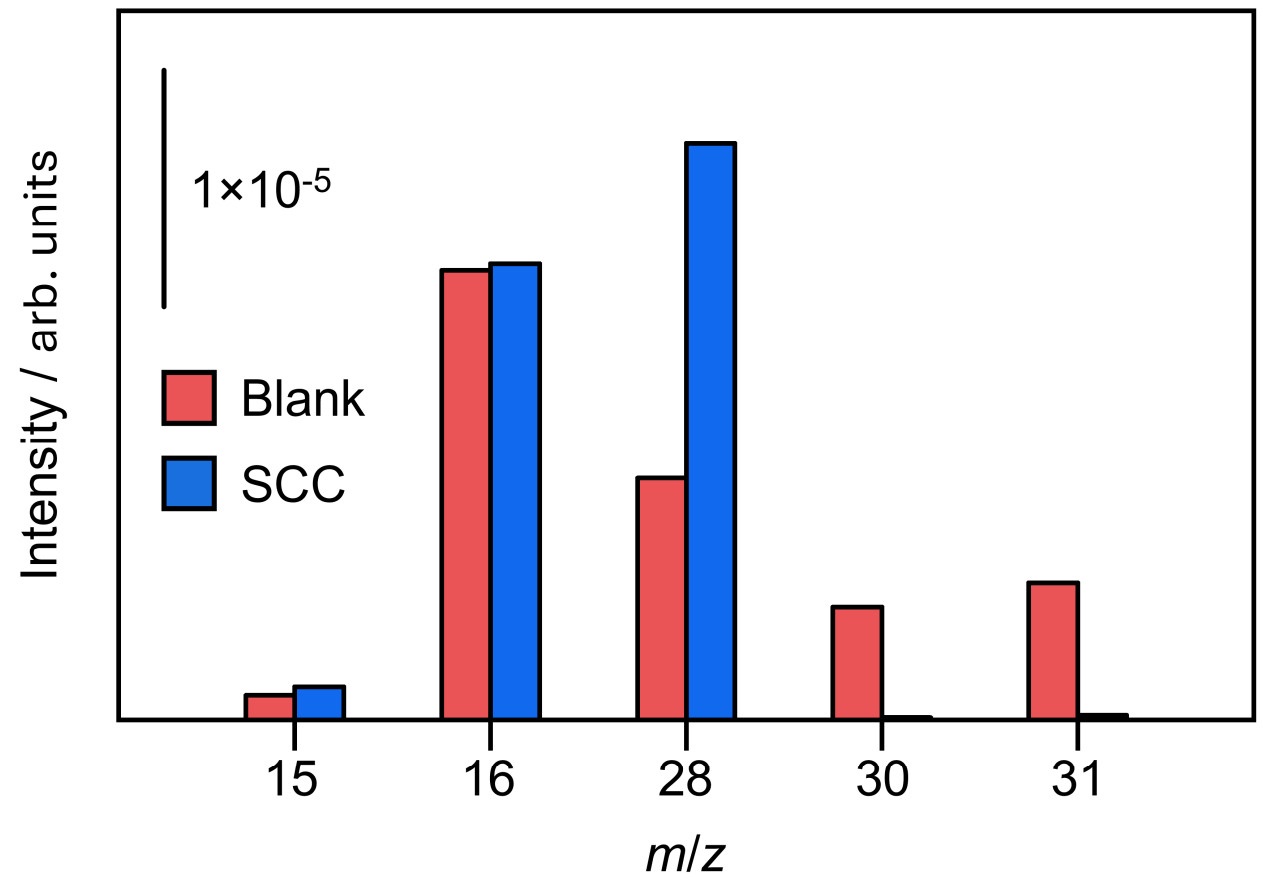

Supplementary Fig. 18. Reactivity of CH_2O over Pd/SiO_2 SCC with H_2 co-feed. MS fragmentation pattern from the reaction of CH_3OH in H_2 over Pd/SiO_2 SCC, and comparison with the corresponding blank. A mixture of CH_2O and H_2O diluted in H_2 (F_T = 56 cm^3_{STP} min^{-1}, $c_{CH2O} \approx 0.1$ mol%) was fed to the reactor packed with Pd/SiO_2 SCC mixed with quartz sand (W_{PdSiO2} = 50 mg, W_{sand} = 600 mg). CH_2O was generated *in situ* by introducing through the evaporator an aqueous solution of 1,3,5-trioxane. The CH_2O concentration of 0.1 mol% corresponds to the values typically observed in MTH[8]. The measured fragmentation pattern was compared with the blank acquired through a bypass line. The drop in *m/z* 30 signal with respect to the blank indicates full CH_2O consumption, with the rise in *m/z* 28 pointing to CH_2O decomposition into CO and H_2. Minor methanation occurs, as indicated by the small rise in *m/z* 15 and 16. The presence of CH_4 was corroborated by parallel GC-FID quantification, resulting in $S_{CH4} \approx 5\%$. Reaction conditions: CH_2O:H_2O:H_2 = 0.1:11.0:88.9 mol%, WHSV = 0.1 g_{CH2O} $g_{Pd/SiO2}$ h^{-1}, T = 723 K, and P = 1.1 bar. The signal is averaged over 60 minutes.

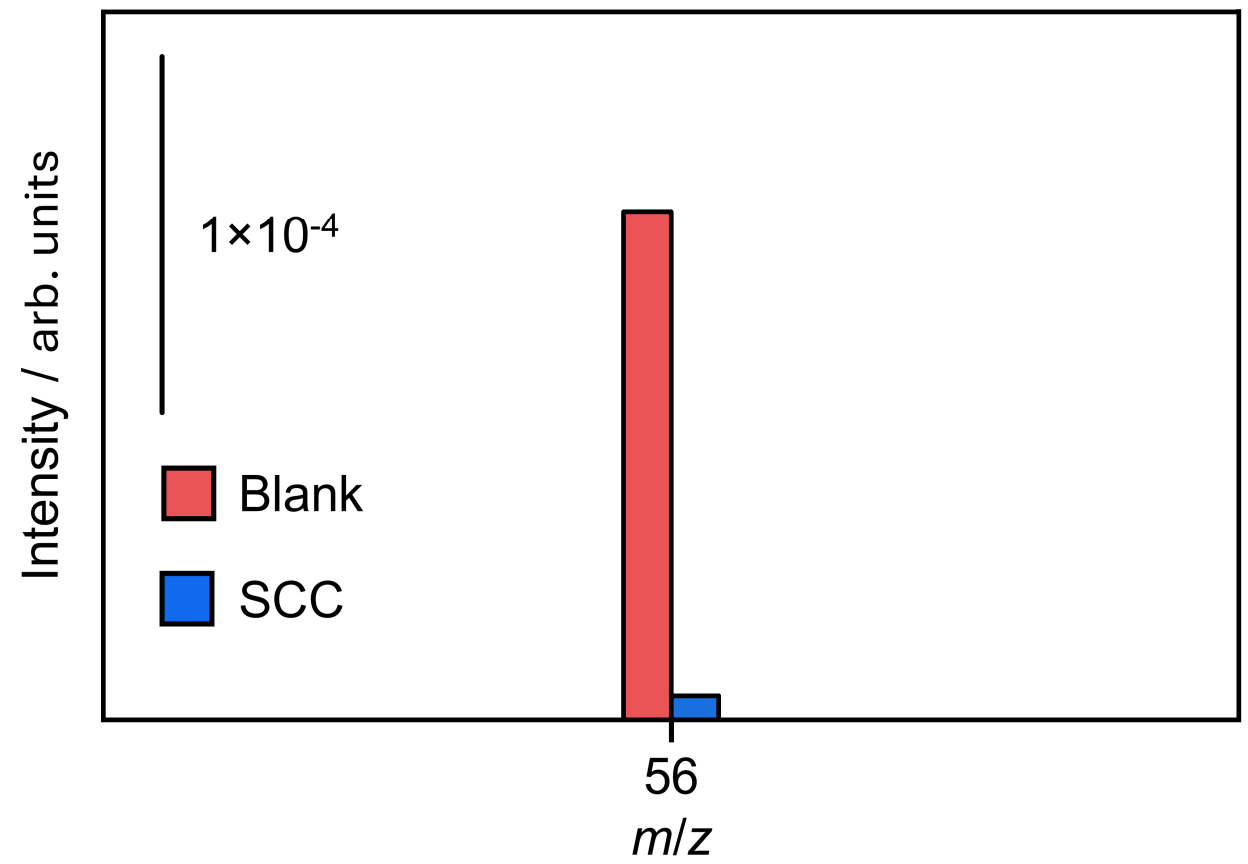

Supplementary Fig. 19. Reactivity of 1-butene over Pd/SiO$_2$ SCC with H$_2$ co-feed. Intensity of the *m*/*z* 56 fragment from the hydrogenation of 1-butene (C_4H_8) over Pd/SiO$_2$ SCC, and comparison with the corresponding blank. A mixture of C_4H_8 diluted in H_2 (F_{C4H8} = 1.4 cm^3_{STP} min^{-1}, F_T = 51 cm^3_{STP} min^{-1}, c_{C4H8} ≈ 2.7 mol%) was fed to the reactor packed with Pd/SiO$_2$ SCC mixed with quartz sand (W_{PdSiO2} = 50 mg, W_{sand} = 600 mg). The measured fragmentation pattern was compared with the blank acquired through the bypass line. The drop in the molecular fragment *m*/*z* 56 indicates extensive butene conversion. Separate GC-FID analysis confirmed full hydrogenation to *n*-butane. Reaction conditions: C_4H_8:H_2 = 2.6:97.4 mol%, WHSV = 4.1 g_{C4H8} $g_{Pd/SiO2}$ h^{-1}, T = 723 K, and P = 1.1 bar. The signal is averaged over 15 minutes.

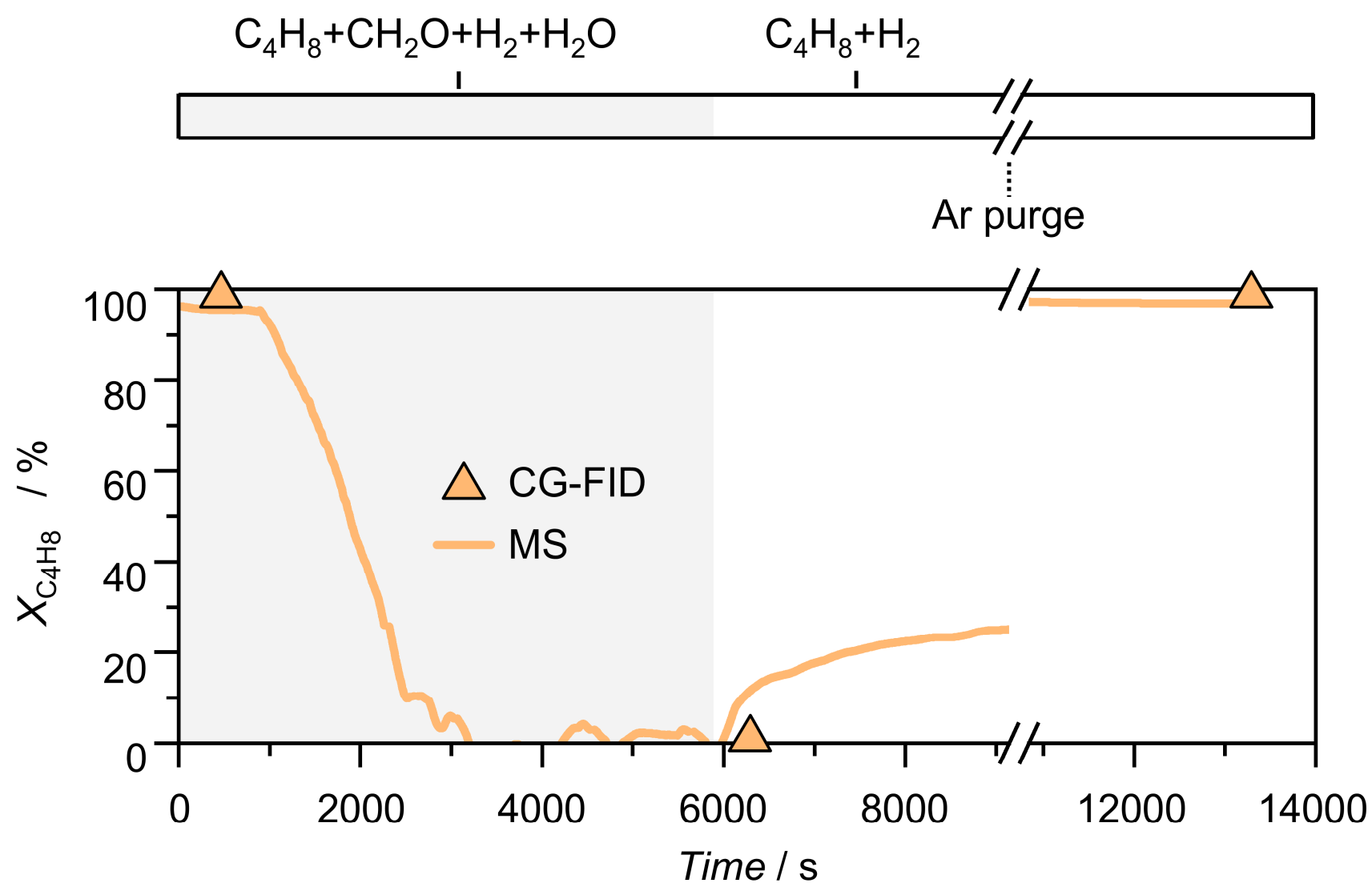

Supplementary Fig. 20 Reactivity of mixed 1-butene and oxygenates over Pd/SiO_2 SCC with H_2 co-feed. Butene conversion (X_{C4H8}) vs. time for the hydrogenation of the C_4H_8+CH_2O+H_2O mixture over Pd/SiO_2 SCC. A mixture of 1-butene, CH_2O, and H_2O diluted in H_2 (F_{C4H8} = 1.4 cm^3_{STP} min^{-1}, F_T = 62 cm^3_{STP} min^{-1}, c_{CH2O} ≈ 1.0 mol_C% vs. C_4H_8) was fed to the reactor packed with Pd/SiO_2 SCC mixed with quartz sand (W_{PdSiO2} = 50 mg, W_{sand} = 600 mg). CH_2O was generated *in situ* by introducing through the evaporator an aqueous solution of 1,3,5-trioxane. Initially, butene is fully hydrogenated to butane, in line with **Supplementary Fig. 19**. After a short induction period (ca. 15 min), the signal of *m*/*z* 56 begins to increase, indicating a decrease in C_4H_8 conversion. Parallel GC-FID analysis corroborates this finding, revealing a complete loss of the hydrogenation activity that persists even when the feed composition is switched to butene-only. After purging the reactor with Ar for an additional 50 min, the hydrogenation activity toward butene is restored. Reaction conditions: C_4H_8:CH_2O:H_2O:H_2 = (2.1-2.6):(0.1-0):(19.2-0):(78.6-97.4) mol%, WHSV = 4.1 g_{C4H8} $g_{Pd/SiO2}$ h^{-1}, T = 723 K, and P = 1.1 bar.

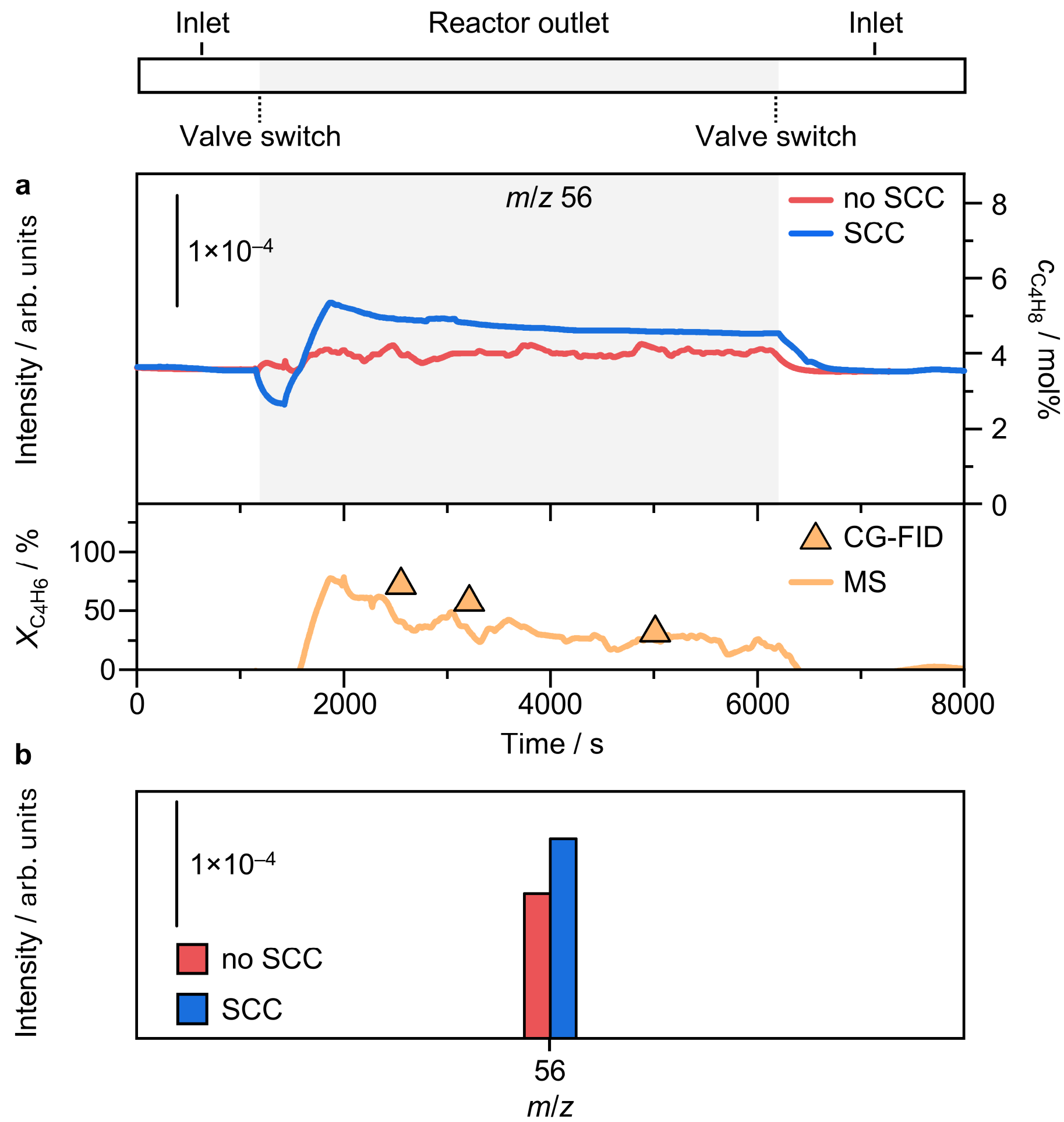

Supplementary Fig. 21. Reactivity of mixed 1-butene and butadiene over Pd/SiO_2 SCC with H_2 co-feed. a Evolution of the *m*/*z* 56 fragment from the hydrogenation of the C_4H_8+C_4H_6 mixture over Pd/SiO_2 SCC, and corresponding butadiene conversion (X_{C4H6}) vs. time profile. A mixture of butene (C_4H_8) and butadiene (C_4H_6) diluted in H_2 (F_{C4H8} = 1.4 cm^3_{STP} min^{-1}, F_T = 52 cm^3_{STP} min^{-1}, $c_{C4H6} \approx 36$ mol_C% vs. C_4H_8) was fed to the reactor, with online monitoring of the molecular fragment *m*/*z* 56. Butadiene was generated *in situ* by dehydration of 3-buten-1-ol. For this purpose, a layer of activated Al_2O_3 (Sigma Aldrich, W_{Al2O3} = 50 mg) was added in the reactor on top of the bed of Pd/SiO_2 SCC and quartz sand (W_{PdSiO2} = 50 mg, W_{sand} = 600 mg). An independent blank experiment was performed with Al_2O_3 only (no SCC). The inlet signal recorded through the bypass line before and after feeding the mixture to the reactor (valve switches) is also shown for reference purpose. **b** Intensity of the *m*/*z* 56 fragment and comparison with the blank experiment. The signal is averaged over ca. 80 minutes (corresponding to the region highlighted in gray in (**a**)). Contrary to the butene-only case, the sudden rise in the *m*/*z* 56 fragment over the Pd/SiO_2 SCC (**a**) indicates a net increase in the

concentration of butene, due to the selective hydrogenation of butadiene, with initial conversion $X_{C4H6} \approx 75\%$. Parallel GC-FID quantification corroborates this observation, with butane remaining below the detection limit. Reaction conditions: C_4H_8:C_4H_6:H_2 = 2.6:1.5:96.0 mol%, WHSV = 4.1 g_{C4H8} $g_{Pd/SiO2}$ h^{-1}, T = 723 K, and P = 1.1 bar.

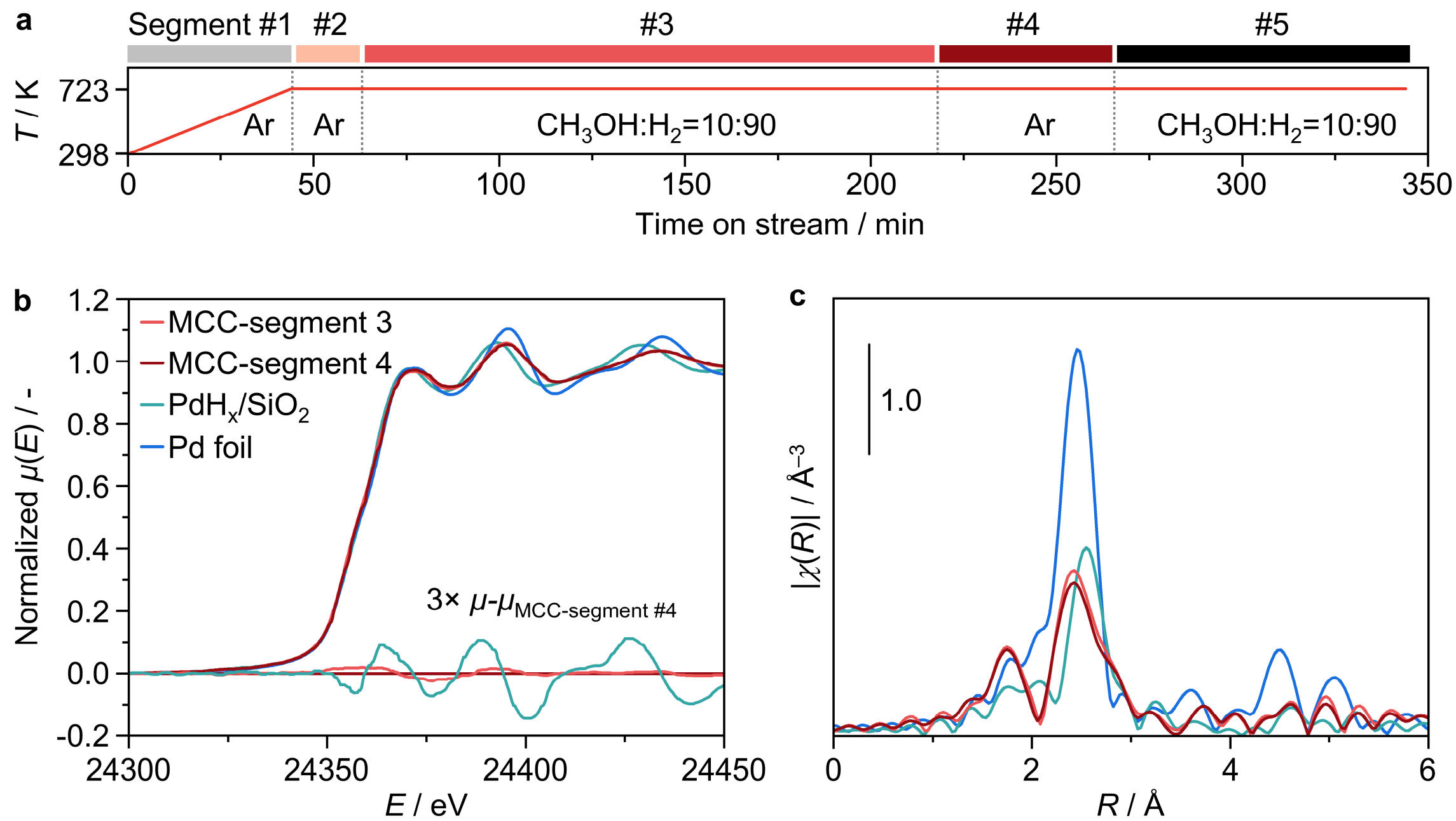

Supplementary Fig. 22. Sequence of reactive environments to which the ZSM-48-Pd/SiO_2 MCC catalyst is exposed during the *operando* Pd K-edge XAS experiment. **b** *Operando* Pd K-edge XANES spectra of the MCC averaged over segments #3 and #4, as shown in (**a**), compared to other Pd references. PdH_x was obtained by averaging the spectra acquired below $T = 440$ K during cooling of the MCC from 723 K to room temperature under the H_2-rich reactant atmosphere, to enable hydride formation. The difference spectra shown at the bottom are obtained by subtracting the spectrum of the MCC under Ar (segment #4) from the other spectra. **c** Corresponding Fourier-transformed data (not phase corrected). The spectra of the MCC were multiplied by a factor 3× for visual comparison. Reaction conditions: WHSV = 0.7 g_{CH3OH} g_{zeo} h^{-1}, W_{PdSiO2}:$W_{zeolite}$ = 0.5, $T = 723$ K, and $P \approx 1.0$ bar. Under reaction conditions, Pd is present as Pd(0) and is indistinguishable from the state observed under Ar (segment #4), as evidenced by the difference spectrum. Comparison with the reference spectrum of PdH_x indicates that no hydride formation occurs at the reaction temperature of 723 K, despite the presence of H_2, in line with reports of the limited thermal stability of palladium hydride and carbide phases (typically < 423 K)[5-7]. These observations suggest that the induction period may be due to changes in the population of chemisorbed species at the Pd surface, rather than to restructuring of the Pd nanoparticles.

Supplementary references

1. Janssens, T. V. H., Svelle, S. & Olsbye, U. Kinetic modeling of deactivation profiles in the methanol-to-hydrocarbons (MTH) reaction: A combined autocatalytic–hydrocarbon pool approach. *J. Catal.* **308**, 122-130 (2013).

2. Treacy, M. M. & Higgins, J.B. Collection of simulated XRD powder patterns for zeolites, 5th revised edition, Elsevier, (2007).

3. Milina, M., Mitchell, S., Crivelli, P., Cooke, D. & Pérez-Ramírez, J. Mesopore quality determines the lifetime of hierarchically structured zeolite catalysts. *Nat. Commun.* **5**, 3922 (2014).

4. Yarulina, I. et al. Suppression of the aromatic cycle in methanol-to-olefins reaction over ZSM-5 by post-synthetic modification using calcium. *ChemCatChem* **8**, 3057-3063 (2016).

5. Ziemecki, S. B., Jones, G. A., Swartzfager, D. G. & Harlow, R. L. Formation of interstitial Pd-C phase by interaction of ethylene, acetylene, and carbon monoxide with palladium. *J. Am. Chem. Soc.* **107**, 4547-4548 (1985).

6. Yamauchi, Y., Kobayashi, H. & Hiroshi Kitagawa. Hydrogen storage mediated by Pd and Pt nanoparticles. *ChemPhysChem* **10**, 2566-2576, (2009).

7. Costley-Wood, L. G., Mohammed, K., Carravetta, M., Decarolis, D., Goguet, A., Kordatos, A., Vakili, R., Manyar, H., McPake, E., Skylaris, C.-K., Thompson, P., Gibson, E. K. & Wells, P. P. Towards the operational window for nitridic and carbidic palladium nanoparticles for directed catalysis. *ChemCatChem* **15**, e202300870 (2023).

8. Liu, Y. et al. Critical role of formaldehyde during methanol conversion to hydrocarbons. *Nat. Commun.* **10**, 1462 (2019).
