# Supplementary material for: Enhanced alkene productivity in methanol-to-hydrocarbons conversion using a secondary catalyst component and hydrogen co-feeds
Source: Nat Commun. 2026 Sep 23;17:11065. doi: 10.1038/s41467-026-77944-9 (PMC13691128; doi:10.1038/s41467-026-77944-9)
Supplement: Supplementary file 2 [file 41467_2026_77944_MOESM2_ESM.pdf]

nature portfolio

Peer Review File

Enhanced alkene productivity in methanol-to-hydrocarbons conversion using a secondary catalyst component and hydrogen co-feeds

Corresponding Author: Dr Vladimir Paunovic

This file contains all reviewer reports in order by version, followed by all author rebuttals in order by version.

Attachments originally included by the reviewers as part of their assessment can be found at the end of this file.

Version 0:

Reviewer comments:

Reviewer #1

(Remarks to the Author)
The authors report that, under H_2-rich co-feeding conditions, physically mixing the selected secondary catalyst component (SCC), Pd/SiO_2, with 1D-10MR zeolites (ZSM-22 and ZSM-48) markedly extends catalyst lifetime and enhances C_3–C_5 olefin formation during the MTH reaction. They attribute this improvement to the in-situ hydrogenation activity of Pd, which selectively removes coke precursors such as formaldehyde and dienes. The manuscript presents some interesting results. Some important points, however, need to be further addressed.
1. Need for catalytic performance comparison under H_2-free conditions
The discussion of the SCC effect is based entirely on catalytic tests performed under H_2-rich conditions (CH_3OH:H_2 = 10/90). Although the authors later include H_2-free MTH conditions in certain in situ spectroscopic experiments, a direct catalytic evaluation of the physical mixtures in the absence of H_2 is still mandatory. Only by comparing H_2-free and H_2-rich performance can the specific contribution of Pd-derived hydrogenation activity be rigorously isolated.
2. Insufficient evidence supporting the inferior performance of Pd/ZSM-22
The authors conclude that Pd/ZSM-22 performs worse than the physical mixture (Pd/SiO_2 + ZSM-22) due to possible acid site loss and/or pore blockage. However, this explanation currently lacks supporting data. To validate this claim, the authors should provide: quantitative acidity measurements (e.g., pyridine-FTIR, NH_3-TPD), BET surface area and pore volume, catalyst particle size. If Pd/ZSM-22 has larger particles or significantly reduced zeolite accessibility, this may independently shorten catalyst lifetime.
3. Clarification of the term “mass diffusion regime” (line 175)
The statement “within the mass diffusion regime achieved with particle sizes between 0.25–0.15 mm…” requires clarification. Does this mean that, under the authors’ reaction conditions, the catalytic performance is actually controlled by mass-transfer limitations? Or do the authors intend to say that external/internal diffusion limitations are negligible in this particle size range?
4. Coke characterization needs more quantitative validation
The DR-UV-vis analysis comparing the intensity ratio of “internal” versus “external” coke species is helpful but only semi-quantitative. To convincingly demonstrate the suppressing effect of SCC on total coke formation, additional quantitative measurements are recommended, such as: TGA for total coke amount, temperature-programmed oxidation (TPO) profiles of CO/CO_2 evolution, deconvolution of oxidation peaks to distinguish different coke types. These would provide more direct quantitative evidence supporting the manuscript’s conclusions.
5. Influence of methanol partial pressure must be discussed
When evaluating the effect of H_2 concentration on catalyst stability and product distribution, the methanol partial pressure was kept at 0.1 P_0, which is relatively low. It is well known that high methanol partial pressure strongly accelerates coke formation. Therefore, the improved catalyst lifetime observed at high H_2 ratios may partly arise from lower $P(CH_3OH)$ rather than H_2 alone. The authors should discuss this aspect explicitly and clarify to what extent the observed enhancement is separable from methanol pressure effects.
6. Missing direct in situ evidence of CH_2O and diene consumption
The authors infer that Pd/SiO_2 selectively hydrogenates formaldehyde and dienes based on co-feeding experiments that affect catalyst lifetime differently. This is plausible but still indirect. To strengthen this mechanistic claim, the authors should

provide or discuss: in situ detection and quantification of CH_2O/dienes (e.g., online MS, IR); comparative time-dependent profiles with/without SCC; kinetic analysis of intermediate consumption rates. Such data would directly demonstrate that SCC indeed accelerates the removal of these coke precursors.
7. Expected increase of instantaneous alkane selectivity
If Pd is hydrogenating CH_2O and dienes, it may also hydrogenate olefins. The authors propose that Pd transforms into a more “mild and selective” state during the induction period, limiting undesired over-hydrogenation. However, this raises the question: Should the instantaneous, not cumulative, alkane selectivity increase at the early stage or during the whole methanol conversion (given that Pd/ZSM-48 exhibits higher alkane production due to stronger hydrogenation activity)?
8. More definitive identification of Pd species is required
To substantiate the interpretation of Pd structural evolution, more comparison with reference spectra is necessary. The authors should consider including: standard XAS references of PdC_x, CO-poisoned Pd, or in situ FTIR/DRIFTS showing characteristic alkyl, carbonyl, or carbide features.
These additions would allow a more confident assignment of the active Pd species responsible for selective hydrogenation.
9. The PDF file contains duplicated main-text sections and lacks supplementary information
The submitted PDF includes two copies of the main manuscript body and does not include the Supporting Information (SI). The authors must correct the document and provide the full SI package.
Overall Recommendation
The manuscript presents some interesting findings in MTH catalysis. However, some key mechanistic claims require more rigorous experimental support or clearer discussion. I recommend a sustainable revision to address all points above.

Reviewer #2

(Remarks to the Author)
I co-reviewed this manuscript with one of the reviewers who provided the listed reports. This is part of the Nature Communications initiative to facilitate training in peer review and to provide appropriate recognition for Early Career Researchers who co-review manuscripts.

Reviewer #3

(Remarks to the Author)
The authors of the manuscript presented a novel catalyst design concept to enhance the productivity of C3-C5 alkenes in unidimensional 10-membered ring zeolites (ZSM-22 and ZSM-48) for MTH reaction by simply mixing the zeolite component with a secondary catalyst component (Pd/SiO2) under near ambient pressure with H2 co-feeding. The authors ascribed such performance enhancement to the coke resistance induced by SCC for the hydrogenation of coke precursors (formaldehyde, dienes, etc.). The proposed bifunctional catalyst design strategy is interesting and the experiments were systematically performed. The manuscript may be published after the following major issues being well addressed.

1) Several unidimensional 10-membered ring zeolites (ZSM-22 and ZSM-48) were employed to integrate SCC (Pd/SiO2) in order to inhibit the formation of coking and improve the lifetime of the catalysts. The unidimensional 10-membered ring parent zeolites suffer seriously from fast coking formation and deactivation, and they are seldom employed for the practical MTH reaction. It is possible to enhance the selectivity to higher alkenes just using parent ZSM-5 samples with high durable stability and lifetime. Why the authors did not test the integration of ZSM-5 samples with SCC? It would be more important and industrially practical for the MTH reaction.

2) The authors stated that the effect of SCC primarily arises from intercepting coke-forming pathways through the hydrogenation of coke precursors (CH2O, dienes, etc.). Why the hydrogenation of alkenes to alkanes can be inhibited on SCC? What is the difference between the hydrogenation activities of alkenes, dienes, and CH2O on SCC after induction?

3) The cyclopentenyl species was identified as the active HCP intermediates in ZSM-22. What are the dominating HCPs in ZSM-22? The authors have suggested that the olefinic cycle dominates the MTH reaction in ZSM-22. It would be better to differentiate the “active” and the “dominating” HCPs in different zeolites for methanol conversion.

4) The authors claimed that several different integration methods were compared with comparable Pd size distribution. However, it is 4.5 nm of the Pd/SiO2 SCC, and 2.8 nm of the Pd loaded on ZSM-22 zeolite. Are the sizes of Pd distribution indeed comparable?

5) Why Wzeolite:WPd/SiO2 = 1:2 for ZSM-48, and 1:1 for ZSM-22? Why different ratios were taken?

6) Why the influence of the loading of SCC was tested on ZSM-48, not on ZSM-22? Which one is the primary parent sample studied in this work?

Version 1:

Reviewer comments:

Reviewer #2

(Remarks to the Author)
I co-reviewed this manuscript with one of the reviewers who provided the listed reports. This is part of the Nature Communications initiative to facilitate training in peer review and to provide appropriate recognition for Early Career Researchers who co-review manuscripts.

Reviewer #3

(Remarks to the Author)
I am satisfied with the revisions and recommend the acceptance of the revised manuscript.

Open Access This Peer Review File is licensed under a Creative Commons Attribution 4.0 International License, which permits use, sharing, adaptation, distribution and reproduction in any medium or format, as long as you give appropriate credit to the original author(s) and the source, provide a link to the Creative Commons license, and indicate if changes were made.
In cases where reviewers are anonymous, credit should be given to 'Anonymous Referee' and the source.
The images or other third party material in this Peer Review File are included in the article’s Creative Commons license, unless indicated otherwise in a credit line to the material. If material is not included in the article’s Creative Commons license and your intended use is not permitted by statutory regulation or exceeds the permitted use, you will need to obtain permission directly from the copyright holder.
To view a copy of this license, visit https://creativecommons.org/licenses/by/4.0/

Manuscript NCOMMS-25-86258 – Response to Reviewers

Notes: Comments in blue - Replies in black - Actions in **bold** – Amendments to the manuscript in red.

Indicated page, line, figure, or reference numbers refer to the revised Manuscript and Supporting Information with changes highlighted.

Reviewer #1

The authors report that, under H_2-rich co-feeding conditions, physically mixing the selected secondary catalyst component (SCC), Pd/SiO_2, with 1D-10MR zeolites (ZSM-22 and ZSM-48) markedly extends catalyst lifetime and enhances C_3–C_5 olefin formation during the MTH reaction. They attribute this improvement to the in-situ hydrogenation activity of Pd, which selectively removes coke precursors such as formaldehyde and dienes. The manuscript presents some interesting results. Some important points, however, need to be further addressed.

We thank the Reviewer #1 for the interest in our paper and for the thoughtful comments, which we have addressed as detailed below.

1) Need for catalytic performance comparison under H_2-free conditions. The discussion of the SCC effect is based entirely on catalytic tests performed under H_2-rich conditions (CH_3OH:H_2 = 10/90). Although the authors later include H_2-free MTH conditions in certain in situ spectroscopic experiments, a direct catalytic evaluation of the physical mixtures in the absence of H_2 is still mandatory. Only by comparing H_2-free and H_2-rich performance can the specific contribution of Pd-derived hydrogenation activity be rigorously isolated.

We thank Reviewer #1 for the valuable suggestion. In order to isolate the effect of the SCC, the MCC and the parent zeolite were compared under the same reactant mixture (CH_3OH + H_2). This is important because H_2 alone can already affect the lifetime of the parent zeolite, as it can be activated on Brønsted acid sites, particularly at high pressure (see *Nat. Catal.* **2018**, *1*, 666-672). However, prompted by the Reviewer's suggestion, **we have conducted new catalytic tests under H_2-free conditions, which are now presented in the revised Supplementary Information file (please see Supplementary Fig. 8) and discussed in the revised manuscript (please see page: 11, lines: 4-12): "**The use of H_2 co-feeds instead of Ar slightly improves the $CP_{C_nH_{2n}, n=3\text{-}5}$ of parent ZSM-48 by 1.6× (**Supplementary Fig. 8**). The addition of Pd/SiO_2 SCC in the absence of H_2 co-feeding leads to higher enhancement by ≈ 4×, likely because small amounts of H_2 are always generated via minor CH_3OH decomposition on Pd (**Supplementary Fig. 9**). External H_2 co-feeding is required to maximize the productivity enhancement. Compared to the parent ZSM-48 tested with H_2, the combined use of SCC and H_2 co-feed increased the alkene productivity in the conversion range X = 100-90% by ≈ 7× (**Fig. 2a,b**). The enhancement factor reaches ≈ 12× when the parent zeolite without H_2 co-feed is used as the benchmark (**Supplementary Fig. 8**).**"**

2) Insufficient evidence supporting the inferior performance of Pd/ZSM-22. The authors conclude that Pd/ZSM-22 performs worse than the physical mixture (Pd/SiO_2 + ZSM-22) due to possible acid site loss and/or pore blockage. However, this explanation currently lacks supporting data. To validate this claim, the authors should provide: quantitative acidity measurements (e.g., pyridine-FTIR, NH_3-TPD), BET surface area and pore volume, catalyst particle size. If Pd/ZSM-22 has larger particles or significantly reduced zeolite accessibility, this may independently shorten catalyst lifetime.

We thank Reviewer #1 for highlighting a lack of evidence supporting this claim. **We have now assessed textural and acidic properties of the calcined and H_2-treated Pd/ZSM-22 catalyst, by N_2 sorption and NH_3-TPD, respectively (Supplementary Table 1, page: 6).** The new measurements reveal that surface area and micropore volume of the parent ZSM-22 are scarcely affected by Pd deposition, even after H_2 treatment. We further notice that the Pd particle size in Pd/ZSM-22 was already presented in **Figure 1f**, being slightly smaller than in Pd/SiO_2, 2.8 *vs.* 4.5 nm, respectively. Contrarily, NH_3 TPD measurements indicate a decreased concentration of stronger acid sites. **These results have been included in the amended Supporting Information file (please see Supplementary Table 1, page: 6, Supplementary Fig. 1, page: 8; and Supplementary Fig. 6, page: 15), and in the revised manuscript (please see page: 10, lines: 4-10): "**Pd/ZSM-22 exhibits comparable textural properties as the parent ZSM-22 (**Supplementary Table 1** and **Supplementary Fig. 6**) but a lower concentration of strong acid sites (by 23%). Considering smaller average Pd nanoparticle size in Pd/ZSM-22 compared to Pd/SiO_2 SCC (2.8 vs. 4.5 nm), which is expected to enhance the hydrogenation activity, its inferior performance in terms of over-hydrogenation and catalyst stability relative to the MCC suggests that close proximity of Pd to HCP active species is not optimal for leveraging the beneficial impact of the hydrogenating functionalities.**"**

3) Clarification of the term "mass diffusion regime" (line 175). The statement "within the mass diffusion regime achieved with particle sizes between 0.25–0.15 mm…" requires clarification. Does this mean that, under the authors' reaction conditions, the catalytic performance is actually controlled by mass-transfer limitations? Or do the authors intend to say that external/internal diffusion limitations are negligible in this particle size range?

We thank Reviewer #1 for highlighting this ambiguity**, which is clarified in our revised manuscript (please see page: 10, lines: 11-17): "**No pronounced differences were observed between the two MCC catalysts with different ZSM-22-SCC proximity in terms of turnover capacity and product selectivity, with a slightly slower deactivation of the grinded MCC at sub-complete conversion (**Supplementary Fig. 7**). This indicates that the positive effect is preserved when the SCC-zeolite proximity is reduced from tens of micrometers (mixed particles) to below 1 μm (mixed powders) and further suggests that this effect primarily arises from the interception of deactivating gas-phase species by the SCC.**"**

4) Coke characterization needs more quantitative validation. The DR-UV-vis analysis comparing the intensity ratio of "internal" versus "external" coke species is helpful but only semi-quantitative. To convincingly demonstrate the suppressing effect of SCC on total coke formation, additional quantitative measurements are recommended, such as: TGA for total

coke amount, temperature-programmed oxidation (TPO) profiles of CO/CO_2 evolution, deconvolution of oxidation peaks to distinguish different coke types. These would provide more direct quantitative evidence supporting the manuscript's conclusions.

We thank Reviewer #1 for suggesting direct quantification experiments. **We have now conducted additional TGA of the parent zeolite and MCC catalysts deactivated at different time on stream, which further supports that addition of SCC along with H_2 co-feed substantially reduces the rate of coke formation. These results are included in the revised manuscript (please see page: 16, lines: 13-15): "**A reduced rate of coke deposition over the MCC system is also confirmed by the independent quantification of the coke content in spent ZSM-48 and ZSM-Pd/SiO_2 MCC catalysts by thermogravimetric analysis (TGA, **Supplementary Fig. 10).**"

5) Influence of methanol partial pressure must be discussed. When evaluating the effect of H_2 concentration on catalyst stability and product distribution, the methanol partial pressure was kept at 0.1 P_0, which is relatively low. It is well known that high methanol partial pressure strongly accelerates coke formation. Therefore, the improved catalyst lifetime observed at high H_2 ratios may partly arise from lower $P(CH_3OH)$ rather than H_2 alone. The authors should discuss this aspect explicitly and clarify to what extent the observed enhancement is separable from methanol pressure effects.

The Reviewer correctly points out that methanol partial pressure (P_{CH3OH}) has an important influence on catalyst deactivation in MTH, as higher pressures (concentrations) can accelerate deactivation paths. However, there is no hidden influence of the methanol concentration behind the enhancement effect observed when comparing the parent zeolite and the MCC system, as all catalysts were compared under the same reactant composition CH_3OH:H_2 = 10:90 and at a fixed total pressure (P_T = 1.2 bar), and hence constant partial pressure of methanol. Additionally, in the experiments in which the H_2 concentration was varied (P_{H2} = 0.1-0.9P_T), P_{CH3OH} was kept constant at 0.1P_T (0.12 bar) by adding the appropriate flow of argon. **We have clarified this aspect in the revised manuscript (please see page: 16, lines: 19-21): "**In these experiments, the H_2 concentration was varied, while the CH_3OH concentration in the feed was kept constant at 10 mol%, with Ar used as balance gas (CH_3OH:H_2:Ar = 10:10-90:80-0 mol%).**"** We further highlight that in the newly added TGA experiments (please see the response to comment #4), the coked materials were prepared under accelerated deactivating conditions, using a higher CH_3OH concentration of 19 mol%, and that even under these conditions a strong lifetime enhancement is observed over the MCC compared to the parent ZSM-48, leading to 15× higher cumulative turnover. This finding has been highlighted in the revised manuscript **(please see page: 11, lines: 12-13): "**Furthermore, the beneficial effect of the SCC and H_2 co-feed is also observed at elevated inlet CH_3OH concentrations (**Supplementary Fig. 10**).**"**

6) Missing direct in situ evidence of CH_2O and diene consumption. The authors infer that Pd/SiO_2 selectively hydrogenates formaldehyde and dienes based on co-feeding experiments that affect catalyst lifetime differently. This is plausible but still indirect. To strengthen this

mechanistic claim, the authors should provide or discuss: in situ detection and quantification of CH_2O/dienes (e.g., online MS, IR); comparative time-dependent profiles with/without SCC; kinetic analysis of intermediate consumption rates. Such data would directly demonstrate that SCC indeed accelerates the removal of these coke precursors.

We thank the Reviewer for highlighting the need to strengthen the experimental evidence behind our mechanistic interpretation and agree that direct detection would be insightful. However, quantification of formaldehyde (CH_2O) in the methanol-containing reactant mixture is at the moment not possible, as the only technique capable of resolving the concentration of this intermediate in the complex MTH reaction mixture is PEPICO spectroscopy (see e.g., *ACS Catal.* **2024**, *14*, 463–474; *Catal. Sci. Technol.* **2024**, *14*, 1216-1228). The dedicated VUV beamline at the Swiss Light Source 2.0 is currently in shut down mode and will be operational only in late 2027. Additionally, a high reactivity of CH_2O in the presence of hydrocarbons (at medium-to-high conversion in MTH) makes it extremely challenging to elucidate the precise mechanism of formaldehyde consumption, and particularly distinguishing between its reaction with hydrocarbons (e.g. Prins condensation) *vs.* decomposition and/or hydrogenation over Pd.

Nonetheless, to strengthen the mechanistic understanding and prompted by the Reviewer's inquiry, **we have conducted a series of new MS experiments. In these, we investigate (*i*) the reactivity of individual species (CH_3OH, CH_2O, butene) over the SCC (Pd/SiO_2) with H_2 co-feed, and (*ii*) that of model mixtures (CH_2O + butene and butadiene + butene).** These experiments have clarified that CH_2O is primarily decomposed in presence of Pd/SiO_2, with minor methanation, while dienes can be selectively hydrogenated to alkenes. **The description of these experiments and their interpretation has been included in the Supplementary Information file (please see Supplementary Methods 2 and Supplementary Fig. 9, 18-21) and in the revised manuscript (please see page: 19, lines: 1-9): "**These results indicate that the promoting effect of SCCs stems from their ability to intercept the species promoting the formation of coke and that this likely proceeds via selective hydrogenation, as also reflected by the dependency of the CT enhancement factor on the H_2 pressure. Investigations of the reactivity of CH_2O and of mixed butadiene-butene feeds over the Pd/SiO_2 SCC in the presence of H_2 show that CH_2O decomposes into CO and H_2, with minor methane formation, whereas butadiene is selectively hydrogenated to butene (**Supplementary Fig. 18-21**). These findings reveal that different deactivating intermediates can follow distinct pathways, with polyenes and possibly other unsaturated species being responsible for the observed dependence on H_2.**"** Our new experiments have further highlighted that the selective hydrogenation behavior of the Pd/SiO_2 SCC can be triggered by the presence of oxygenates and polyene species within the MTH mixture. **The description of these experiments and their interpretation has been included in the Supplementary Information file (please see Supplementary Fig. 20-21) and the findings highlighted in the revised manuscript (please see page: 23, lines: 13-21): "**To gain insights into the influence of selected species over the hydrogenation activity of the Pd/SiO_2 SCC, a series of experiments were performed feeding model mixtures of 1-butene, H_2, and a co-reactant, chosen between CH_2O or butadiene, in the absence of the zeolite catalyst (**Supplementary Fig. 19-21**). While pure streams of 1-butene in H_2 undergo complete hydrogenation on Pd/SiO_2 at 723 K, both oxygenates and butadiene were found to inhibit butene consumption. Furthermore, butadiene is selectively hydrogenated in presence of butene. Despite simplifying

the complex composition of the MTH product mixture, these experiments indicate that species formed during MTH can prevent over-hydrogenation over SCC, thus steering the hydrocarbon selectivity toward alkenes.**"**

7) Expected increase of instantaneous alkane selectivity**.** If Pd is hydrogenating CH_2O and dienes, it may also hydrogenate olefins. The authors propose that Pd transforms into a more "mild and selective" state during the induction period, limiting undesired over-hydrogenation. However, this raises the question: Should the instantaneous, not cumulative, alkane selectivity increase at the early stage or during the whole methanol conversion (given that Pd/ZSM-48 exhibits higher alkane production due to stronger hydrogenation activity)?

The Reviewer is correct. During the induction period, when the olefin selectivity is rising and the MCC system still exhibits very hydrogenative behavior, the selectivity to methane and alkanes is correspondingly higher and drops at increasing time on stream (*tos*). Selectivity *vs.* *tos* plots reported in the Supplementary Information file clearly show this trend (**please see Supplementary Fig. 4** and **11**). We have emphasized this observation in the amended manuscript **(please see page: 11, lines: 17-21, and page: 12, lines: 1): "**Similarly to ZSM-22, the selectivity profiles (**Supplementary Fig. 11**) reveal a short induction period at the early stage of the reaction, characterized by extensive CH_4 and alkane formation. This suggests methanol decomposition over Pd to carbon monoxide and hydrogen, followed by hydrogenation into CH_4, as well as unselective alkene hydrogenation to alkanes (*vide infra*; **Supplementary Fig. 9**).**"** As the Reviewer observes in the comment #10, the Supplementary Information file was not correctly included among the available files during the initial submission. We apologize for this inconvenience and provide it now together with the revised manuscript. We highlight that Pd/SiO_2 SCC reactivity was additionally studied via probe CH_2O and butadiene experiments and the *operando* XAS analysis discussed in our responses to the comments #6 and #8 of Reviewer #1, respectively.

8) More definitive identification of Pd species is required. To substantiate the interpretation of Pd structural evolution, more comparison with reference spectra is necessary. The authors should consider including: standard XAS references of PdC_x, CO-poisoned Pd, or in situ FTIR/DRIFTS showing characteristic alkyl, carbonyl, or carbide features.

We thank Reviewer #3 for highlighting the need to deepen our XAS analysis. Prompted by their inquiry, **we have carried out *operando* Pd K-edge XAS measurements of the MCC system.** *Operando* data overcome the limitations of *ex situ* measurements and provide a comprehensive description of the on-stream evolution of the MCC. Notably, the *operando* XAS experiments ruled out the formation of palladium hydrides and/or carbide phases at the temperature used in the reaction (T = 723 K). **These new results are discussed in our revised manuscript (please see page: 20, lines: 4-7 and 17-25; page: 23, lines: 1-5): "***Operando* Pd K-edge XAS indicates that PdO present in the calcined SCC is reduced to metallic Pd during temperature ramping in Ar and exposure to the H_2 co-feed (**Fig. 5**). Consistent with the tests performed in conventional microreactor, the temporal profile of alkene-specific fragment at *m*/*z* 41 exhibits a short induction period (**Fig. 5c**), which is also

observable during a second MTH reaction that follows an intermediate Ar treatment (≈ 60 min). Notably, Pd(0) state remains unaltered during cycles of reactive (CH_3OH+H_2) and inert atmosphere treatments (**Fig. 5a,b**), excluding the occurrence of deep structural changes of the Pd phase induced by reacting environment (**Supplementary Fig. 22**). The absence of detectable reaction-induced restructuring suggests that the modulation of Pd hydrogenation activity that enables the formation of alkenes with high selectivity, is likely originating from the surface-confined effects, such as competitive adsorption of strongly binding intermediates (e.g. polyenes) on Pd(0) and poisoning species that accumulate during MTH. These results are consistent with the suppression of alkene over-hydrogenation induced by oxygenates and dienes in the MTH mixture (*vide infra*, **Supplementary Fig. 19-21**).**"**

9) The PDF file contains duplicated main-text sections and lacks supplementary information. The submitted PDF includes two copies of the main manuscript body and does not include the Supporting Information (SI). The authors must correct the document and provide the full SI package.

We sincerely apologize for this inconvenience. During the submission, the manuscript in PDF format as well as the SI file were both submitted as supplementary materials, and only the first was delivered. **We apologize and provide the revised Supplementary Information file with changes highlighted.**

Reviewer #2

I co-reviewed this manuscript with one of the reviewers who provided the listed reports. This is part of the Nature Communications initiative to facilitate training in peer review and to provide appropriate recognition for Early Career Researchers who co-review manuscripts.

We thank Reviewer #2 for evaluating our manuscript in a thoroughly manner and we support involving Early Career Researchers in the review process.

Reviewer #3

The authors of the manuscript presented a novel catalyst design concept to enhance the productivity of C3-C5 alkenes in unidimensional 10-membered ring zeolites (ZSM-22 and ZSM-48) for MTH reaction by simply mixing the zeolite component with a secondary catalyst component (Pd/SiO2) under near ambient pressure with H2 co-feeding. The authors ascribed such performance enhancement to the coke resistance induced by SCC for the hydrogenation of coke precursors (formaldehyde, dienes, etc.). The proposed bifunctional catalyst design strategy is interesting and the experiments were systematically performed. The manuscript may be published after the following major issues being well addressed.

We thank Reviewer #3 for acknowledging the novelty of our multicomponent catalyst and the experimental approach. We have addressed their comments and concerns below.

1) Several unidimensional 10-membered ring zeolites (ZSM-22 and ZSM-48) were employed to integrate SCC (Pd/SiO2) in order to inhibit the formation of coking and improve the lifetime of the catalysts. The unidimensional 10-membered ring parent zeolites suffer seriously from fast coking formation and deactivation, and they are seldom employed for the practical MTH reaction. It is possible to enhance the selectivity to higher alkenes just using parent ZSM-5 samples with high durable stability and lifetime. Why the authors did not test the integration of ZSM-5 samples with SCC? It would be more important and industrially practical for the MTH reaction.

Reviewer #3 is perfectly right that 10 MR are highly prone to coke-induced deactivation, exhibiting 2-3 orders of magnitude faster deactivation relative to the state-of-the-art ZSM-5-based MTH catalysts. Nonetheless, these zeolites offer an exceptional selectivity to medium-range C_{3+} alkenes (80% carbon-based selectivity), while strongly suppressing the formation of aromatics and ethylene. This product mixture is particularly suitable for direct upgrading via oligomerization-hydrogenation steps into sustainable aviation fuels (SAFs). Although an industrial deployment in this direction is still pending, several major companies have already licensed methanol-to-jet technologies (see e.g., Topsoe's technology MTJet™ (https://www.topsoe.com/solutions/technologies/process-licensing/mtjet-process, Honeywell's eFining™ technology (https://uop.honeywell.com/en/industry-solutions/renewable-fuels/efining, Exxon's Methanol to Jet technology (https://www.exxonmobilchemical.com/en/resources/library/library-detail/101116/exxonmobil_sustainable_aviation_fuel_production_en) and the relevant patents mention the 10 MR zeolites as preferable catalysts (see e.g., WO2022063994A1). Thus, our motivation to study MCC systems based on 10-MR zeolites stems from the prospect that improving the stability of catalysts with such a favourable product distribution could substantially advance their practical deployment, while also providing a fundamental strategy for enhancing the coking resistance of similarly highly selective yet inherently low-stability systems. Nonetheless, following the Reviewer's suggestion, and to corroborate the generality of our approach, **we have extended the MCC approach to include MFI zeolites**. A net lifetime improvement is also observed for the ZSM-5-based MCC relative to the parent zeolite, although the enhancement factor is appreciably lower than that observed for 1D zeolites, being 1.5×. **These results are presented in the revised Supplementary Information file (please**

see Supplementary Fig. 12), and in the revised manuscript (please see page: 13, lines: 5-11): "This value is in the range of those achieved using the industrially deployed ZSM-5 zeolite (**Supplementary Fig. 12**), illustrating the potential of the MCC approach to attain industrially attractive catalyst stability with 1D zeolite topologies. Furthermore, as demonstrated with Pd/SiO_2-ZSM-5 MCC system, which showed an enhancement factor of 1.5× compared to already high CT_0 value of parent ZSM-5 catalyst (**Supplementary Fig. 13**), the MCC approach can be extended beyond 1D 10-MR zeolites.**"**

2) The authors stated that the effect of SCC primarily arises from intercepting coke-forming pathways through the hydrogenation of coke precursors (CH2O, dienes, etc.). Why the hydrogenation of alkenes to alkanes can be inhibited on SCC? What is the difference between the hydrogenation activities of alkenes, dienes, and CH2O on SCC after induction?

We thank Reviewer #3 rising this comment, which is **now addressed within the response to comment #6 of Reviewer #1**.

3) The cyclopentenyl species was identified as the active HCP intermediates in ZSM-22. What are the dominating HCPs in ZSM-22? The authors have suggested that the olefinic cycle dominates the MTH reaction in ZSM-22. It would be better to differentiate the "active" and the "dominating" HCPs in different zeolites for methanol conversion.

We thank Reviewer #3 for spotting a possible semantic pitfall. As Reviewer #3 implicates in concluding remark, we make a distinction between active and dominating species. During MTH over ZSM-22 zeolite, the alkene cycle is dominating, with alkene species being the main chain carriers, as probed by ^{13}C incorporation experiments (see *e.g.*, Z. Liu et al. *RSC Adv.* 2016, *6*, 95855-95864). In this regard, alkenes (or respective carbenium ions) could be a reasonable choice to normalize the *operando* DR UV-vis signal for line profile analysis (Fig. 3). However, their spectral fingerprint (210 ±10 cm^{-1}) falls at the boundary of the spectral window. Alkyl-cyclopentenyl cations are hydrocarbon intermediates pinpointed during MTH within ZSM-22 as well as in other zeolites. They are considered to be active species (*i.e.* reactive and not accumulated), readily formed from alkenes under reacting conditions, which can revert to alkenes or evolve into aromatics. We refer, among the others, to the spectroscopic work of F.C. Jentoft et al. (*ACS Catal.* 2021, *11*, 12893–12914, already cited within the manuscript), the DFT investigation by P. N. Plessow et al. (*J. Phys. Chem. C* 2025, *129*, 20971–20980), and the recent proposal of a methylcyclopentenyl-based cycle of Z. Liu et al. (*ACS Catal.* 2019, *9*, 7373–7379). **We have clarified the role of cyclopentenyl species in our revised manuscript (please see page: 15, lines: 2-6): "**This is in agreement with previous studies identifying cyclopentenyl species as active HCP intermediates in ZSM-22[36-39]. In particular, alkyl-cyclopentenyl cations are easily formed from alkene species, even in the absence of CH_3OH (**Supplementary Fig. 15**) and can in turn revert to alkenes or evolve into aromatics without accumulating.**"**

4) The authors claimed that several different [Pd] integration methods were compared with comparable Pd size distribution. However, it is 4.5 nm of the Pd/SiO_2 SCC, and 2.8 nm of the Pd loaded on ZSM-22 zeolite. Are the sizes of Pd distribution indeed comparable?

The Reviewer is right in noticing that the two particles size distribution are not perfectly identical. However, the lower and upper bounds of particle-size distributions in Pd/SiO_2 SCC and Pd/ZSM-5 fall within 2-7 nm. Additionally, we could not observe the formation of highly-dispersed "single atom" Pd sites, nor the formation of substantially larger Pd nanoparticles. **We have accordingly amended our statement in the revised manuscript, page: 9, lines: 15-17): "**[…] and *iii*) a bifunctional Pd/ZSM-22 catalyst (**Fig. 1f**, **Supplementary Figs. 1** and **5**), comprising Pd loaded on a ZSM-22 zeolite and exhibiting Pd particles within a similar size range (d_{Pd} ≈ 2.8 nm), […]**"**

5) Why $W_{zeolite}$:$W_{Pd/SiO2}$ = 1:2 for ZSM-48, and 1:1 for ZSM-22? Why different ratios were taken?

On moving from ZSM-22 to ZSM-48, all the experimental conditions where kept the same, including the initial amount of SCC used, except for the amount of zeolite catalyst, which was reduced by half to account for the higher activity and conversion capacity of ZSM-48 zeolite with respect to ZSM-22 (as assessed for the parent zeolites). As a result, the W_{SCC}:$W_{zeolite}$ ratio is increased to 2:1 for ZSM-48. However, it should be noted that when studying the effect of the W_{SCC}:$W_{ZSM\text{-}48}$ ratio over the cumulative olefin productivity (**Fig. 2**), the ratio was varied from 0.2:1 to 1:2, which covers the 1:1 ratio used in the tests with ZSM-22. We further stress that irrespective of the ratio used, the enhancement effect is always observed, with a functional dependence as shown in **Fig. 2**.

6) Why the influence of the loading of SCC was tested on ZSM-48, not on ZSM-22? Which one is the primary parent sample studied in this work?

Both zeolites, ZSM-22 and ZSM-48, are equally important. Since the purpose of our communication is to report the MCC approach in general, irrespective of the specific sample, we chose only one to show the effect of SCC loading.

Manuscript NCOMMS-25-86258A – Response to Reviewers

Notes: Comments in *blue* - Replies in black - Actions in **bold**

Indicated page, line, figure, or reference numbers refer to the revised Manuscript and/or Supporting Information with changes highlighted.

Reviewers #2 and #3

I co-reviewed this manuscript with one of the reviewers who provided the listed reports. This is part of the Nature Communications initiative to facilitate training in peer review and to provide appropriate recognition for Early Career Researchers who co-review manuscripts. I am satisfied with the revisions and recommend the acceptance of the revised manuscript.

Thank you for your review and recommendation. We appreciate your assessment and are pleased that the revisions have satisfactorily addressed the concerns raised. We thank you for your time and valuable feedback throughout the review process.

The authors of the manuscript presented a novel catalyst design concept to enhance the productivity of C3-C5 alkenes in unidimensional 10-membered ring zeolites (ZSM-22 and ZSM-48) for MTH reaction by simply mixing the zeolite component with a secondary catalyst component (Pd/SiO2) under near ambient pressure with H2 co-feeding. The authors ascribed such performance enhancement to the coke resistance induced by SCC for the hydrogenation of coke precursors (formaldehyde, dienes, etc.). The proposed bifunctional catalyst design strategy is interesting and the experiments were systematically performed. The manuscript may be published after the following major issues being well addressed.

1) Several unidimensional 10-membered ring zeolites (ZSM-22 and ZSM-48) were employed to integrate SCC (Pd/SiO2) in order to inhibit the formation of coking and improve the lifetime of the catalysts. The unidimensional 10-membered ring parent zeolites suffer seriously from fast coking formation and deactivation, and they are seldom employed for the practical MTH reaction. It is possible to enhance the selectivity to higher alkenes just using parent ZSM-5 samples with high durable stability and lifetime. Why the authors did not test the integration of ZSM-5 samples with SCC? It would be more important and industrially practical for the MTH reaction.

2) The authors stated that the effect of SCC primarily arises from intercepting coke-forming pathways through the hydrogenation of coke precursors (CH2O, dienes, etc.). Why the hydrogenation of alkenes to alkanes can be inhibited on SCC? What is the difference between the hydrogenation activities of alkenes, dienes, and CH2O on SCC after induction?

3) The cyclopentenyl species was identified as the active HCP intermediates in ZSM-22. What are the dominating HCPs in ZSM-22? The authors have suggested that the olefinic cycle dominates the MTH reaction in ZSM-22. It would be better to differentiate the “active” and the “dominating” HCPs in different zeolites for

methanol conversion.

4) The authors claimed that several different integration methods were compared with comparable Pd size distribution. However, it is 4.5 nm of the Pd/SiO2 SCC, and 2.8 nm of the Pd loaded on ZSM-22 zeolite. Are the sizes of Pd distribution indeed comparable?

5) Why $W_{zeolite}$:$W_{Pd/SiO2}$ = 1:2 for ZSM-48, and 1:1 for ZSM-22? Why different ratios were taken?

6) Why the influence of the loading of SCC was tested on ZSM-48, not on ZSM-22? Which one is the primary parent sample studied in this work?
